# Programmable RNA targeting by bacterial Argonaute nucleases with unconventional guide binding and cleavage specificity

**DOI:** 10.1038/s41467-022-32079-5

**Published:** 2022-08-08

**Authors:** Lidiya Lisitskaya, Yeonoh Shin, Aleksei Agapov, Anna Olina, Ekaterina Kropocheva, Sergei Ryazansky, Alexei A. Aravin, Daria Esyunina, Katsuhiko S. Murakami, Andrey Kulbachinskiy

**Affiliations:** 1 Institute of Molecular Genetics, National Research Center “Kurchatov Institute”, Moscow, Russia; 2 Institute of Gene Biology, Russian Academy of Sciences, Moscow, Russia; 3 Department of Biochemistry and Molecular Biology, Pennsylvania State University, University Park, PA USA; 4 Division of Biology and Biological Engineering, California Institute of Technology, Pasadena, CA USA; 5 Present Address: Department of Biochemistry and Molecular Biophysics, Columbia University, New York, NY USA

**Keywords:** RNAi, CRISPR-Cas systems, X-ray crystallography

## Abstract

Argonaute proteins are programmable nucleases that have defense and regulatory functions in both eukaryotes and prokaryotes. All known prokaryotic Argonautes (pAgos) characterized so far act on DNA targets. Here, we describe a new class of pAgos that uniquely use DNA guides to process RNA targets. The biochemical and structural analysis of *Pseudooceanicola lipolyticus* pAgo (PliAgo) reveals an unusual organization of the guide binding pocket that does not rely on divalent cations and the canonical set of contacts for 5’-end interactions. Unconventional interactions of PliAgo with the 5’-phosphate of guide DNA define its new position within pAgo and shift the site of target RNA cleavage in comparison with known Argonautes. The specificity for RNA over DNA is defined by ribonucleotide residues at the cleavage site. The analysed pAgos sense mismatches and modifications in the RNA target. The results broaden our understanding of prokaryotic defense systems and extend the spectrum of programmable nucleases with potential use in RNA technology.

## Abstract (web summary)

The authors describe programmable Argonaute nucleases that use small DNA guides to recognize and cleave RNA targets and can sense mismatches and modifications in RNA targets, making them potential tools for RNA biotechnology.

## Introduction

Two principal types of programmable nucleases, CRISPR-Cas (clustered regularly interspaced short palindromic repeats, CRISPR-associated proteins) and Ago (Argonaute) proteins, play the central role in genetic immune systems that protect host cells from invader nucleic acids^1–3^. Both of them use small nucleic acid guides to recognize and cleave their targets. Prokaryotic CRISPR-Cas systems utilize RNA guides transcribed from the CRISPR cassettes to recover memory of previous infections and destroy invader DNA or RNA^4–6^. Eukaryotic Argonaute proteins (eAgos) play the central role in RNA interference (RNAi) and use guide RNAs for the recognition of RNA targets^7,8^. They can be loaded with small RNAs processed from viral RNA or transcribed from genomic loci, to target viral RNA genomes, inhibit transposon expression, or participate in gene regulation^9,10^. In contrast, most studied prokaryotic Ago (pAgo) nucleases have a natural specificity for DNA guides and DNA targets^11–20^, and a small group of CRISPR-associated pAgos has a preference for RNA guides and DNA targets^21,22^. Some of the studied pAgos were also shown to bind and cut RNA targets in vitro, but with a lower rate than DNA^19–21,23–25^. No pAgo with a distinct preference for RNA targets was described.

The preferential specificity of studied pAgos to DNA targets may be connected to the abundance of DNA viruses and plasmids in prokaryotes, which are the main targets for prokaryotic immune systems. Indeed, CbAgo from *Clostridium butyricum* was recently demonstrated to provide defense against bacteriophages^26^, while several pAgos including CbAgo and TtAgo from *Thermus thermophilus* were shown to target plasmid DNA^15,16,18,26,27^. As was demonstrated for CbAgo, foreign genetic elements may be recognized based on their intense replication and the absence of species-specific Chi motifs recognized by the host double-strand break machinery (RecBCD in *E. coli*), thus making them a preferential source of small guide DNAs and a target for pAgo-dependent degradation^26^. CRISPR-Cas systems may rely on similar mechanisms for protospacer selection during adaptation to foreign genetic elements^28–30^, and may possibly cooperate with pAgos in fighting phage infections^26^.

Typical Ago proteins contain six main domains, N-terminal, L1 (Linker 1), PAZ (Piwi-Argonaute-Zwille), L2 (Linker 2), MID (Middle) and PIWI (P-element induced wimpy testis), responsible for guide binding and target recognition^3,8,31,32^. The PIWI domain contains the nuclease active site with a catalytic tetrad (DEDX motif, where X is D, H, or K) that coordinates two divalent metal cations essential for target cleavage. The MID domain forms a basic pocket that accommodates the 5’-end of guide nucleic acid through stacking and polar interactions, involving a divalent metal cation (Mg^2+^) bound in pAgos and some eAgos^17,21,32,33^. The PAZ domain binds the 3’-end of guide nucleic acid, which is released upon target recognition^24,34,35^. The first several nucleotides of the guide nucleic acid from the ‘seed’ region are pre-ordered in a helical conformation and participate in the initial target recognition. Precise guide positioning within the Ago molecule defines the position of target cleavage, which occurs between 10^th^ and 11^th^ guide nucleotides in all studied pAgos and eAgos^3,8,22,31,36^

Phylogenetic analysis revealed that pAgos are much more diverse than eAgos and include not only full-length catalytically active proteins but also multiple variants with substitutions of key residues in the catalytic tetrad and deletions of individual domains (Supplementary Fig. 1)^2,22,31,36^. For catalytically active pAgos, their catalytic tetrad was shown to be essential for DNA targeting in vitro and in vivo^12,15,16,18,21,26,37,38^. Inactive pAgos may also play a role in cell defense against invader nucleic acids, possibly in cooperation with accessory nucleases^3,22,27,31,36^. Indeed, pAgos lacking the catalytic activity are often co-encoded with nucleases, which might be involved in guide and target processing, but their functional activities remain unknown^2,22,31^.

Due to their relatively small protein size and the absence of strong requirements for specific motifs during guide binding or target recognition, pAgos have a potential to become a tool for nucleic acid manipulations and genomic technologies^3,11^. Search for the new groups of pAgos with undiscovered specificities is therefore of a great importance for not only understanding of their biological functions and evolution of RNAi but also for expanding the toolbox for biotechnology. However, despite the huge diversity of pAgos, their mechanisms of action and cellular functions are only beginning to be explored, with the previous studies limited to DNA-targeting pAgos.

In this study, we identify a new class of pAgos that are strictly specific to DNA guides and RNA targets, and perform their biochemical and structural analysis. We show that a subset of these pAgos have a shifted target cleavage site relative to the 5’-end of guide in comparison with other pAgos and eAgos. By determining the atomic resolution X-ray crystal structures of a pAgo from this class in complex with guide DNA, we demonstrate that these pAgos have a novel type of the MID binding pocket resulting in different positioning of guide DNA and explaining the observed cleavage preferences. The new type of programmable nucleases are active under physiological temperature and may serve as a potential tool for RNA biotechnology, including sequence-specific detection of RNA and transcriptome manipulations.

## Results

### A novel class of pAgos use DNA guides to process RNA targets

Biochemical and structural studies of a limited number of pAgos from several bacterial and archaeal species revealed that most of them have preference for DNA guides and DNA targets and some for RNA guides and DNA targets (see Introduction). However, comprehensive phylogenetic analysis of pAgo sequences from prokaryotic genomes revealed their remarkable diversity^22,31^, suggesting that proteins with different properties might exist. To find pAgos with novel specificities, we studied the activities of pAgos from previously unexplored branches of their phylogenetic tree.

**Fig. 1:**
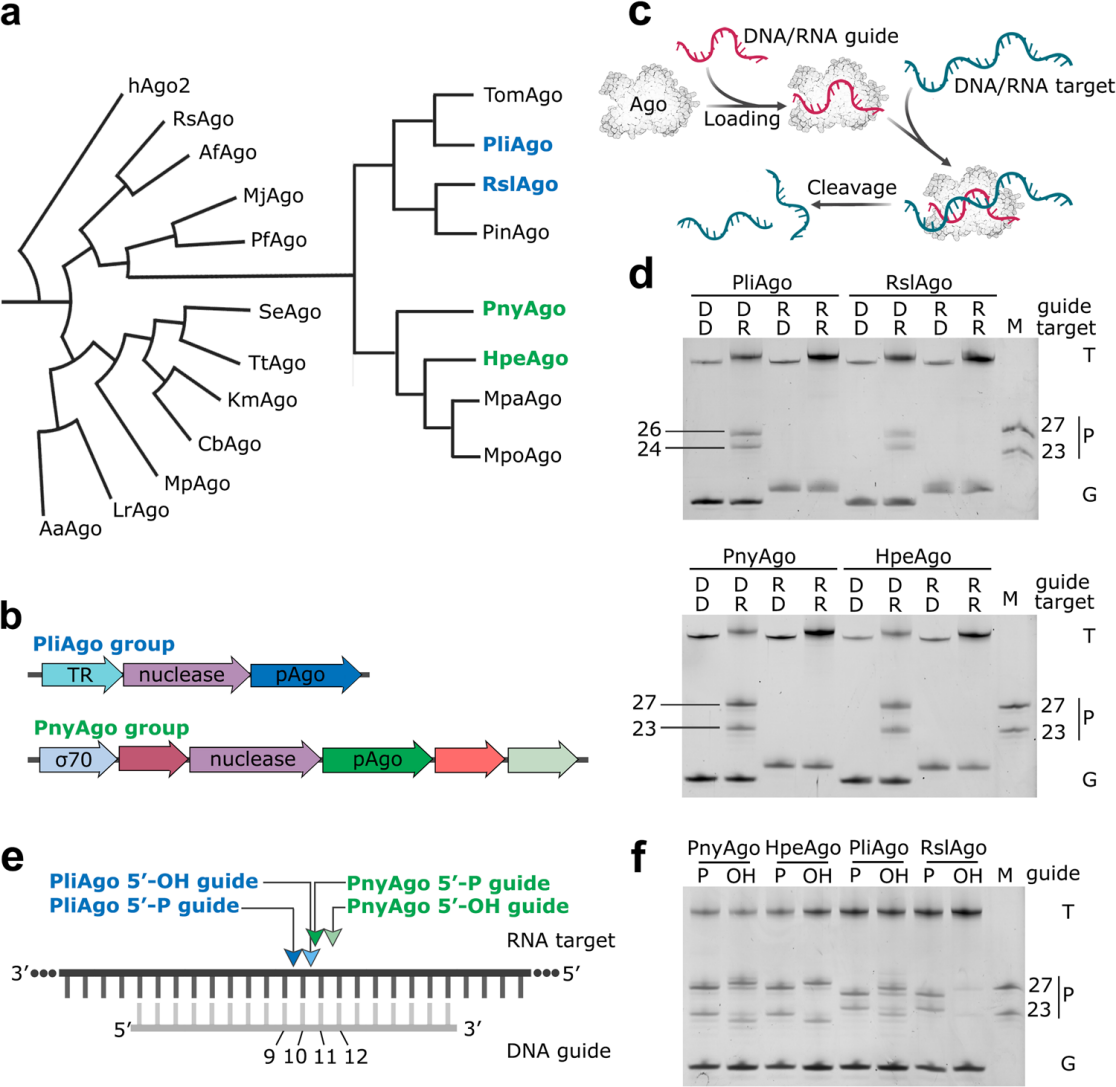
Two groups of pAgos prefer DNA guides and RNA targets. **a** Phylogenetic tree of previously studied pAgos and of predicted D-R pAgos; pAgos studied in this work are shown in color (see Supplementary Fig. 1a for the full list of D-R pAgos and abbreviations). **b** Operon structure of D-R pAgos from the PliAgo (top) and PnyAgo (bottom) groups. pAgo-associated nuclease from the PD-(D/E)XK superfamily, putative transcription regulator (TR) and σ^70^ family factor are indicated. **c** Scheme of the cleavage assay. **d** Nucleic acid specificity of the two groups of pAgos. The reactions were performed with DNA (‘D’) or RNA (R’) guides and targets; positions of targets (‘T’), guides (‘G’) and reaction products (‘P’) are indicated (SYBR Gold staining). **e** Schematics of target RNA cleavage by the two groups of D-R pAgos in the presence (dark arrowheads) and in the absence (light arrowheads) of the 5’-phosphate in guide DNA. Nucleotide positions in guide DNA are indicated. **f** Analysis of RNA cleavage by D-R pAgos with 5′-P and 5′-OH guide DNAs. In panels **d** and **f** representative gels from 3 independent experiments with similar results are shown.

Using sequence analysis of the catalytic PIWI and MID domains^22,26^, we have identified two related groups of long pAgos that were not studied previously and that harbor proteins with predicted nuclease sites and unusual MID-pockets (Fig. 1a and Supplementary Fig. 1; see also Fig. 3a below). In total, we identified 14 and 13 pAgos in each group, among the 1,711 pAgos previously found in 2,883 prokaryotic genomes (Supplementary Fig. 1a). Similar pAgo genes that belong to these two groups are found in unrelated bacteria from different classes of Proteobacteria, Bacteroidetes, Planctomycetes, Chloroflexi and Cyanobacteria (Supplementary Fig. 1a), suggesting their horizontal transfer between species. Analysis of gene neighbors of these pAgos revealed that in each group pAgos are encoded in operons with the same gene composition, suggesting functional association between pAgos and adjacent genes. Remarkably, in both groups the pAgo gene is preceded by a gene encoding a nuclease from the PD-(D/E)XK superfamily, distantly related to Cas4 (Fig. 1b). In addition, pAgos from the first group are encoded together with a putative helix-turn-helix transcriptional regulator, while pAgos from the second group are accompanied by a σ^70^ family RNA polymerase σ factor (Fig. 1b).

We selected four pAgo proteins from these two groups for further characterization, PliAgo from *Pseudooceanicola lipolyticus* and RslAgo from *Runella slithyformis* from the first group, and PnyAgo from *Pedobacter nyackensis* and HpeAgo from *Hydrobacter penzbergensis* from the second group (Fig. 1a). We cloned the genes encoding these pAgos in a protein expression vector, expressed and purified these proteins from *E. coli* (Supplementary Fig. 2a), and analyzed their nuclease activities in an in vitro nucleic acid cleavage assay (Fig. 1c). All four proteins were shown to act as programmable nucleases that use DNA guides to process RNA targets and are not active with other combinations of guides and targets (Fig. 1d). This type of strict guide and target specificities has never been identified for any other programmable nuclease. Weak cleavage of DNA targets with DNA guides can be observed at long incubation times only with PnyAgo, but the rate of this reaction is much lower compared to cleavage of target RNA (Supplementary Fig. 2b). Sequence alignments showed that all four proteins have the canonical catalytic tetrad DEDD in their PIWI domains (D563, E592, D626 and D762 in PliAgo; D563, E598, D632 and D765 in PnyAgo; Supplementary Fig. 1b). No target cleavage is observed with catalytically inactive variants of PliAgo and PnyAgo with substitutions of the first and third residues of the DEDD motif (Supplementary Fig. 2c).

These results show that the newly identified pAgos belong to a novel class of programmable nucleases that use DNA guides to recognize and cleave RNA targets. We designate this group of nucleases as D-R pAgos, in comparison with D-D pAgos and R-D pAgos that use DNA or RNA guides, respectively, to cleave DNA targets (in this classification, all eukaryotic Argonautes are R-R Agos).

Strikingly, the site of RNA cleavage by PliAgo and RslAgo from the first group of pAgos is shifted in comparison with PnyAgo and HpeAgo from the second group, as well as with previously studied Ago proteins from both prokaryotes and eukaryotes. While the canonical cleavage site is located between target positions 10′ and 11′, corresponding to the 10^th^ and 11^th^ nucleotide from the guide 5′-end, RNA cleavage by PliAgo and RslAgo primarily occurs between positions 9′ and 10′, one nucleotide closer to the guide 5′-end (Fig. 1d, e, Supplementary Fig. 2d). This class of pAgo proteins may therefore utilize an unusual mechanism for guide binding and/or target recognition.

All four tested D-R pAgos from both groups can also utilize guide DNAs lacking a phosphate group in their 5′-ends, unlike the majority of previously studied pAgos (Fig. 1f and Supplementary Fig. 2e). However, in this case the major site of RNA cleavage is shifted toward the guide 3′-end, resulting in preferential cleavage between target positions 10’ and 11’ in the case of PliAgo and RslAgo, and between positions 11’ and 12’ in the case of PnyAgo and HpeAgo (Fig. 1). The 5’-phosphate group in guide DNA therefore affects the selection of the cleavage site by D-R pAgos.

To define the range of conditions compatible with RNA cleavage by the D-R pAgos, we tested the effects of temperature, divalent cations and the structure of guide molecules on the cleavage efficiency (Supplementary Fig. 3). All four pAgos have the highest activity between 30 and 44 °C and are inactivated at elevated temperatures above 50 °C (Supplementary Fig. 3a, e). Accordingly, they are encoded by mesophilic bacteria. The reaction rates for these pAgos at 37 °C are comparable to the cleavage rates previously reported for other groups of pAgos (the half-reaction times vary between 5.4 min for HpeAgo and 34.6 min for PliAgo) (Supplementary Fig. 3b). All four proteins are active with Mg^2+^ and Mn^2+^, but not with other tested divalent cations (Ca^2+^, Co^2+^, Cu^2+^ or Zn^2+^). In addition, PliAgo has a low level of activity with Co^2+^ (Supplementary Fig. 3c, f). The pattern of the RNA cleavage products is similar for Mg^2+^ and Mn^2+^-dependent reactions (Supplementary Fig. 3f). Similarly, previously studied pAgos, as well as other enzymes catalyzing phosphoryl transfer reactions, were shown to be mostly active with Mg^2+^ and Mn^2+^^12,15,16,21,39^^–^^41^. The maximum activity is observed with 14-20 nt guides for PliAgo and RslAgo from the first group and with 16-20 nt guides for PnyAgo and HpeAgo from the second group (Supplementary Fig. 3d, g). The cleavage efficiency drops dramatically with guides shorter than this range for pAgos from both groups. For PliAgo and RslAgo, additional shorter cleavage products are observed with guides longer than 18 nt (Supplementary Fig. 3g), suggesting that the optimal guide length is important for precise target cleavage. At the same time, the cleavage site selection is not affected by the temperature, the divalent cation cofactor and the ionic strength of the reaction (Supplementary Fig. 3e–h). The efficiency and precision of target RNA cleavage also does not depend on the identity of the 5’-nucleotide in guide DNA (with the exception of RslAgo, the activity of which is higher with 5’-G guide DNA) (Supplementary Fig. 2e), which is known to be specifically recognized by other Ago proteins^16,17,27,42^.

### 3D structures of PliAgo and its complexes with guide DNA

To elucidate the mechanism of guide DNA binding and cleavage site selection by the newly identified D-R pAgos, we determined 3D structures of apo-form PliAgo and of its complex with 5’-phosphorylated guide DNA (P-gDNA, 18 nt long), either in the absence or in the presence of Mg^2+^. To decipher the functional role of the guide 5’-phosphate, we also solved the structure of PliAgo with non phosphorylated guide DNA (OH-gDNA). All crystals were formed at nearly identical crystallization conditions and belonged to the same space group, containing one PliAgo or PliAgo-DNA complex per asymmetric unit. The structure of PliAgo was determined by single wavelength anomalous dispersion (SAD) method with SeMet labeled protein at 3.28 Å resolution followed by extending the final resolution to 3.1 Å with a native crystal. The structures of PliAgo and DNA complexes (~2.5 Å resolution) were determined by molecular replacement using the apo-form PliAgo structure as a search model (Supplementary Table 2).

**Fig. 2:**
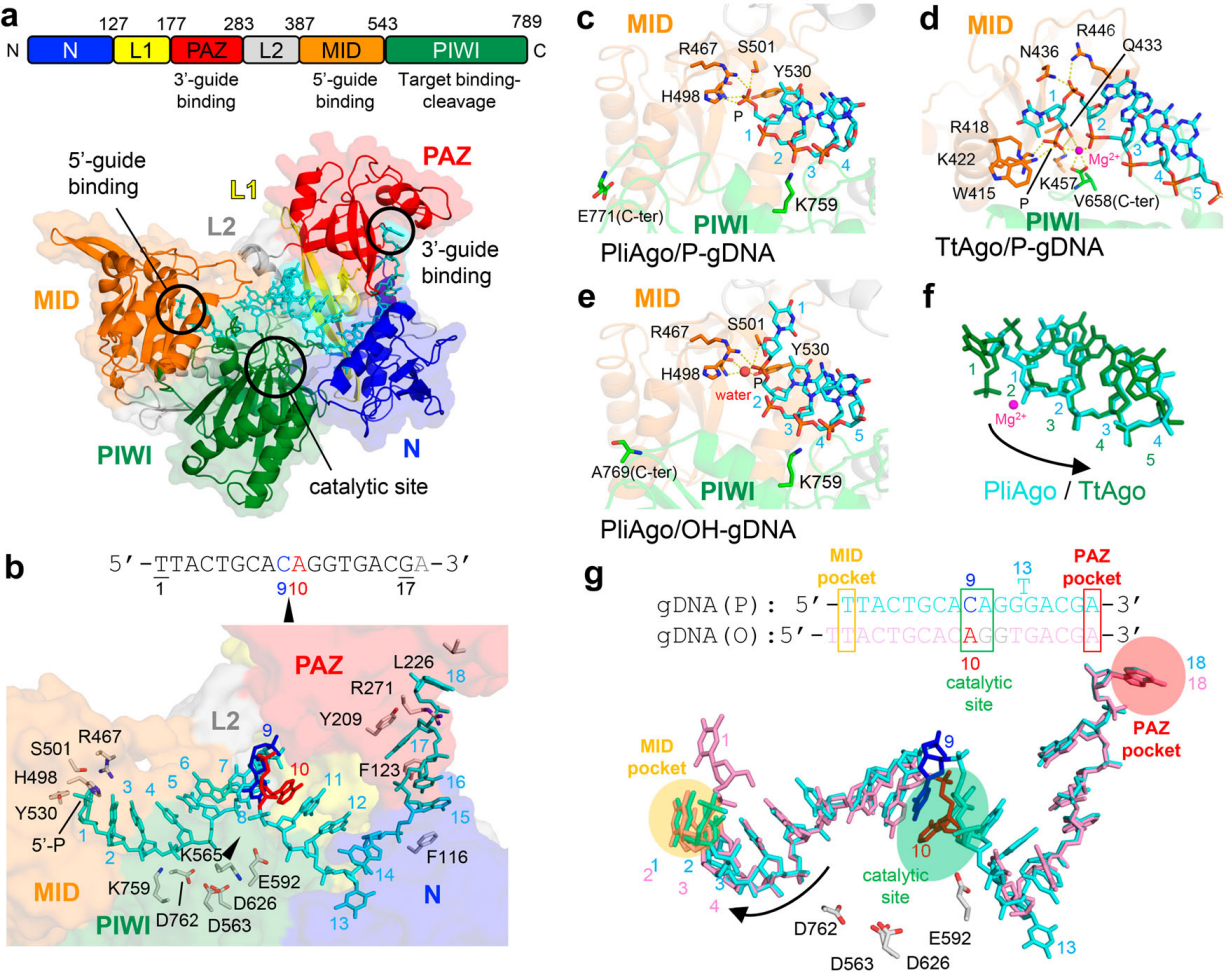
Structure of PliAgo and its interactions with guide DNA. **a** The structure of the PliAgo and P-gDNA complex. (*Top*) The primary structure of PliAgo with amino acid numbering. Domains and their functions are colored and labeled. (*Bottom*) Overall structure of the PliAgo-P-gDNA complex. PliAgo and DNA are depicted as ribbon and stick models, respectively, with partially transparent surfaces. Domains are colored as in the top panel and labeled. **b** The PliAgo and P-gDNA interactions. (*Top*) The guide DNA sequence used for complex crystallization. Nucleotides that define the site of target RNA cleavage are colored. (*Bottom*) A magnified view of the PliAgo-P-gDNA interactions. PliAgo is depicted as a partially transparent surface. DNA nucleotides and amino acid residues responsible for guide positioning are shown as stick models and labeled. The catalytic site is indicated as a black triangle. The view is the same as **a**. **c**–**e** The guide 5’-end binding in the PliAgo-P-gDNA complex **c** TtAgo-P-gDNA complex (PDB: 3DLH^32^) **d**, and PliAgo-OH-gDNA complex **e**. PliAgo and TtAgo are depicted as partially transparent ribbon models. Amino acid residues responsible for 5’-end binding are shown as stick models and labeled. Salt bridges between amino acid residues and phosphate groups as well as coordination bonds of Mg^2+^/water are shown as yellow dashed lines. **f** Overlay of guide DNAs in the PliAgo-P-gDNA (cyan) and TtAgo-P-gDNA complexes (green). Positions of guide DNA nucleotides are labeled. Shifting the register of guide DNA from TtAgo to PliAgo is indicated by a black arrow. **g** 5’-phosphate dependent guide DNA positioning by PliAgo. (*Top*) The registers of 5’-P and 5’-OH guide DNAs in the PliAgo-gDNA complexes. (*Bottom*) Overlay of the guide DNA in the complexes of PliAgo with P-gDNA (cyan) and OH-gDNA (pink). Shifting the register of guide DNA without the 5’-phosphate group is indicated by a black arrow.

A crescent-shaped PliAgo molecule contains the N-terminal-L1-PAZ domains on one side and the L2-MID-PIWI domains on the other side (Fig. 2a, Supplementary Fig. 4a–c), similar to other eAgos and pAgos such as TtAgo (Supplementary Fig. 4d)^14,24,25,32^. The structure of the binary complex of PliAgo with P-gDNA shows the path of the DNA molecule and its contacts with the MID, PIWI, N and PAZ domains of the protein (Fig. 2 and Supplementary Fig. 4a). The positions of DNA bases in the seed and the 3’ supplemental regions are well determined whereas the bases in the cleavage region (positions 9-12 in the P-gDNA, 10-13 in the OH-gDNA) are not well determined likely due to their intrinsic flexibility before base-paring with their target RNAs (Supplementary Fig. 4b, c). The RNaseH fold found in the PIWI domain forms the catalytic site with the tetrad residues D563, E592, D626 and D762 located near the expected cleavage site on target RNA. The 9^th^ and 10^th^ nucleotides of P-gDNA, which define the cleavage site on target RNA, are positioned in front of the catalytic site of PliAgo (Fig. 2a, b). Along its length, guide DNA is oriented within PliAgo through multiple contacts between the DNA backbone and positively charged amino acid residues. These contacts pre-order guide DNA in a nearly A-form conformation from the 1^st^ to 6^th^ nucleotides in the seed region (Supplementary Fig. 4d), thus making it optimal for target RNA recognition. In comparison, the seed region of the guide strand in available structures of hAgo2 (that targets RNA) or in other pAgos (that naturally target DNA) also adopts the A-form conformation or intermediate conformations between the A- and B-forms, even in DNA-targeting pAgos, suggesting that the nucleic acid topology is primarily dictated by the protein interactions (Supplementary Fig. 4e)^14,21,25,34,43–45^.

Similarly to previously studied binary complexes of Ago proteins^17,21,32^, the 5′- and 3′-ends of P-gDNA are accommodated in pockets formed by the MID and PAZ domains of PliAgo, respectively (Fig. 2b). PliAgo recognizes the 5′-phosphate of guide DNA by salt bridges with several charged and polar residues in the MID domain, using a unique set of contacts in comparison with other pAgos and eAgos (Fig. 2c and Supplementary Fig. 4b, see below). In accordance with biochemical observations, there are no sequence-specific contacts of the 5′-end nucleotide base with PliAgo. The 3′-end guide DNA bases from the 15^th^ to 16^th^ nucleotides are positioned along the side wall of the N and PAZ domains using base stacking interactions between residues F116/F123 and the 15^th^/16^th^ nucleotides, and residue Y209 and the 17^th^ nucleotide (Fig. 2b). The 18^th^ nucleotide of gDNA is positioned in a crevice of the PAZ domain and is sandwiched between residues R271 and L226 (Fig. 2b). Notably, the DNA-protein contacts are significantly altered in the complex of PliAgo with non phosphorylated guide DNA (OH-gDNA) (Fig. 2e, g), explaining the role of the 5’-phosphate in guide DNA binding and cleavage site selection by PliAgo. The implications of these structures for the activity of PliAgo are further discussed below.

### Guide DNA binding by a novel type of the MID pocket in PliAgo

**Fig. 3:**
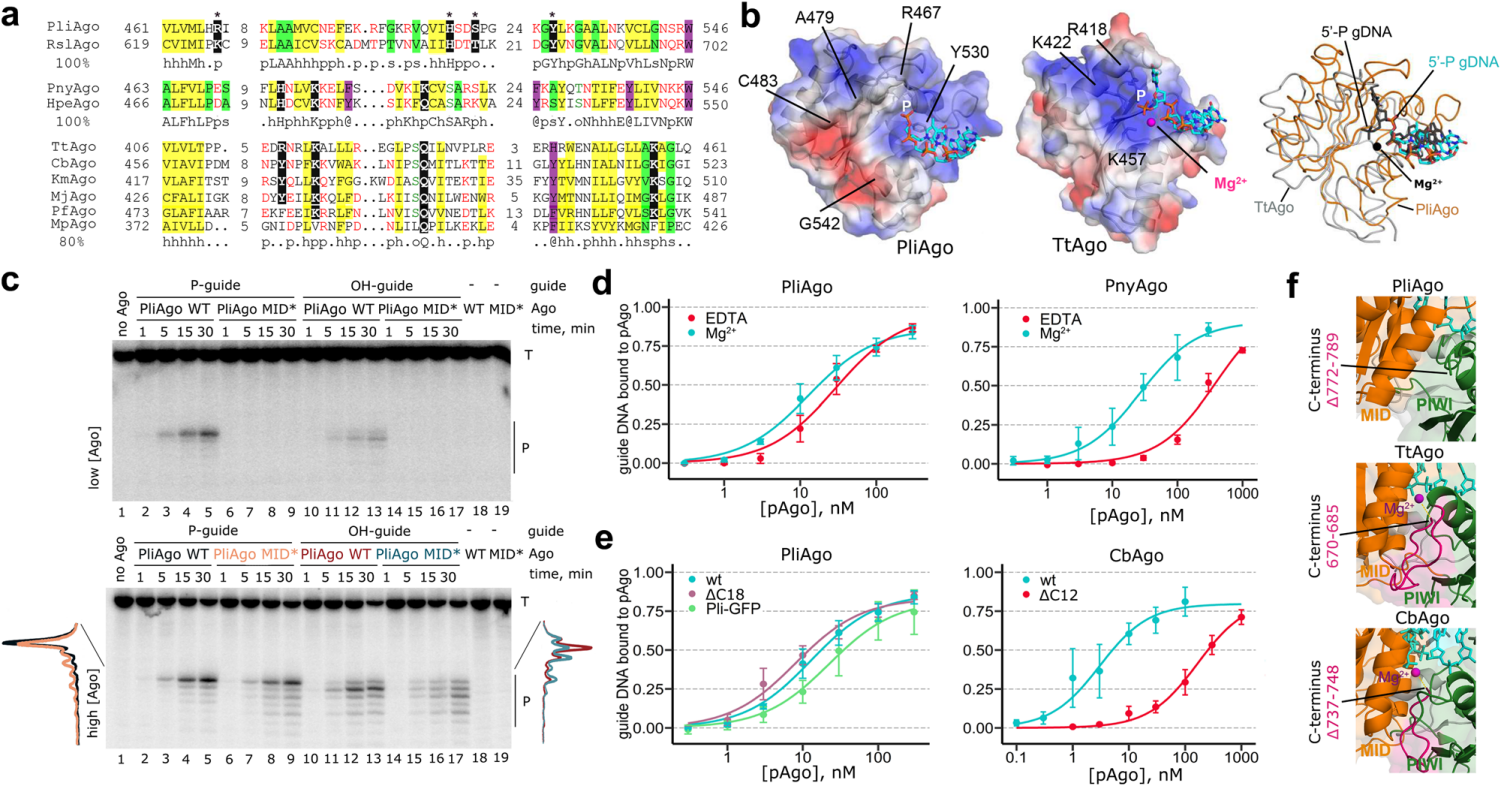
Role of the MID pocket in guide DNA binding and target RNA cleavage. **a** Alignment of the MID pocket in various groups of pAgos. Conserved residues of the MID motif involved in 5’-nucleotide interactions are indicated in black. MpAgo has a hydrophobic MID pocket and binds 5′-hydroxylated guide RNAs but is more closely related to canonical pAgos than to PliAgo. The consensus for each group is shown underneath the alignment: ‘h’, hydrophobic residues (WFYMLIVACTH); ‘p’, polar (EDKRNQHTS); ‘s’, small (ACDGNPSTV); ‘o’, OH-containing (ST); ‘@‘, aromatic (YWFH). **b** Interactions of the MID domain with guide 5’-end (stick) in PliAgo (*left*) and TtAgo (PDB: 3DLH^32^) (*middle*). Electrostatic potentials are shown as surfaces (blue, basic; red, acidic; white, neutral). (*Right*) Overlay of the MID pockets of PliAgo/P-gDNA (MID, orange; DNA, cyan) and TtAgo/gDNA complexes (MID, gray; DNA and Mg^2+^, black). **c** Kinetics of RNA cleavage by wild-type and MID* PliAgo (R467A/Y530A) loaded with 5’-P or 5’-OH gDNA, at low (50 nM) or high (500 nM) PliAgo concentrations (representative gel from two independent experiments). The reactions were performed with 5’-P^32^-labeled target RNA; positions of the target (‘T’) and the reactions products (‘P’) are indicated. The profiles of the cleavage products observed after 30 min with 500 nM PliAgo are shown on the left for P-gDNA (WT PliAgo, black; MID* PliAgo, orange) and on the right for OH-gDNA (WT, red; MID*, cyan). **d** Guide DNA binding by PliAgo and PnyAgo in the presence and in the absence of Mg^2+^. **e** Guide DNA binding by PliAgo, CbAgo and their variants PliAgo ΔC18 (deletion of the C-terminal 18 residues), PliAgo-GFP (C-teminal fusion with GFP), CbAgo ΔC12 (deletion of the C-terminal 12 residues). In **d** and **e**, means from 3 or 4 independent experiments are shown; error bars correspond to standard deviations. The amount of bound DNA is shown as a fraction of total 5’-P^32^-labeled DNA in the sample. **f** Comparison of the C-terminus positions in the structures of PliAgo (*top*), TtAgo (3DLH, *middle*) and CbAgo (6QZK, *bottom*). The C-terminal segment deleted in PliAgo (Δ772-789) is not solved on the structure. The corresponding segments in TtAgo (670-685) and CbAgo (737–748) are shown in magenta based on structural alignment.

When compared with previously studied pAgos and eAgos, PliAgo reveals striking differences in its interactions with the 5’-end of guide DNA. The MID domain of all previously characterized pAgos and eAgos makes extensive contacts with the 5′-phosphate group of the guide molecule through a conserved (Y/H/R)KQK motif of the MID pocket (R^418^K^422^Q^433^K^457^ in TtAgo or Y^529^K^533^Q^545^K^570^ in human Ago2) (Fig. 2d)^32,42^. Alignment of the sequences of the MID domains in D-R pAgos shows that pAgos from the group of PnyAgo have a variant of the standard MID pocket motif (Fig. 3a). In contrast, none of these residues is present in PliAgo and other pAgos from its group (Fig. 3a, Supplementary Fig. 1c). The 3D structure of PliAgo in complex with P-gDNA reveals that the 5′-phosphate group is coordinated by residues R467, H498, S501 and Y530, resulting in positioning of the 5′-end DNA base (1T) next to the side chain of Y530 (Figs. 2c and  3b and Supplementary Fig. 4b). Two of these residues (the second histidine and the forth tyrosine) are absolutely conserved in the PliAgo group of D-R pAgos (Supplementary Fig. 1c).

The orientation of the guide 5′-end in the complex of PliAgo with P-gDNA differs from all previously characterized Ago-guide complexes, shifting the register of guide DNA by one nucleotide toward the guide 3′-end. In TtAgo, which belongs to D-D pAgos and cleaves target DNA between positions 10′ and 11′^14,25,32^, guide DNA is sharply bent between positions 1 and 2, with the first nucleotide unstacked from the second and positioned in the MID pocket (Fig. 2d). In contrast, in PliAgo the 5’-end nucleotide stays outside of the MID pocket and forms stacking interactions with the second nucleotide of guide DNA (Fig. 2c, Supplementary Fig. 4b). As a result, the 1^st^ and subsequent nucleotides in guide DNA in the PliAgo complex superimpose with the 2^nd^ and subsequent guide nucleotides in previously studied pAgos (Fig. 2f). This explains the observed shift in the cleavage site position relative to the 5′-end of guide DNA in PliAgo in comparison with other Ago proteins (Fig. 1e, f).

In the complex of PliAgo with OH-gDNA, the DNA molecule slides one nucleotide upstream relative to its position in the complex with the 5’-phosphate group, with the MID pocket facing a bridging phosphate between the first and the second positions of guide DNA (Fig. 2e, g). The bridging phosphate interacts with the residues H498 (main chain) and Y530 (side chain) via a water molecule (Fig. 2e, Supplementary Fig. 4c), instead of direct coordination between the 5’-phosphate and H498 in the PliAgo-P-gDNA complex (Fig. 2c, Supplementary Fig. 4b). At the same time, similarly to the complex of PliAgo with P-gDNA, the PAZ domain interacts with the last (18^th^) nucleotide of guide DNA (Fig. 2g). The change provides the structural basis for shifting the RNA cleavage site with guides lacking the 5’-phosphate for D-R pAgos (Fig. 1e, f, Supplementary Fig. 2e).

To decipher the role of the observed protein–DNA interactions in guide binding by D-R pAgos, we determined the apparent dissociation constants (*K*_d_) for binding of guide DNA or RNA by PliAgo and PnyAgo. Both pAgos bind P-gDNA with high affinities (*K*_d_ = 14 nM for PliAgo and 31 nM for PnyAgo) (Supplementary Fig. 5a). Both proteins do not interact with guide RNA (Kd >> 1000 nM), thus explaining their observed preference for DNA guides in the cleavage reaction. Interestingly, the A-form RNA superimposes well with the seed region of guide DNA in the complex of PliAgo, with similar sugar puckering (C3′-endo conformation) and no strong clashes of the 2’-ribose OH groups with the protein (Supplementary Fig. 4d). Therefore, the exclusion of RNA from binding to PliAgo as a guide molecule may possibly be explained by differences in DNA and RNA interactions with other regions of PliAgo beyond the seed region.

We then compared the interactions P-gDNA and OH-gDNA with PliAgo and PnyAgo. *K*_d_ values for OH-gDNA are significantly increased in comparison with P-gDNA for PliAgo (>25 fold), as well as for PnyAgo (12-fold) (*p*-values of 0.0005 and 0.013, respectively) (Supplementary Fig. 5b, c). This demonstrates that changes in the OH-gDNA interactions with the MID pocket (Fig. 2e, Supplementary Fig. 4c) strongly decrease gDNA affinity to PliAgo. Consistently, the RNA cleavage activity of PliAgo loaded with 5’-OH guide is much decreased in comparison with the 5’-P guide at low PliAgo concentration (Fig. 3c, top panel, lanes 10–13). While OH-gDNA can direct efficient target RNA cleavage at high PliAgo concentration (see Fig. 1e above), the absence of the 5’-phosphate greatly affects the precision of the reaction in these conditions, as determined from analysis of radiolabeled products resulting from cleavage of a 5’-P^32^-labeled RNA target (Fig. 3c, bottom panel). In the case of P-gDNA, the major site of cleavage is located between the 9’ and 10’ target positions (~80% of all cleavage products; Fig. 3c, lanes 3–5). In contrast, in the case of OH-gDNA the main cleavage site is shifted by one nucleotide further from the guide 5′-end and accounts for <50% of all products among several other cleavage sites (Fig. 3c, lanes 11-13; compare scanned profiles of the cleavage products for P-gDNA and OH-gDNA).

To test the importance of the guide 5’-phosphate interactions in PliAgo, we obtained its derivative with substitutions of two residues involved in these interactions in the MID pocket (MID*, R467A/Y530A). The MID* variant of PliAgo has a dramatically decreased affinity for P-gDNA (*K*_d_ > 1000 nM) (Supplementary Fig. 5A). In agreement with these data, MID* PliAgo shows no catalytic activity at low pAgo concentrations (Fig. 3c, top, lanes 7–9). At a higher concentration, MID* PliAgo produces a shifted pattern of RNA products, corresponding to target RNA cleavage further downstream from the guide 5’-end in comparison with the wild-type protein (Fig. 3c, bottom, lanes 7-9; compare scanned profiles of the cleavage products for WT and MID* PliAgo). This indicates that at high concentrations the MID* mutant can transiently bind guide DNAs to cleave the RNA target in different registers. The MID* mutation also changes the pattern of the cleavage products observed with OH-gDNA (lanes 15-17), suggesting that the interactions of the MID pocket with an internal guide phosphate observed in the PliAgo structure (Fig. 2e) affect positioning of unphosphorylated guide DNAs. Thus, the 5’-end of guide DNA cannot be stably accommodated in the mutant MID* pocket, resulting in its sliding in the upstream direction from the active site. Overall, these results indicate that the MID pocket positions guide DNA in a defined register primarily through interactions with the 5’-phosphate group.

Additional protein contacts with the guide 5’-end observed in the structure with P-gDNA also contribute to its binding by PliAgo. In particular, alanine substitution of a lysine residue K759 that is involved in interactions with the 3^rd^ phosphate in guide DNA (Fig. 2b, c) and is located close to the last residue of the catalytic tetrad residue D762 significantly decreases the affinity of guide DNA in comparison with wild-type PliAgo (~20 fold, p-value of 0.004) (Supplementary Fig. 5a) but does not affect the cleavage site selection by PliAgo. Lysine at this position is conserved in the D-R group of pAgos (Supplementary Fig. 1b) and is replaced with histidine in previously studied pAgos (e.g. H657 in TtAgo).

### PliAgo does not use the C-terminus and Mg^2+^ ions for the guide 5′-end binding

Most known pAgos as well as eAgos from the PIWI branch use a Mg^2+^ ion bound in the MID pocket for interactions with the 5′-phosphate in guide DNA or RNA (Fig. 2d)^17,32,33,40,43,44,46^. The (Y/H/R)KQK motif in these Ago proteins forms a positively charged MID pocket suitable for Mg^2+^ coordination and 5′-phosphate interaction (residues R418/K422/K457 in TtAgo, Fig. 3b). In contrast, no Mg^2+^ ion is observed in the MID domain in the PliAgo/P-gDNA complex obtained even in the presence of 50 mM Mg^2+^ (Fig. 2c and 7R8J), suggesting that PliAgo does not use Mg^2+^ for coordination of the guide DNA 5’-phosphate. The MID pocket in PliAgo lacks functional groups from the (Y/H/R)KQK motif (containing neutral residues A479/C483/G542 at positions corresponding to charged residues in TtAgo) and the 5’-phosphate (because of its different positioning) that participate in the coordination of Mg^2+^ in other pAgos (compare Figs. 2c, d and 3b).

To decipher the role of divalent cations in guide DNA binding by the two groups of D-R pAgos, we compared the affinities of PliAgo and PnyAgo to guides in the presence and in the absence of Mg^2+^. We found that exclusion of Mg^2+^ from the reaction does not affect guide DNA binding by PliAgo but has a significant effect on PnyAgo (the affinity is decreased by more than an order of magnitude, *K*_d_ = 390 nM; *p*-value of 0.01) (Fig. 3d and Supplementary Fig. 5a). These results indicate that Mg^2+^ is critical for guide binding by PnyAgo, which has a standard MID pocket, while the MID pocket of PliAgo binds guide DNA without Mg^2+^.

The carboxy terminus of the protein is involved in interactions with the guide 5’-end in both pAgos and eAgos^3,33,42^. In particular, the C-terminal carboxyl group participates in coordination of the Mg^2+^ ion in the MID pocket in pAgos and PIWI eAgos (Fig. 2d)^14,17,32,33,40,43,44,47^. In contrast, the C-terminal 18–20 residues of PliAgo are disordered in all the structures indicating that the C-terminus is located away from the 5’-end binding pocket and does not interact with guide DNA (Figs. 2c, e and 3f).

To confirm that the C-terminus of PliAgo is not essential for guide DNA binding, we prepared a PliAgo derivative lacking its C-terminal 18 residues (PliAgo ΔC18, Δ772–789). For comparison, we analysed binding of guide DNA by CbAgo (a mesophilic D-D pAgo distantly related to TtAgo) that uses its C-terminus carboxyl group for coordinating Mg^2+^ in the MID pocket^12,40^. We obtained a truncated variant of CbAgo without 12 C-terminal residues (CbAgo ΔC12, Δ737–748), based on structural alignment of CbAgo^40^ with the position of the deletion in PliAgo (Fig. 3f). As expected, truncated CbAgo binds guide DNA with a strongly decreased affinity in comparison with the full-length protein (45-fold increase in the *K*_d_ value, *p*-value < 0.05) (Fig. 3e and Supplementary Fig. 5a). In contrast, the ΔC18 deletion in PliAgo does not compromise guide DNA binding and even slightly increases its affinity (*K*_d_ of 8 nM). Furthermore, the PliAgo ΔC18 derivative retains the catalytic activity toward target RNA (Supplementary Fig. 5d). These results indicate that unlike other pAgo and eAgo proteins, the C-terminus of PliAgo is dispensable for the guide 5’-end binding. In contrast, deletion of the C-terminal segment in CbAgo may directly affect coordination of 5’-guide DNA in the MID pocket and/or disrupt the interface between the MID and PIWI domains normally occupied by the C-terminus (Fig. 3f).

Overall, these results allow us to conclude that PliAgo and its relatives have a novel type of the guide binding pocket, which does not directly interact with the 5’-nucleotide base and does not rely on the Mg^2+^ ion and the C-terminus of pAgo for interactions with the 5’-phosphate. This opens a way for modification of their C-ends, which is not possible for other Ago proteins that use the C-terminus for guide binding. To explore this possibility, we obtained a variant of the PliAgo protein fused with GFP at its C-terminus (PliAgo-GFP). PliAgo-GFP binds guide DNA with high affinity (Fig. 3e and Supplementary Fig. 5a) and is catalytically active (Supplementary Fig. 5d). PliAgo-GFP can also be visualized within the cells by fluorescence microscopy (Supplementary Fig. 5e). This allows a greater flexibility in introducing potentially any desirable modifications from both ends of pAgos from this group.

### RNA target selection by D-R pAgos occurs at the cleavage step and depends on the nature of residues at the cleavage site

**Fig. 4:**
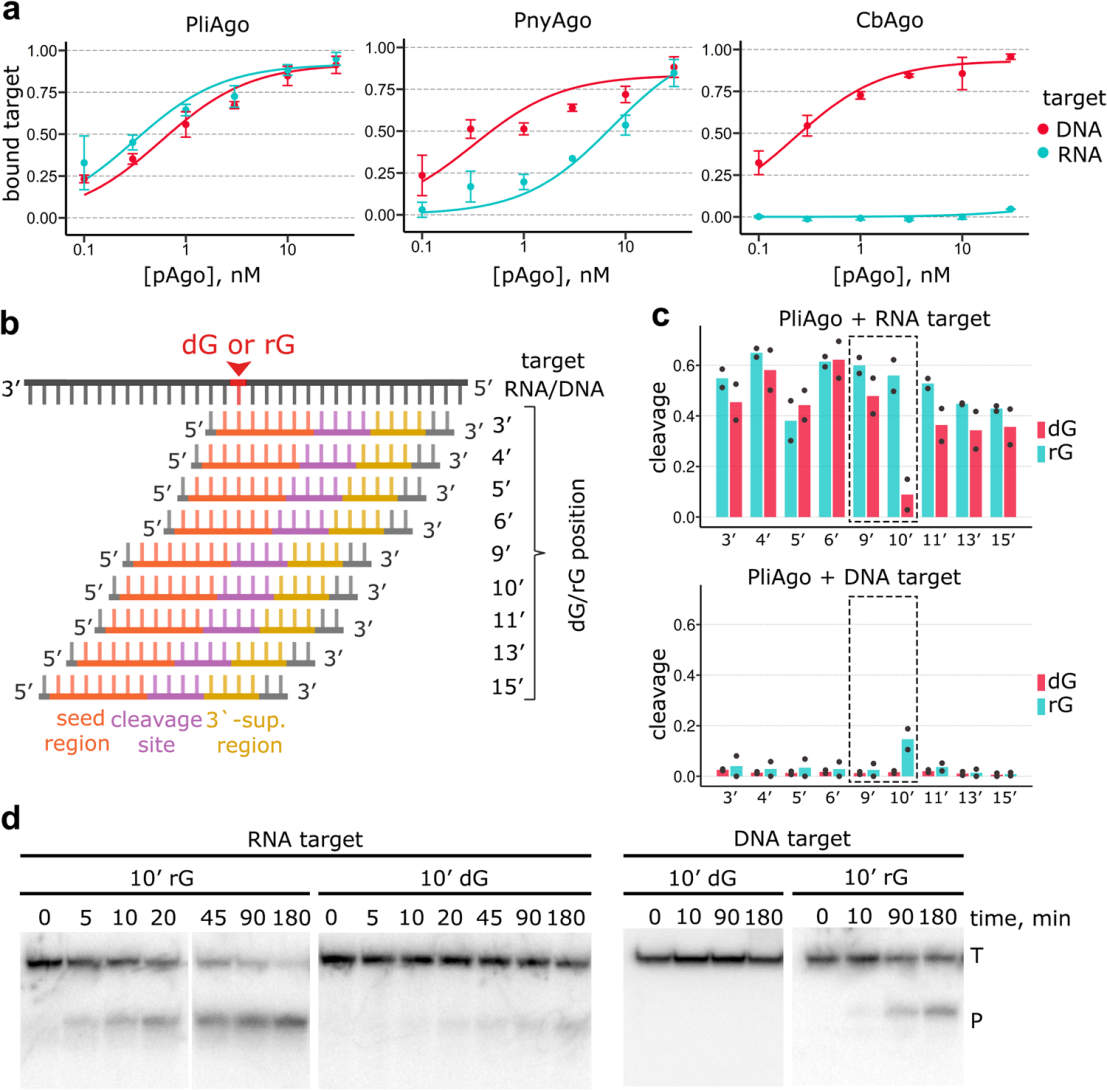
Specificity of target recognition by D-R pAgos. **a** Binding of target RNA and ssDNA of the same sequence by PliAgo, PnyAgo and CbAgo loaded with cognate guide DNA. Means and standard deviations from 3 independent experiments are shown. **b** Scheme of RNA/DNA targets containing a single dG/rG residue with differently positioned DNA guides (see Supplementary Table 1 for oligonucleotide sequences). Positions of the functional regions in guide DNA are indicated (seed, orange; central, magenta; 3’-supplementary region, ochre). **c** Effects of a single deoxyribonucleotide (dG) in the RNA target (top) and a single ribonucleotide (rG) in the DNA target (bottom) on target cleavage by PliAgo in comparison with control RNA and DNA targets. The reaction was performed at 37 °C for 30 min in the case of target RNA and for 3 h in the case of target DNA. Means from 2 independent experiments are shown. Target positions around the cleavage site are shown with dotted boxes. **d** Kinetics of target RNA (*left*) or target DNA (*right*) cleavage by PliAgo with containing ribonucleotide (rG) or deoxyribonucleotide (dG) residues at the position 10’. Representative gels from two independent experiments are shown.

To explore the molecular basis for the unusual preference of D-R pAgos for RNA targets, we analysed the interactions of PliAgo and PnyAgo with targets of various structures. We found that, when loaded with guide DNAs, both pAgos bind complementary target RNA with high affinity, with apparent *K*_d_s of 0.3 nM for PliAgo and 7.5 nM for PnyAgo (Fig. 4a, Supplementary Fig. 6a). Notably, catalytically inactive variants of both pAgos bind guide DNA and target RNA with the same affinities as wild-type proteins suggesting that they can potentially be used for programmable recognition of RNA targets without their cleavage (Fig. 4a, Supplementary Figs. 5a and 6a).

Surprisingly, single-stranded target DNA (ssDNA) is also efficiently bound by these pAgos, with the same affinity as RNA in the case of PliAgo or with an even higher affinity in the case of PnyAgo (Fig. 4a and Supplementary Fig. 6a). Equally efficient formation of ternary guide-target-pAgo complexes is also observed with RNA and ssDNA targets in an electrophoretic mobility shift assay performed with PliAgo (Supplementary Fig. 6b). Importantly, guide-loaded PliAgo does not bind corresponding double-stranded DNA, indicating that it cannot efficiently unwind double-stranded substrates for target sequence recognition (Supplementary Fig. 6c). In contrast to PliAgo, CbAgo, which strongly prefers DNA over RNA targets in the cleavage reaction^12^, has a dramatically lower affinity to RNA than to ssDNA (Fig. 4a). Thus, unlike CbAgo, the RNA-targeting pAgos can bind both single-stranded RNA and DNA molecules but discriminate between them at downstream processing steps.

To further understand discrimination between RNA and DNA targets, we performed cleavage reactions with an RNA target containing a single 2’-deoxyribonucleotide residue (dG or dT) at different positions relative to the site of cleavage, using a series of differently positioned DNA guides (Fig. 4b). For PliAgo, target RNA cleavage is dramatically decreased when the deoxyribonucleotide residue is placed in position 10′, but not in other positions (Fig. 4c, d; Supplementary Fig. 6e). For PnyAgo, the greatest effect on cleavage is observed for deoxyribonucleotide in position 11′ (Supplementary Fig. 6d, e). Similar site-specific effects of deoxyribonucleotide residues are observed for RslAgo (for position 10’) and HpeAgo (for position 11’) (Supplementary Fig. 6f). In comparison, 2′-fluoro modification in the target RNA, which can increase resistance to some RNases^48^ but supports hydrogen bonding^49^, has no effect on RNA cleavage by PliAgo independently of its position (Supplementary Fig. 7a, b).

Conversely, when a single ribonucleotide (rG or rU) is introduced in the DNA target, it significantly stimulates target cleavage by PliAgo when placed in position 10’ and by PnyAgo when placed in position 11’ (and, to some extent, in position 10’), in comparison with the control DNA target (Fig. 4c, d and Supplementary Fig. 6d). In contrast, for CbAgo neither a single deoxyribonucleotide in the RNA target can stimulate cleavage nor a single ribonucleotide at any position of the DNA target can inhibit its cleavage (Supplementary Fig. 6d). Thus, for both PliAgo and PnyAgo, the critical role in discrimination between RNA and DNA targets is played by the nature of the residue upstream of the cleavage site (located between positions 9’ and 10’ for PliAgo and positions 10’ and 11’ for PnyAgo). Overall, these results indicate that RNA target selection by the D-R pAgos is mainly achieved at the cleavage step of the reaction, and not during initial target binding.

To get further insight into the mechanism of target RNA cleavage by PliAgo, we analyzed alanine substitution of a conserved lysine residue K565, which is specific to the PliAgo group of D-R pAgos and is replaced with histidine in PnyAgo or with a small residue in previously studied pAgos (Supplementary Fig. 7c). K565 is adjacent to the catalytic tetrad residue D563 and may likely contact target RNA in the active site (Fig. 2b). Substitution K565A significantly decreases the apparent affinity of target RNA in comparison with wild-type protein (~3.4-fold, p-value 0.005) (Supplementary Fig. 6a) and also decreases the rate of the reaction at saturating pAgo concentrations (~3.3-fold, t_1/2_ of ~115 min in comparison with 34.6 min for wild-type PliAgo). At the same time, the K565A substitution does not change the preference of PliAgo for target RNA over DNA, and RNA cleavage by the mutant is similarly inhibited by the 2’-deoxynucleotide residue upstream of the site of cleavage (Supplementary Fig. 7d, e). In comparison, an adjacent substitution N566A, which does not face target RNA, has no significant effect on target RNA binding and cleavage (Supplementary Fig. 6a and Supplementary Fig. 7d, e). Therefore, contacts of positively charged residues at the active site of PliAgo (K565 and possibly R473 and K663) with target RNA are likely important for its proper positioning and cleavage.

To confirm that the analyzed mutations in PliAgo do not strongly affect protein folding, we measured thermal denaturation curves for all protein variants using a label-free assay based on changes in the intrinsic protein fluorescence upon heating (see Methods). The thermal unfolding curves of PliAgo and its mutants were highly similar. The midpoint temperatures of thermal denaturation (inflection temperatures, Ti), were identical for all proteins (50.4 °C for wild-type PliAgo, 52.0 °C for MID*, 52.9 °C for K759A, 51.3 °C for K565A, 51.7 °C for N566A and 51.1 °C for ΔC18 PliAgo variants). For the PliAgo-GFP fusion, two thermal transitions were detected (Ti_1_ 52.1 °C, Ti_2_ 89.4 °C), corresponding to denaturation of PliAgo and GFP, respectively. The results indicate that the mutations in PliAgo do not affect protein stability.

### D-R pAgos sense modifications and single-nucleotide mismatches in RNA targets

To characterize the specificity of target RNA recognition and unveil the potential of D-R pAgos for RNA technologies, we analysed their ability to recognize RNA targets containing other types of site-specific modifications or mismatches with the guide sequence.

**Fig. 5:**
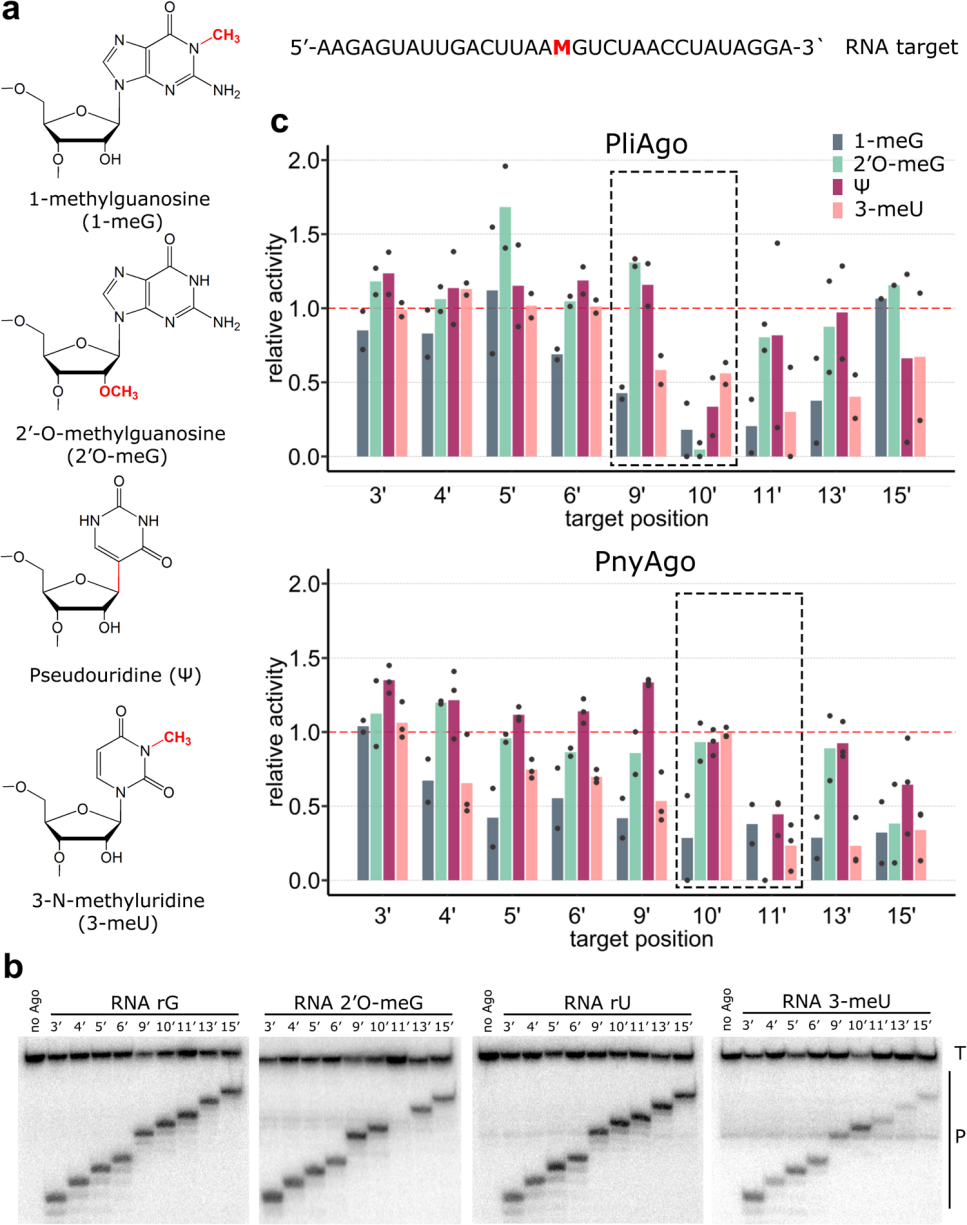
D-R pAgos sense RNA modifications at specific target positions. **a** Modified nucleotide residues with detected effects on the target RNA cleavage. The target RNA sequence is shown on the right (M, modified nucleotide), the guides are shown in Supplementary Fig. 8a. **b** Cleavage of modified RNAs containing 2’O-meG or 3-meU at the indicated positions by PnyAgo in comparison with control RNAs (rG or rU). Representative gels from 2 or 3 independent experiments. **c** Effects of RNA modifications at different positions relative to the guide 5’-end on target RNA cleavage by PliAgo (top) and PnyAgo (bottom). Target positions around the cleavage site are shown with dotted boxes. For each position, the efficiency of cleavage of modified RNA was normalized to the efficiency of cleavage of control RNA measured with the same guide DNA (‘relative activity’). Means from 2-3 independent experiments are shown.

To test whether D-R pAgos can detect RNA modifications, we analyzed cleavage of a series of RNA targets containing various modified nucleotides, including 2’-O-methylguanosine (2’O-meG), 1-methylguanosine (1-meG), pseudouridine (Ψ), 3-methyluridine (3-meU), N^6^-methyladenine (N^6^-meA), inosine (I) and 5-methylcytosine (5-meC) (Fig. 5a and Supplementary Fig. 8). For each RNA target, we used several differently positioned DNA guides so that the modified target nucleotide was placed at different positions relative to the site of cleavage (Supplementary Fig. 8a). We observed that pAgos sense the presence of 2’O-meG, 1-meG, Ψ and 3-meU (Fig. 5b, c, Supplementary Fig. 8b), while the other three modifications only weakly affect target RNA cleavage (Supplementary Fig. 8c). In particular, 2’O-meG is sensed at position 10’ by PliAgo, positions 9’ and 10’ by RslAgo, and at position 11’ by PnyAgo and HpeAgo (light green in Fig. 5c and Supplementary Fig. 8b). This is similar to the effects of 2’-deoxynucleotides in target RNA (see the previous section) and further confirms that the presence of an intact 2’-OH group upstream of the cleavage site is crucial for the reaction. Modificatons of nucleobases can also affect cleavage in a position-specific way. 1-meG strongly decreases the cleavage efficiency at positions 10’ and 11’ for PliAgo, positions 5’, 6’, 9’, 10’ and 11’ for RslAgo, and at most positions (except for 9’) for HpeAgo, but has only moderate effects on RNA cleavage by PnyAgo (dark green bars in Fig. 5c and Supplementary Fig. 8b). Ψ has no strong effects on RNA cleavage at most positions but decreases the cleavage efficiency about 2-fold when present at position 10’ for PliAgo and RslAgo, and at position 11’ for PnyAgo and HpeAgo (dark red in Fig. 5c and Supplementary Fig. 8b). Finally, 3-meU has moderate effects on RNA cleavage by PliAgo and PnyAgo (2-3 fold decrease for positions 9’−15’), but strongly decreases cleavage at positions 5’, 6’, 9’, 10’, 11’ by RslAgo and at most positions (except for positions 9’ and 10’) by HpeAgo (light red in Fig. 5c and Supplementary Fig. 8b).

These results demonstrate that D-R pAgos can sense the presence of various types of modifications at specific positions of RNA targets. Notably, in most cases the strongest effects on cleavage are observed when the modified nucleotide is present at position 11’ for the first group of pAgos (PliAgo and RslAgo) and at position 10’ for the second group (PnyAgo and HpeAgo). This corresponds to the first nucleotide upstream of the site of target cleavage and is similar to the effects of single deoxynucleotides/ribonucleotides described above.

**Fig. 6:**
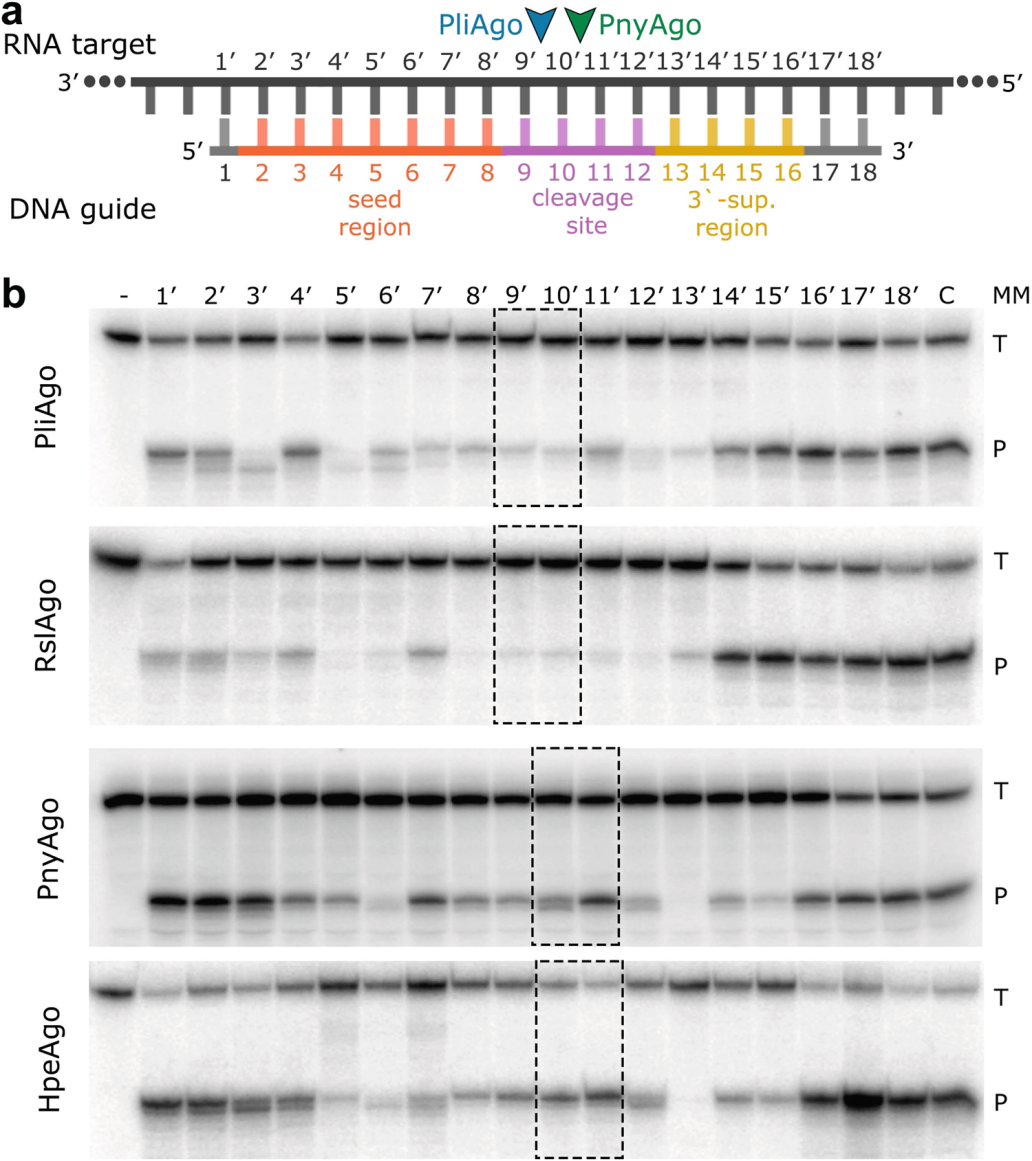
D-R pAgos sense mismatches in the RNA target. **a** Scheme of the guide-target duplex with indicated functional regions in guide DNA. The cleavage sites by PliAgo and PnyAgo are indicated. **b** Effects of single-nucleotide mismatches on the efficiency of RNA cleavage by D-R pAgos (see Supplementary Table 1 for guide DNA and target RNA sequences). The reactions were performed with 5’-P^32^-labeled target RNA at 37 °C for 60 min for PliAgo, RslAgo, HpeAgo and 10 min for PnyAgo; positions of the target (‘T’) and the reactions products (‘P’) are indicated. Representative gels from three independent experiments are shown (see Supplementary Fig. 9 for means and errors). The positions around the cleavage sites of corresponding pAgo proteins are shown with dotted boxes.

Finally, we tested the effects of mismatches on target RNA cleavage using a series of guides based on the same sequence with single-nucleotide substitutions at different positions (Fig. 6a). It was shown that for PliAgo and RslAgo the efficiency of cleavage is significantly decreased (more than 2-fold) for mismatches at most positions, except for position 1’ (and also 2’ and 4’ for PliAgo) and positions starting from position 14’ (Fig. 6b and Supplementary Fig. 9). In addition, the cleavage site is shifted with some mismatches (at positions 2’, 3’, 5’, 6’ and 7’), indicating that the mismatch disturbs the geometry of the catalytic complex. For PnyAgo, target RNA cleavage is decreased for most mismatches between positions 3’ and 15’ and is eliminated for a mismatch at position 13’. For HpeAgo, cleavage is decreased for mismatches at positions 5’−9’, 12’, 14’, 15’ and is also eliminated for a mismatch at position 13’ (Fig. 6b and Supplementary Fig. 9). Notably, RNA cleavage by PliAgo and RslAgo is more strongly affected by changes at the site of cleavage in comparison with PnyAgo and HpeAgo. All four pAgos are highly sensitive to the mismatch at position 13’ indicating that this position is critical for target cleavage. Overall, the results suggest that pAgos from the D-R group can be used for highly specific RNA target recognition and cleavage.

## Discussion

Programmable nucleases guided by small nucleic acids use a straightforward mechanism based on complementary interactions to identify and destroy specific target sequences, thus making them versatile tools in genetic engineering and biotechnology^1,11,50,51^. Enzymes studied to date have specific preferences in respect to the nature of both guide and target molecules. Hypothetically, both RNA and single-stranded DNA molecules can be used as guides to recognize RNA or DNA targets, corresponding to four possible types of programmable nucleases^2^, three of which have been identified to date. In particular, eukaryotic Ago proteins are loaded with several types of small RNAs to suppress the expression of mRNA targets (R-R nucleases)^7^. Prokaryotic CRISPR-Cas systems use RNA guides to recognize and cleave DNA or RNA targets (R-D or R-R nucleases)^4–6^. Previously studied pAgo proteins use DNA guides or, more rarely, RNA guides to recognize DNA targets (D-D or R-D nucleases)^3^. While some pAgos, including TtAgo, MpAgo from *Marinitoga piezophila* and KmAgo from *Kurthia massiliensis*, were reported to cleave RNA targets in vitro, they are all more active with DNA targets and likely recognize DNA in vivo^14,16,19–21,25,37^. Here, we have characterized the remaining type of D-R nucleases that exclusively bind small DNA guides to recognize and cleave RNA targets. The D-R pAgos do not bind RNA guides and cannot cleave DNA targets efficiently, suggesting that RNA is their primary target in vivo.

We have discovered two related groups of D-R pAgos, which are significantly different in the mode of their interactions with guide DNA and in the structure of the MID pocket involved in these interactions (Supplementary Movie 1). pAgos from the PnyAgo group have an HKQK motif in the MID pocket responsible for the guide 5’-end binding, which is similar to the motif found in other pAgos (Fig. 3a). In the binary complexes of these pAgos, the 5’-nucleotide of guide DNA or RNA is unstacked from the second nucleotide and is placed into the MID pocket (Fig. 2d)^17,32,42–44^. Accordingly, these pAgos cleave target RNA at the standard position between the 10^th^ and 11^th^ guide nucleotide (Fig. 1d). In contrast, pAgos from the PliAgo group have a novel type of the MID pocket with an RHSY motif not found previously in any other pAgo or eAgo protein (Fig. 3a, Supplementary Fig. 1c). The MID pocket of PliAgo does not bind a divalent cation and PliAgo does not use the C-terminal carboxyl group for interactions with guide DNA. The RHSY motif interacts with the 5’-phosphate of guide DNA in such a way that the first nucleotide of the guide maintains stacking interactions with the next nucleotide and the whole seed segment of guide DNA adopts an A-helical conformation (Fig. 2). This shifts the register of guide DNA by one nucleotide in comparison with other Agos and results in a corresponding shift in the site of target RNA cleavage. This structure of the MID pocket is conserved in the PliAgo group of pAgos (Fig. 3a, Supplementary Fig. 1b), suggesting that they all utilize a similar mechanism of guide DNA binding and explaining their unconventional cleavage site preferences.

Another group of pAgo proteins that use non phosphorylated RNA guides, including MpAgo from *M. piezophila*, was also previously shown to have an unusual MID pocket, lacking bound divalent cations and containing substitutions of polar residues interacting with the guide 5’-phosphate in other Agos (Fig. 3a)^21^. However, unlike pAgos from PliAgo group, the MID pocket of MpAgo is still related to the majority of pAgos based on the sequence alignment (Fig. 3a). Accordingly, the overall geometry of bound guide RNA and target DNA and the target cleavage site in MpAgo remain the same as in other pAgos^21,34^.

By analyzing the structure of a binary complex of PliAgo with non phosphorylated guide DNA, we have also revealed the molecular basis for the observed preference of PliAgo and other D-R pAgos for 5’-P guide DNAs. In the complex with non-phosphorylated guide DNA, the guide 5’-end slides from the MID pocket by one nucleotide in the 5' direction, and is fixed in the new position by interactions with an internal phosphate group of guide DNA. These changes decrease the affinity of guide DNA to PliAgo and also affect the precision of the cleavage reaction. The results demonstrate that the presence of the 5’-phosphate in guide DNA is important for positioning of the guide and the target within PliAgo during cleavage.

We have shown that target RNA cleavage by D-R pAgos is sensitive to mismatches at different positions of the guide-target duplex, including the seed, central and 3’-supplementary regions of guide DNA (Fig. 6). Furthermore, D-R pAgos sense site-specific modifications in the RNA target, including nonbulky adducts of nucleobases and the ribose moiety, with the strongest effects observed for nucleotide modifications around the site of cleavage and upstream of it (Fig. 5). This suggests that the cleavage reaction is highly sensitive to distortions in the conformation of the RNA-DNA hybrid or changes in the RNA-protein contacts affecting substrate positioning in the active site of D-R pAgos.

Highly specific RNA targeting by D-R pAgos paves the way for their applications in RNA biology and biotechnology. Previously, DNA-targeting pAgos have been applied for specific cleavage or detection of target DNA sequences in vitro^12,40,52,53^, but their potential for in vivo applications has remained in question^11,54^. In comparison, D-R pAgos can potentially be used for specific RNA cleavage and modification, or for identification of single-nucleotide polymorphisms, non-canonical nucleotides and other types of modifications in mRNA and noncoding RNA targets^55^. Eukaryotic RNAi and eAgo proteins have long served as an efficient instrument for gene silencing based on targeting of specific mRNA targets with small interefering RNAs. The newly discovered D-R pAgos may serve as an orthogonal and more specific tool for RNA silencing and modification, acting independently of eAgos and other components of the host RNAi system. In comparison with some previously characterized Ago proteins^16,17,27,33,42^, the D-R pAgos do not require the presence of particular motifs in the guide 5’-end and can potentially be programmed with DNA guides of any sequence to recognize desired RNA targets. Importantly, D-R pAgos cannot cleave DNA targets and cannot recognize double-stranded DNA, likely preventing their undesired reactions with DNA targets in vivo. The physiological temperature range of activities of D-R pAgos makes them potentially suitable for in vivo experiments in both mesophilic prokaryotes and eukaryotes. Furthermore, the ability to modify both protein termini in PliAgo and related pAgos without the loss of their activity provides extended opportunities for their modification and creating fusions with signaling or effector domains for in vivo applications.

The unusual specificity of the D-R pAgos raises questions about their natural biological functions. Recent studies suggested that DNA-targeting pAgo proteins are active components of prokaryotic defense systems and may be involved in other genetic processes including decatenation of chromosomal DNA during replication^3,26,37^. D-R pAgos may also participate in the antiphage defense or in the regulation of gene expression. As shown for Type VI CRISPR-Cas systems (Cas13 nucleases), targeting of mRNA transcripts may be an efficient strategy to suppress phage infection^56^. However, in contrast to the Cas nucleases that are guided by crRNAs encoded in the CRISPR cassettes, D-R pAgos must be first loaded by guide DNAs derived from the target genetic element. It can therefore be speculated that D-R pAgos may cooperate with other nucleases to disrupt both the genome and the transcriptome of an invading genetic element. The association of all D-R pAgos with a conserved nuclease (Fig. 1b) suggests that it might be involved in the processing of small guide DNAs used by pAgos or participate in the degradation of target genetic elements together with pAgos. Analysis of the potential roles of D-R pAgos and associated nucleases in the suppression of foreign genetic elements and in gene regulation will be important directions for further studies.

### Note added in proof

While this study was under review, another pAgo from the PnyAgo group (MpaAgo, Fig. S1) was shown to be a DNA guided RNA nuclease^57^.

## Methods

### Expression and purification of pAgos

The genes encoding pAgos from *R. slithyformis* (WP_013921749.1), *P. nyackensis* (WP_084286803.1) and *H. penzbergensis* (WP_139173808.1) were obtained by PCR from the genomic DNA purified from native bacterial strains. The DNA sequence of PliAgo (WP_100161590.1 *P. lipolyticus*) was codon-optimized using the IDT Codon Optimization Tool for expression in *E. coli* and synthesized as gBlocks Gene Fragments. All four genes were cloned into the pET-28b vector with an amino-terminal His_6_ sequence. Mutant variants of pAgos were obtained by PCR-mutagenesis and cloned in the same way, followed by plasmid sequencing. To obtain the fusion protein PliAgo-GFP, the EGFP gene was fused to the PliAgo gene via two Gly-Gly-Gly-Ser repeat linkers.

For protein expression, the strain *E. coli* BL21(DE3) was transformed with plasmids encoding pAgos, the cells were grown in the Luria-Bertrani (LB) broth supplemented with 50 μg/mL kanamycin at 30 °C with aeration until OD_600_ 0.3–0.4. The cultures were cooled down to 16 °C, induced with 0.1 mM IPTG, grown for 16 h at 16 °C and harvested by centrifugation. In the case of PliAgo and RslAgo, the cells were resuspended in buffer A (50 mM Tris-HCl pH 7.4, 500 mM NaCl, 20 mM imidazole, 5% glycerol, 1 mM PMSF) and disrupted using Cell Disruptor CF (Constant Systems) at 30 kpsi. The lysate was centrifuged at 35,000 g for 30 min and the supernatant was loaded onto a HisTrap Fast Flow crude column (GE Healthcare) packed with Ni-Sepharose and equilibrated with the same buffer. The column was washed with buffer A containing 60 mM imidazole and the protein was eluted with buffer A containing 250 mM imidazole. The elution fractions were concentrated using an Amicon Ultra centrifugal filter unit 50 K (Millipore) and loaded onto a Superose 6 10/300 GL column (GE Healthcare) equilibrated with buffer GF (10 mM HEPES–NaOH pH 7.0, 500 mM NaCl, 5% glycerol, 1 mM DTT). The pAgo-containing fractions were loaded onto a Heparin Fast Flow column (GE Healthcare) equilibrated with buffer GF, washed with 10 column volumes of GF and eluted with the same buffer containing 700 mM or 1 M NaCl. The mutant variants of PliAgo and CbAgo were purified with the same protocol using HisTrap Fast Flow crude and Heparin Sepharose columns. PnyAgo, its inactive derivative, and HpeAgo were purified in a similar way using a HisTrap Fast Flow crude column equilibrated in 10 mM Tris–HCl pH 7.9, 500 mM NaCl, 10 mM imidazole, 5% glycerol, 1 mM PMSF. The column was washed with the same buffer containing 30 mM imidazole and the proteins were eluted with a linear imidazole gradient. The protein-containing fractions were diluted to a 100 mM NaCl concentration and loaded onto a HiTrap SP HP column equilibrated with 40 mM Tris–HCl pH 7.9, 0.1 mM DTT, 0.5 mM EDTA, 5% glycerol. Proteins were eluted with buffer containing 500 mM NaCl. In all cases, the samples were concentrated by ultrafiltration (Amicon-50K), aliquoted with 50% glycerol and stored at −20 °C. The purity of the samples was analyzed by SDS-PAGE, and the protein concentration was determined by NanoDrop-1000 Spectrophotometer or Qubit Fluorometer (Thermo Fisher Scientific).

For expression of PliAgo-GFP, the culture of *E. coli* BL21 (DE3) containing the plasmid pET28-PliAgo-GFP was grown at 30 °C until OD_600_ 0.3, cooled down to 16 °C and induced with 0.1 mM IPTG for 18 h at 16 °C. 10 microliters of the culture were mixed with DAPI (4’,6-diamidino-2-phenylindole dihydrochloride) (Thermo Fisher Scientific) and incubated in the dark for 20 min at 22 °C. The cells were put on a slide, air-dried and heat-fixed using a spirit lamp. Then the SlowFade Gold Antifade Mountant reagent (Thermo Fisher Scientific) was added and the sample was covered with a cover glass. Fluorescence microscopy was performed with a 63 × oil immersion objective on a ZEISS Axio Observer microscope (Carl Zeiss, Jena, Germany) equipped with appropriate filter sets to detect fluorescence of GFP and DAPI. Images were acquired and analyzed using the ZEN microscope software.

For structure determination, PliAgo was expressed in the same way as described above. The cells were lysed in 40 mM Tris-HCl, pH 8.0, 500 mM NaCl, 5% glycerol, 0.1 mM DTT and 1 mM PMSF by sonication. The lysate was applied to a Ni-Sepharose column equilibrated with 50 mM Tris-HCl, pH 7.5, 500 mM NaCl, 5% glycerol and 10 mM imidazole followed by eluting the protein with the same buffer plus 250 mM imidazole. The eluate was further purified on a Superdex-200 size-exclusion column equilibrated with 10 mM HEPES, pH 7.0, 400 mM NaCl, 5% glycerol and 1 mM DTT. The fractions containing PliAgo were combined and applied to a 5 ml Heparin column equilibrated with 10 mM HEPES, pH 7.0, 400 mM NaCl, 5% glycerol and 1 mM DTT. The protein was eluted with a salt gradient from 400 mM to 1000 mM NaCl. The fractions containing PliAgo were combined, concentrated to 26 mg/ml and stored at −80 °C. For preparing SeMet labeled PliAgo, the BL21(DE3) cells expressing PliAgo were grown in SeMet growth media^58^ containing 50 μg/ml kanamycin. The SeMet-labeled PliAgo was purified in the same way as described above for native PliAgo.

### Protein thermal stability measurements

Measurements of thermal stability of PliAgo and its mutant variants were performed using a Tycho NT.6 instrument (NanoTemper Technologies). The experiments were carried out in 10 mM Hepes buffer with 350 mM NaCl, 50% glycerol, pH 7.0. Protein samples (10 μM) were loaded into Tycho NT1.6 capillaries and heated at 30 °C/min. Protein unfolding was recorded by measuring changes in tryptophan and tyrosine fluorescence at emission wavelengths of 330 and 350 nm as a function of increasing temperature. The thermal inactivation values (Ti, inflection temperature) were determined from peaks of the first derivative of the fluorescence changes using internal evaluation features of the Tycho instrument.

### Analysis of nucleic acid cleavage by pAgos

The sequences of all oligonucleotides used in the assays are shown in Supplementary Table 1. Modified RNA oligonucleotides were obtained from Horizon Discovery Biosciences Limited. Cleavage assays were performed in reaction buffer containing 10 mM Tris-HCl pH 7.9, 100 mM NaCl, 5 mM MgCl_2_, 5% glycerol. When required, oligonucleotides were 5′*-*phosphorylated with ATP or radiolabeled with γ-P^32^-ATP using T4 polynucleotide kinase (New England Biolabs). Binary pAgo-guide complexes were assembled by mixing pAgo and guide oligonucleotides (500 nM and 200 nM, respectively, unless otherwise indicated) at 37 °C for 15 min. The cleavage reactions were initiated by the addition of 100 nM target RNA or DNA at 37 °C and the samples were incubated for indicated time intervals. To analyze the effect of various divalent cations, 0.5 mM or 5 mM MnCl_2_, CaCl_2_, CoCl_2_, CuCl_2_ or ZnCl_2_ were added to the reaction buffer instead of MgCl_2_. Analysis of temperature dependence of target cleavage was carried out in a PCR thermocycler (T100, Bio-Rad) at different temperatures. Kinetic analysis of target RNA cleavage was performed using 5’-radiolabeled RNA targets in single-turnover reaction conditions. In some reactions with mutant variants of pAgos, the efficiency of target cleavage was measured with lower concentrations of the reaction components, 50 nM pAgo, 25 nM guide DNA, and 25 nM target RNA for indicated time intervals. To determine the effect of mismatches on the target cleavage, a set of DNA guides was used, each containing a single mismatched nucleotide at a certain position, the reaction was performed for 1 h (10 min in the case of PnyAgo) at 37 °C. Analysis of the effects of RNA modifications was performed using RNA targets containing modified nucleotides at the 17th position from the 5’-end and a set of 18 nt (for PnyAgo and HpeAgo) or 16 nt guides (for PliAgo and RslAgo) with different positions relative to the site of modification (Supplementary Table 1) for indicated time intervals at 37 °C.

In all cases, the reactions were stopped after indicated time intervals by mixing the samples with equal volumes of stop solution (8 M urea, 20 mM EDTA, 0.005% Bromophenol Blue). The cleavage products were resolved by 19% and 23% PAGE, pre-stained with SYBR Gold (Invitrogen) for unlabeled oligonucleotides or visualized with a Typhoon FLA 9500 scanner (GE Healthcare). The data were analyzed using ImageQuant (GE Healthcare) and custom R scripts (version 4.0.0). The data of single-turnover reactions were fitted to the equation: Y = C + A_max_ × (1 ‒ exp(–*k*_obs_ × *t*)), where Y is the efficiency of cleavage at a given time point, A_max_ is the maximum cleavage, C – the background level of cleavage, and *k*_obs_ is the observed rate constant.

### Analysis of guide and target binding by pAgos

Determination of apparent dissociation constants (*K*_d_) for complexes of pAgos with guides and targets was performed by quantification of their binding to nitrocellulose filters using a dot blot assay. For analysis of guide binding, 5’-P^32^-labeled DNA or RNA guide oligonucleotides (0.1 nM final concentration) were mixed with increasing concentrations of pAgo in the reaction buffer (containing or lacking Mg^2+^ ions) with the addition of 100 μg/ml BSA. For analysis of target binding, 5’-P^32^-labeled DNA or RNA target oligonucleotides were mixed with preformed binary guide-pAgo complexes (1:1 ratio of guide DNA and pAgo). The mixtures were incubated at 37 °C for 20 min (for analysis of guide binding) or for 5 min (for analysis of target binding) and loaded onto a Bio-Dot Microfiltration Apparatus (Bio-Rad) loaded with nitrocellulose membrane (Merck–Millipore) on top of nylon Hybond N + membrane (GE Healthcare) pre-washed with the reaction buffer. The samples were filtered through the membranes, the membranes were washed three times with the reaction buffer, dried at 63 °C for 20 min and visualized with a Typhoon FLA 9500 scanner (GE Healthcare). The fraction of bound guide or target oligonucleotides was calculated as the ratio of the amounts of labeled DNA or RNA bound to the nitrocellulose membrane to the sum of the signals from the nitrocellulose and Hybond N + membranes. The data were analyzed using ImageQuant (GE Healthcare) and custom R scripts (version 4.0.0). The data were fitted to the equation: B = B_max_ × C/(*K*_d_ + C), where B is the fraction of bound substrate, A_max_ is the maximum binding, and C is the concentration of pAgo or of the binary guide-pAgo complex. The affinity of 5’-OH guides was measured in a competition assay with 5’-P^32^-labeled guide DNA. Unlabeled 5’-P or 5’-OH guide DNA was mixed at increasing concentrations with 0.1 nM labeled guide DNA in the reaction buffer containing 100 μg/ml BSA followed by the addition of 100 nM pAgo. The mixture was incubated for 30 min at 37 °C and then filtered through nitrocellulose membrane as described above. The data were fitted as described in ref. 59.

For analysis of target RNA and DNA binding by the electrophoretic mobility shift assay (EMSA), the ternary pAgo-guide-target complexes were assembled with wild-type PliAgo or catalytically inactive dPliAgo. Target dsDNA was obtained by pre-annealing of 5’-P^32^-labeled target ssDNA  with a complementary nontarget strand and purified from 10% native PAGE. The reaction mixtures containing 5’-P guide DNA (102.4 nM G-guide) with pAgos (204.8 nM) were incubated in the reaction buffer containing 100 μg/ml BSA for 15 min at 37 °C. The 5’-[P^32^]-labeled target ssRNA, ssDNA or dsDNA (0.1 nM) was then mixed with increasing concentrations of the binary complex (0, 0.2, 0.4, 0.8, 1.6, 3.2, 6.4, 12.8, 25.6, 51.2 nM) and incubated for 5 min at 37 °C. The samples were mixed with 5× loading buffer (5× TBE, 50% glycerol, 0.005% Bromophenol Blue), resolved by electrophoresis in native 4–20% Mini-PROTEAN TGX gels (Bio-Rad) run at constant voltage (120 V) for 80 min in ice-cold bath, and visualized by phosphorimaging (Typhoon FLA 9500).

### Crystallization of PliAgo

All crystals were grown by the hanging drop vapor diffusion method at room temperature. Native wild-type PliAgo and SeMet-labeled PliAgo crystals were grown by mixing 1 μl of 20 mg/ml PliAgo solution with 1 μl of reservoir solution containing 100 mM Tris-HCl (pH 8.0) and 8% PEG 8000. For crystallizing of the PliAgo and gDNA complexes PliAgo was mixed with DNA at 1:1.5 molar ratio before mixing with the crystallization solution. The guide DNAs containing or lacking the 5’- phosphate were purchased from IDT (5′-TTACTGCACAGGTGACGA-3′). The crystals were transferred to cryoprotection solutions containing 100 mM Tris-HCl (pH 8.0), 15% butanediol plus 15% PEG 8,000 and flash-frozen with liquid nitrogen. For crystallizing the PliAgo and gDNA complex in the presence of Mg^2+^, the complex was mixed with the crystallization solution containing 50 mM MgCl_2_ and the crystals were transferred to the cryo-solution containing 50 mM MgCl_2_ and flash-frozen with liquid nitrogen.

### X-ray data collection and processing

The X-ray data were collected at the Macromolecular Diffraction facility at the Cornell High Energy Synchrotron Source (MacCHESS) 7B2 beamline (Cornell University, Ithaca, NY) and processed by HKL2000 (version 718.05)^60^. All crystals (apo-form and DNA bound forms) belonged to the I23 space group and contained one molecule in an asymmetric unit.

### Structure determination and refinement

With the anomalous signal from SeMet, 19 selenium sites in the asymmetric unit were located and the experimental phase (figure of merit: 0.390) was calculated using Automated structure solution (AutoSol) in PHENIX (version 1.20rc3-4406)^61^. Density modification using native crystal data by AutoSol yielded an excellent map and the structure of TtAgo (PDB:3DLB) was used as a guide for manual model building by Coot (version 0.9.3)^62^ followed by the structure refinement using PHENIX. The resolution limits for crystallographic datasets were determined based on the completeness (>80%) and CC1/2 (>20%) values rather than R_merge_ and < I > /sigmaI > 2 criteria, since this approach prevents loss of useful crystallographic data for structure refinement^63^. The structures of PliAgo and DNA complexes were determined by the molecular replacement (PHENIX) using apo-form PliAgo structure followed by DNA model building and structure refinement by PHENIX. Final coordinates and structure factors have been deposited to the Protein Data Bank (PDB) with the accession codes listed in Supplementary Table 2.

### Reporting summary

Further information on research design is available in the Nature Research Reporting Summary linked to this article.

## Supplementary information

Supplementary Information

Peer Review File

Description of Additional Supplementary Files

Supplementary Movie 1

Reporting Summary

## Data Availability

The data that support this study are available from the corresponding authors upon request. The coordinates are deposited in the Protein Data Bank with PDB accession codes 7R8F (native PliAgo), 7R8G (PliAgo-OH-gDNA complex), 7R8H (PliAgo-P-gDNA complex), 7R8J (PliAgo-P-gDNA-Mg2+ complex) and 7R8K (SeMet PliAgo). Source data are provided with this paper.

## Source data

Source Data
