# Author Correction: Programmable RNA targeting by bacterial Argonaute nucleases with unconventional guide binding and cleavage specificity

**DOI:** 10.1038/s41467-022-32616-2

**Published:** 2022-08-17

**Authors:** Lidiya Lisitskaya, Yeonoh Shin, Aleksei Agapov, Anna Olina, Ekaterina Kropocheva, Sergei Ryazansky, Alexei A. Aravin, Daria Esyunina, Katsuhiko S. Murakami, Andrey Kulbachinskiy

**Affiliations:** 1 Institute of Molecular Genetics, National Research Center “Kurchatov Institute”, Moscow, Russia; 2 Institute of Gene Biology, Russian Academy of Sciences, Moscow, Russia; 3 Department of Biochemistry and Molecular Biology, Pennsylvania State University, University Park, PA USA; 4 Division of Biology and Biological Engineering, California Institute of Technology, Pasadena, CA USA; 5 Present Address: Department of Biochemistry and Molecular Biophysics, Columbia University, New York, NY USA

**Keywords:** RNAi, CRISPR-Cas systems, X-ray crystallography

**Correction to**: *Nature Communications* 10.1038/s41467-022-32079-5, published online 08 August 2022

In this article the affiliation ‘Institute of Gene Biology, Russian Academy of Sciences, Moscow 119334, Russia’ for Andrey Kulbachinskiy was incorrectly assigned as a present address. The original article has been corrected.

