# Supplementary material for: Programmable RNA targeting by bacterial Argonaute nucleases with unconventional guide binding and cleavage specificity
Source: Nat Commun. 2022 Aug 8;13:4624. doi: 10.1038/s41467-022-32079-5 (PMC9360449; doi:10.1038/s41467-022-32079-5)
Supplement: Supplementary file 1 — Supplementary Information [file 41467_2022_32079_MOESM1_ESM.pdf]

Programmable RNA targeting by bacterial Argonaute nucleases with unconventional guide binding and cleavage specificity

Lidiya Lisitskaya, Yeonoh Shin, Aleksei Agapov, Anna Olina,
Ekaterina Kropocheva, Sergei Ryazansky, Alexei A. Aravin, Daria Esyunina,
Katsuhiko S. Murakami, Andrey Kulbachinskiy

Supplementary Information

Contains 9 Supplementary Figures and 2 Supplementary Tables

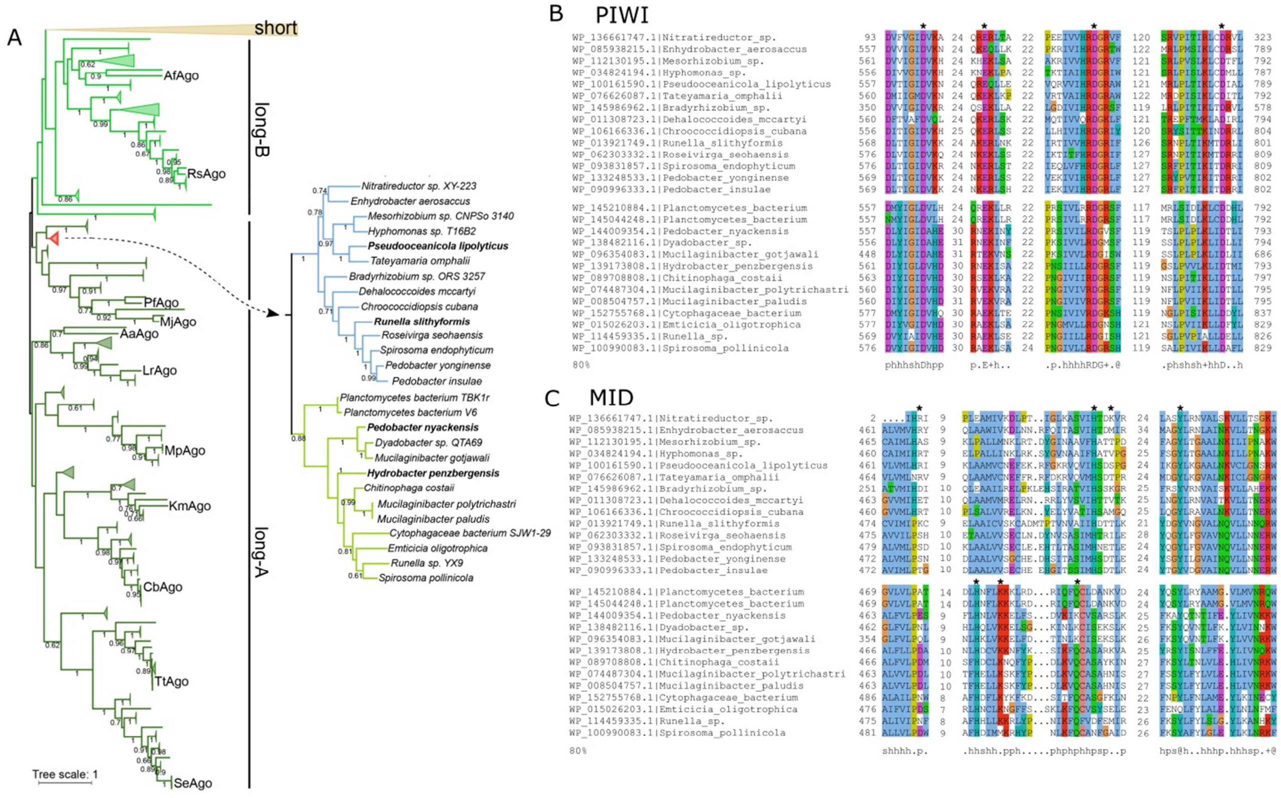

Supplementary Fig. 1. Phylogenetic analysis of D-R pAgos. (A) Phylogenetic tree showing positions of previously analysed pAgos and of the new class of D-R pAgos. The ‘Long-A’ clade of pAgos mainly includes catalytically active full-length pAgos, the ‘long-B’ clade includes catalytically inactive full-length pAgos with substitutions in the catalytic tetrad, the ‘short’ clade includes catalytically inactive pAgos containing the PIWI and MID domains but lacking the N-terminal and PAZ domains (Ryazansky et al., 2018). The PliAgo and PnyAgo groups are shown in blue and green, respectively; host bacterial species are indicated. The previously studied pAgos shown on the tree include RsAgo from *Rhodobacter sphaeroides*, AfAgo from *Archaeoglobus fulgidus*, MjAgo from *Methanocaldococcus jannaschii*, PfAgo from *Pyrococcus furiosus*, SeAgo from *Synechococcus elongatus*, TtAgo from *Thermus thermophilus*, KmAgo from *Kurthia massiliensis*, CbAgo from *Clostridium butyricum*, MpAgo from *Marinitoga piezophila*, LrAgo from *Limnothrix rosea* and AaAgo from *Aquifex aeolicus*. (B) Alignment of the active site residues in the PIWI domain in D-R pAgos in the two groups of D-R pAgos (PliAgo group, top; PnyAgo group, bottom). The catalytic tetrad residues are indicated with asterisks, the coloring is based on the consensus shown underneath the alignment. (C) Alignment of the MID pocket sequences in the two groups of D-R pAgos. The residues involved in contacts with the 5’-phosphate in PliAgo (top) and in the canonical MID pocket (bottom) are indicated with asterisks.

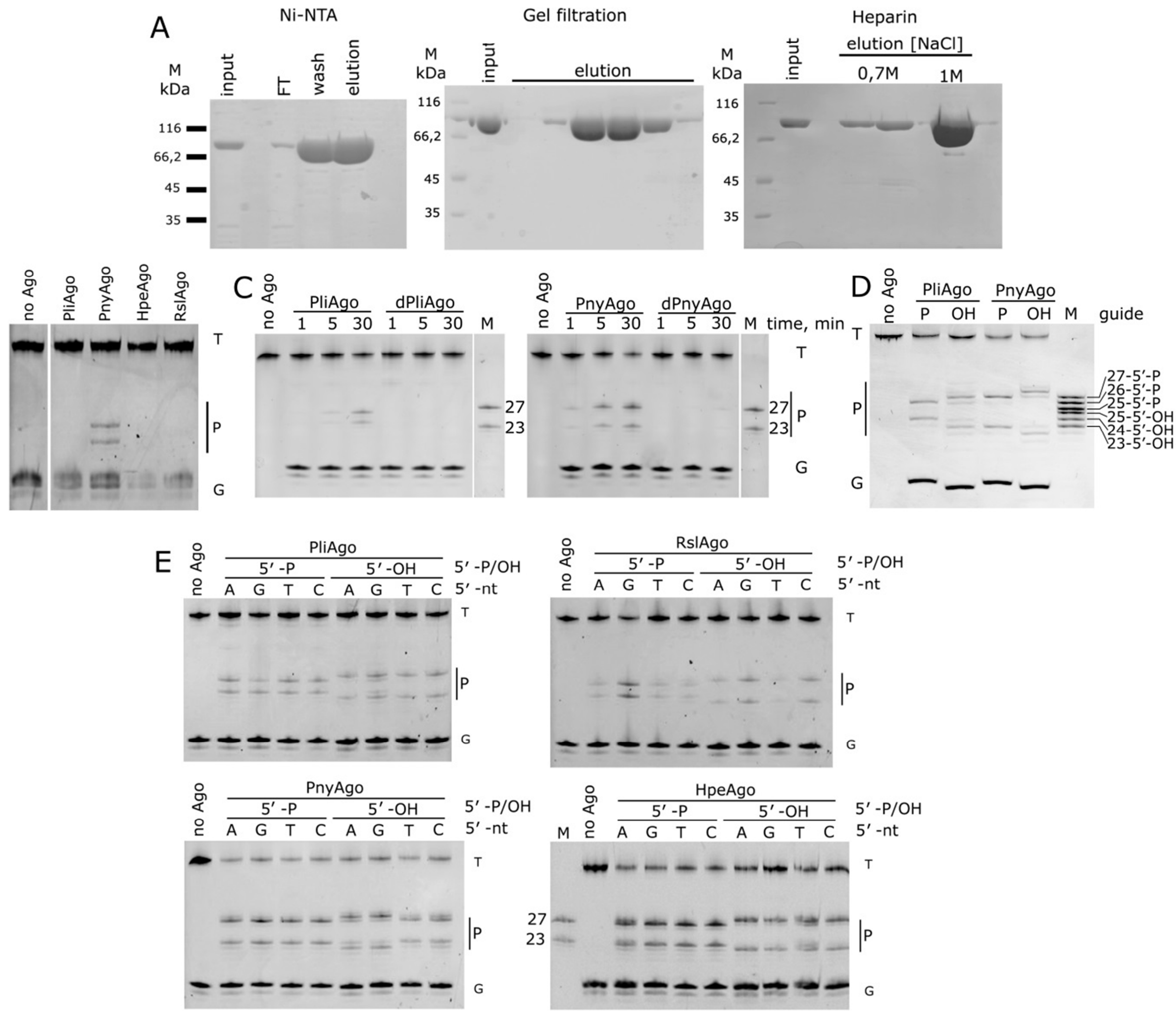

Supplementary Fig. 2. Purification and characterization of D-R pAgos. (A) Purification of PliAgo by Ni-NTA-affinity, Superose-6 gel filtration and heparin chromatography steps. For each step, the input and the elution fractions are shown (SDS-PAGE, Coomassie staining). Representative gels from two independent experiments are shown. (B) Analysis of target DNA cleavage by indicated pAgos after prolonged incubation (3 hours) at 37 °C (SYBR Gold staining). A representative gel from two independent experiments is shown. (C) Comparison of RNA cleavage by wild-type and catalytically dead dPliAgo (substitutions D563A, D626A) and dPnyAgo (D563A, D632A) (SYBR Gold staining). Representative gels from two independent experiments. (D) Determination of the position of the cleavage site for PliAgo and PnyAgo. Representative gel from two independent experiments. Synthetic oligonucleotides with the same sequence as the RNA target were used as size markers for the expected cleavage products corresponding to the 5'-part of the RNA target (23-5'-OH, 24-5'-OH and 25-5'-OH) and 5'-phosphorylated 3'-part of the target (27-5'-P, 26-5'-P, 25-5'-P) (lane M). The main cleavage products observed for PliAgo and PnyAgo coincide with the 24-5'-OH and 26-5'-P markers and 23-5'-OH and 27-5'-P markers, respectively, corresponding to cleavage between target positions 9' and 10' for PliAgo and positions 10' and 11' for PnyAgo. (E) Activities of the pAgo proteins with 5'-P and 5'-OH guide DNAs containing various 5' nucleotides. The results of a single experiment are shown. The reactions in panels were performed with 18 nt guide DNAs for 1 hour at 37 °C. Positions of targets ('T'), guides ('G') and reaction products ('P') are indicated.

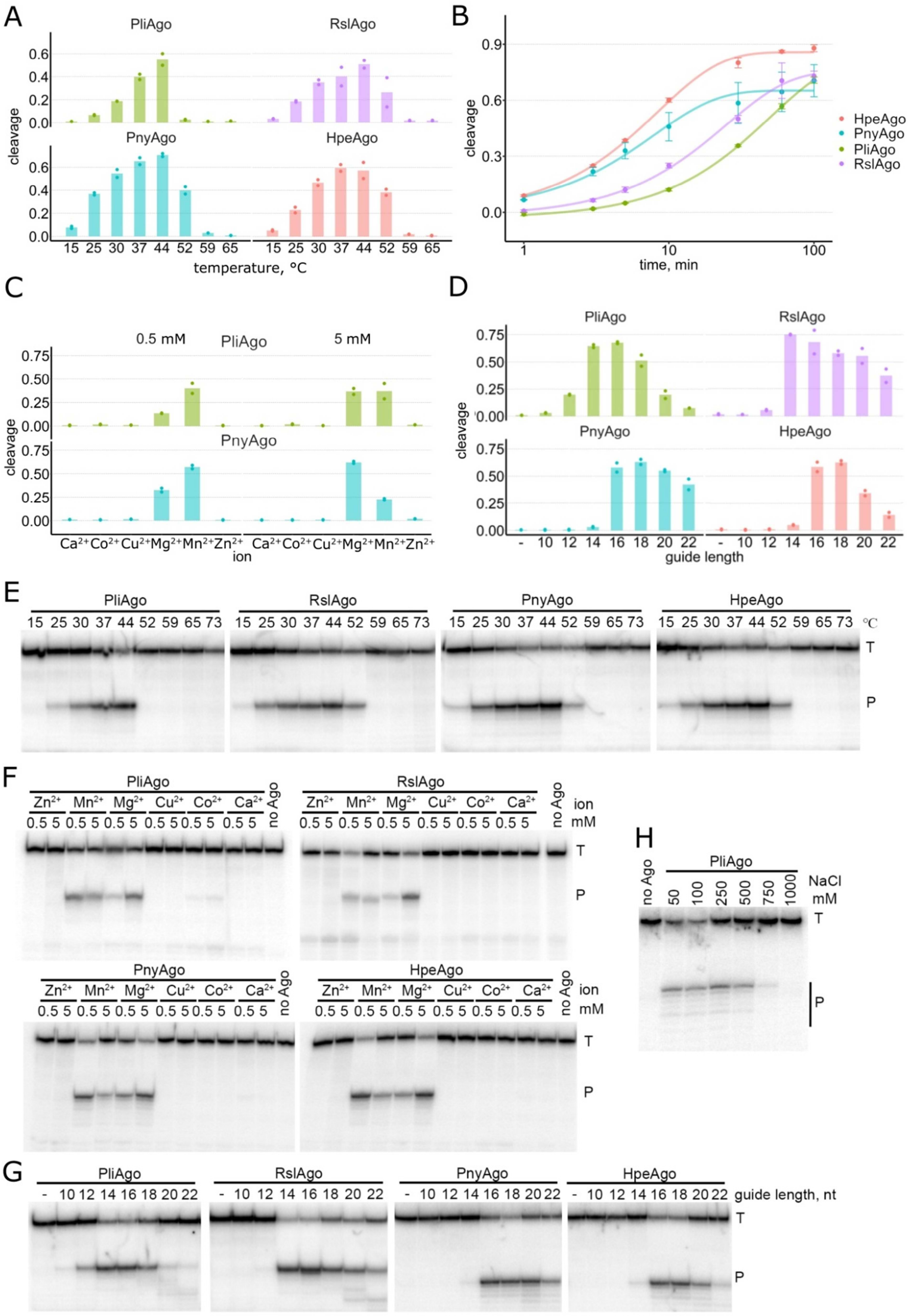
A
PliAgo
RslAgo
PnyAgo
HpeAgo
cleavage
temperature, °C
B
cleavage
time, min
HpeAgo
PnyAgo
PliAgo
RslAgo
C
0.5 mM
PliAgo
5 mM
PnyAgo
cleavage
ion
D
PliAgo
RslAgo
PnyAgo
HpeAgo
cleavage
guide length
E
PliAgo
RslAgo
PnyAgo
HpeAgo
15 25 30 37 44 52 59 65 73
°C
T
P
F
PliAgo
RslAgo
PnyAgo
HpeAgo
Zn²⁺ Mn²⁺ Mg²⁺ Cu²⁺ Co²⁺ Ca²⁺
no Ago
ion
mM
T
P
H
no Ago
PliAgo
50 100 250 500 750 1000
NaCl
mM
T
P
G
PliAgo
RslAgo
PnyAgo
HpeAgo
- 10 12 14 16 18 20 22
guide length, nt
T
P

Supplementary Fig. 3. Biochemical activities of D-R pAgos. (A) and (E) Temperature dependence of the pAgo activity. (B) Kinetics of RNA cleavage by PliAgo, RslAgo, HpeAgo and PnyAgo under single-turnover conditions (500 nM pAgo, 200 nM guide DNA, 100 nM target RNA) at 37 °C. The rate constants for the cleavage reaction are 0.020 ± 0.002 min^{-1} for PliAgo, 0.040 ± 0.005 min^{-1} for RslAgo, 0.12 ± 0.02 min^{-1} for PnyAgo and 0.13 ± 0.02 min^{-1} for HpeAgo (0.95 confidence intervals). (C) and (F) Target RNA cleavage by PliAgo and PnyAgo with different divalent cations added to 0.5 mM or 5 mM concentrations. (D) and (G) Efficiency of RNA cleavage by the four pAgos with DNA guides of various lengths. (H) RNA cleavage by PliAgo at different concentrations of NaCl. Positions of targets ('T'), guides ('G') and reactions products ('P') are indicated. The reactions were performed with 18 nt guide DNAs (or with guides of various lengths) for 1 hour at 37 °C (or other temperatures) as indicated. In panels (A), (C) and (D), means from two independent experiments are shown. In panel (B), means from three independent experiments are shown; error bars correspond to standard deviations. In panels (E), (F), (G), (H), representative gels from two independent experiments are shown.

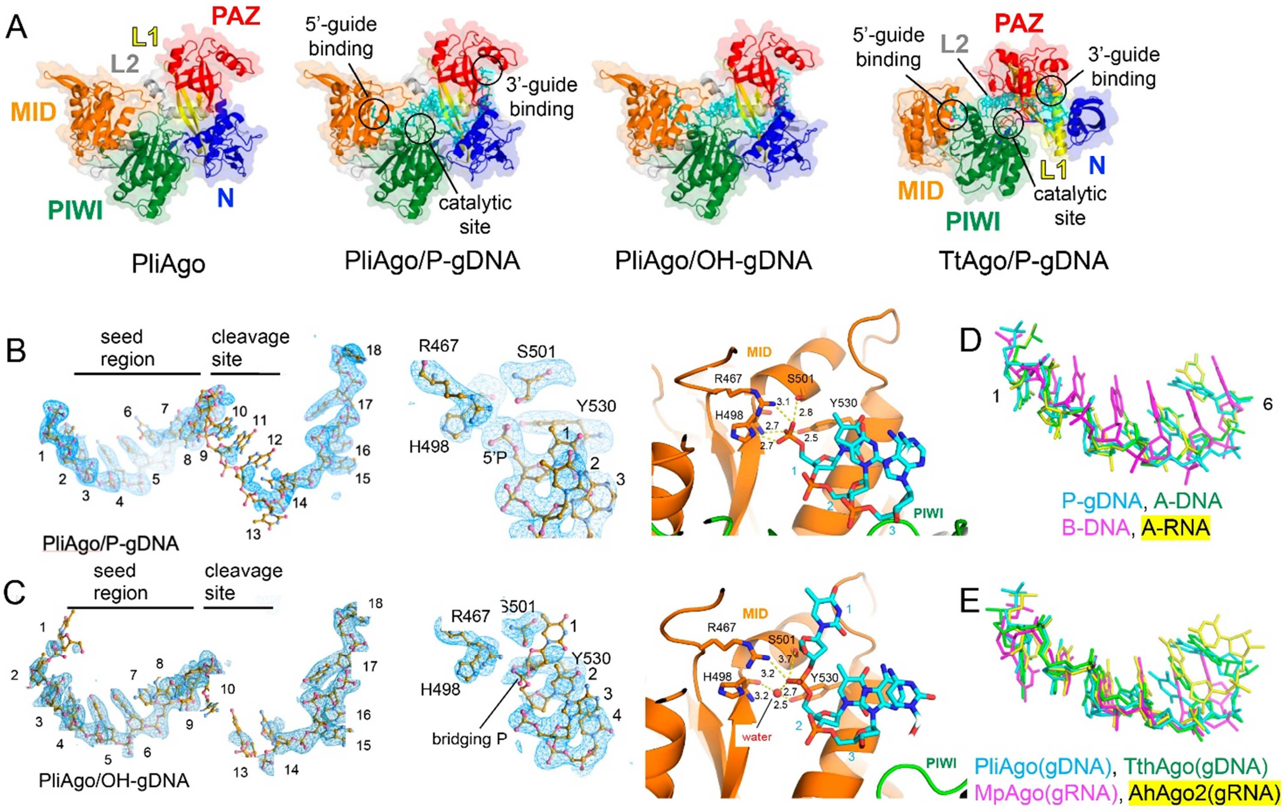

Supplementary Fig. 4. X-ray crystal structures of PliAgo and its complexes with guide DNA. (A) Comparison of X-ray crystal structures of PliAgo and TtAgo. X-ray crystal structures of PliAgo in the apo-form, in the complex with P-gDNA complex, in the complex with OH-gDNA and X-ray crystal structure of the TtAgo-gDNA-tDNA complex (PDB: 4NCA) are depicted as ribbon (Agos) and stick models (DNA) with partially transparent surfaces. Domains are colored and labelled as in Fig. 2. (B, C) PliAgo and gDNA interactions. (*Left*) Simulated annealing electron density omit maps (light blue mesh, sigma cutoff=4.5) of gDNA in the PliAgo/P-gDNA (B) and the PliAgo/OH-gDNA complexes (C) (overlaid with stick models of gDNA). Functional regions of gDNA are indicated. (*Middle*) Simulated annealing electron density omit maps (light blue mesh, omitted gDNA and amino acid residues around DNA) of the 5'-segment of gDNA in the PliAgo/P-gDNA (B) and the PliAgo/OH-gDNA complexes (C) (overlaid with stick models of gDNA and amino acid residues). (*Right*) Interactions of the MID domain (ribbon, orange) with the 5'-phosphate of gDNA (stick model) in PliAgo/P-gDNA (B) and the bridging phosphate of gDNA (stick model) in the PliAgo/OH-gDNA (C) complexes. The distances (Å) between the phosphate group, amino acid residues and water are indicated. (D) Overlay of the seed region (nucleotides 1-6) of gDNA in the PliAgo-P-gDNA complex (cyan) with A-form DNA (green), B-form DNA (magenta) and A-form RNA (yellow) using the DNA/RNA backbones. DNA and RNA structures were prepared by Coot. (E) Overlay of the seed region of gDNA in the PliAgo-P-gDNA complex (nucleotides 1-6, cyan) with gDNA in TtAgo (nucleotides 2-7, green; 3DLH), gRNA in hAgo2 (nucleotides 2-7, yellow; 4W5O) and gRNA in MpAgo (nucleotides 2-7, magenta; 5UX0) using the DNA/RNA backbones.

A

Ago	Mg^{2+}	K_d, nM
PnyAgo	+	31 ± 15 **1**
	-	390 ± 74 **13**
dPnyAgo	+	25 ± 16 **0.82**
	-	170 ± 48 **5.5**
PliAgo	+	14 ± 4.3 **1**
	-	31 ± 15 **2.3**
dPliAgo	+	16 ± 5.5 **1.1**
	-	44 ± 5.2 **3.2**
PliAgo K759A	+	220 ± 28 **16**
	-	270 ± 15 **20**
PliAgo K565A	+	36 ± 18 **2.6**
PliAgo N566A	+	13 ± 6.7 **0.94**
PliAgo ΔC18	+	8.5 ± 3.3 **0.63**
	-	22 ± 4.1 **1.6**
PliAgo MID*	+	>1000
	-	>1000
PliAgo-GFP	+	24 ± 7.6 **1.7**
CbAgo	+	4.0 ± 2.2 **1**
CbAgo ΔC12	+	180 ± 66 **44**

B

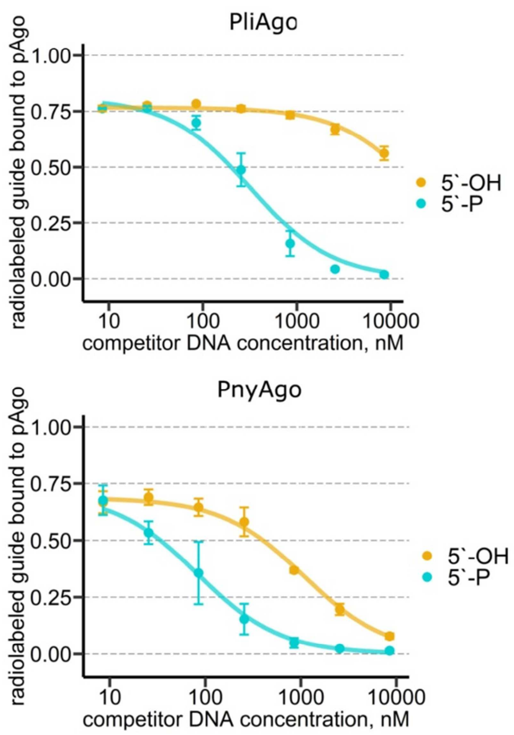

C

Ago	P/OH	K_d, nM
PliAgo	P	41 ± 13 **1**
	OH	>1000
PnyAgo	P	21 ± 12 **1**
	OH	250 ± 24 **12**

D

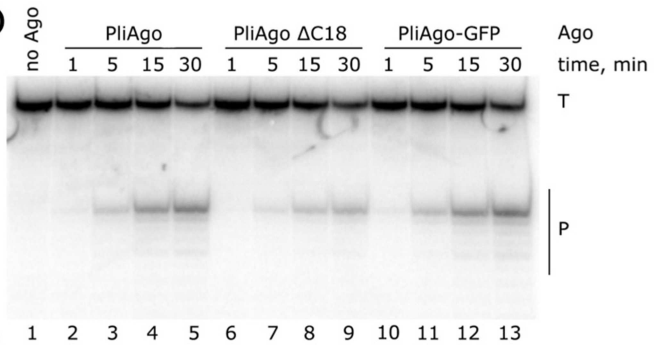

E

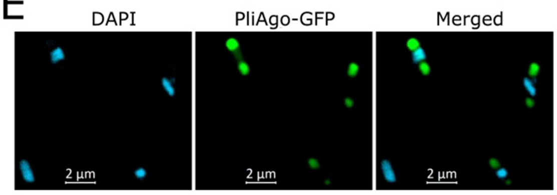

Supplementary Fig. 5. Analysis of guide DNA binding by pAgo proteins and their mutant variants. (A) Apparent K_d values for interactions of guide DNA with wild-type and mutant PnyAgo, PliAgo and CbAgo proteins measured in direct binding experiments by dot-blotting. Means and 0.95 confidence intervals from 3 or 4 independent experiments are shown. The numbers in bold show fold-changes in the apparent K_d values relative to wild-type pAgos. The data were used to calculate statistical significance (p-values) for the observed differences between K_ds for the wild-type and mutant pAgo variants, presented in the main manuscript text. P-values were calculated using the two-tailed Student t-test for independent variables without adjustments for multiple comparisons. (B) Competitive titration of 5'-P^{32}-labeled guide DNA with unlabeled 5'-P or 5'-OH guides for PliAgo (top) and PnyAgo (bottom). The fraction of bound radiolabeled DNA is shown (the ratio of bound DNA to the total amount of labeled DNA in the sample). Means from 3 independent experiments are shown; error bars correspond to standard deviations. (C) Apparent K_d values for interactions of 5'-P and 5'-OH guide DNA with PliAgo and PnyAgo measured in competition experiments. Means and 0.95 confidence intervals from 3 independent experiments are shown. (D) Effects of mutations in the C-terminus of PliAgo on the kinetics of RNA cleavage. A representative gel from three independent experiments is shown. (E) Expression of PliAgo-GFP in *E. coli* cells, visualized by confocal microscopy (merged with DAPI staining of chromosomal DNA).

A

Ago	target	K_d, nM
PnyAgo	RNA	7.5 ± 3.8 **1**
	DNA	0.27 ± 0.1 **1**
dPnyAgo	RNA	13 ± 3,0 **1.8**
	DNA	0.73 ± 0.25 **2.7**
PliAgo	RNA	0.32 ± 0.18 **1**
	DNA	0.58 ± 0.1 **1**
dPliAgo	RNA	0.53 ± 0.22 **1.7**
	DNA	0.99 ± 0.29 **1.7**
PliAgo K759A	RNA	5.3 ± 3.2 **16**
	DNA	5.0 ± 1.4 **8.5**
PliAgo K565A	RNA	1.1 ± 0.12 **3.4**
	DNA	1.8 ± 0.43 **3.1**
PliAgo N566A	RNA	0.54 ± 0.22 **1.7**
	DNA	0.90 ± 0.3 **1.5**
PliAgo ΔC18	RNA	0.33 ± 0.07 **1.03**
	DNA	0.28 ± 0.16 **0.48**
PliAgo-GFP	RNA	1.2 ± 0.25 **3.6**
	DNA	1.7 ± 0.95 **2.8**
CbAgo	RNA	>100
	DNA	0.23 ± 0.07 **1**

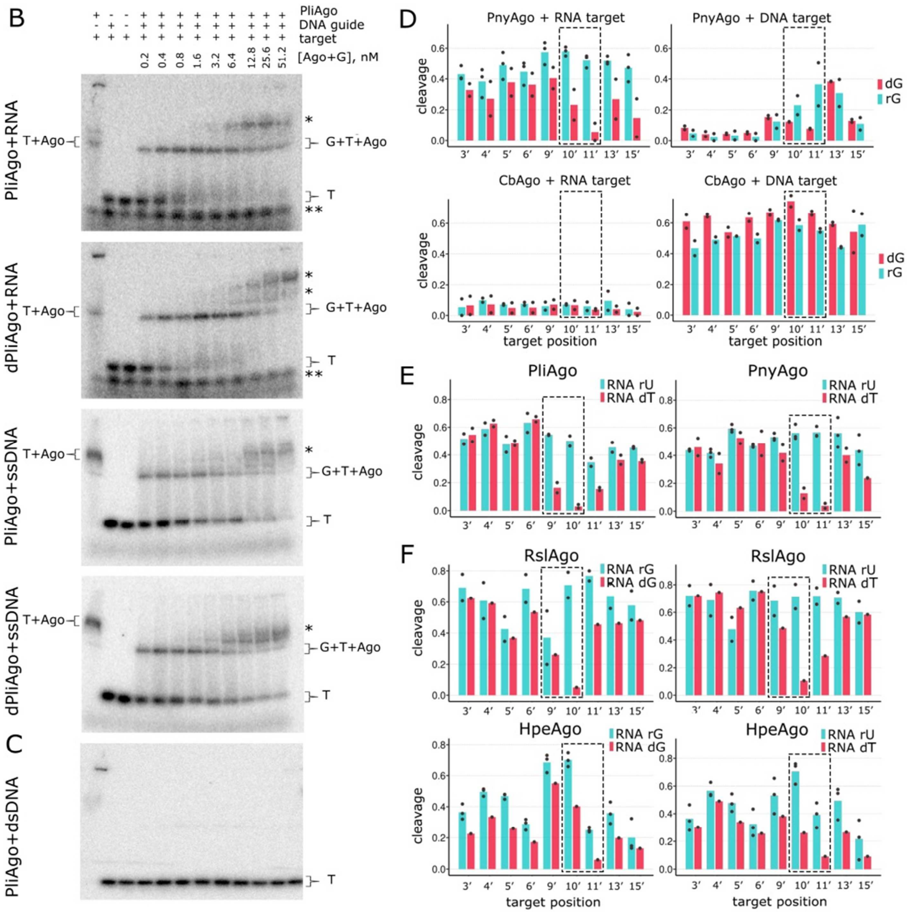

Supplementary Fig. 6. Analysis of target RNA or DNA binding by binary guide-pAgo complexes. (A) Apparent K_d values for the binding of target DNA or RNA (5'-P^{32}-labeled) by wild-type and mutant pAgos measured by dot-blotting. Means and 0.95 confidence intervals from 3 independent experiments are shown. The numbers in bold show fold-changes in the apparent K_d values relative to wild-type pAgos. The data were used to calculate statistical significance (p-values) for the observed differences between K_ds for the wild-type and mutant pAgo variants, presented in the main manuscript text. P-values were calculated using the two-tailed Student t-test for independent variables without adjustments for multiple comparisons. (B) Analysis of the formation of ternary guide-target-pAgo complexes by EMSA (with 5'-P^{32}-labeled target RNA or ssDNA). Positions of free target RNA or DNA ('T'), nonspecific target-pAgo complexes (T+Ago) formed in the absence of guide DNA, and of ternary guide-target-pAgo complexes ('G+T+pAgo') are indicated. Single asterisks indicate higher molecule weight complexes observed at high concentrations of pAgo, which may be formed as a result of nonspecific binding of additional pAgo molecules to the ternary complex. Formation of the higher-order low mobility complexes may possibly explain why the efficiency of target RNA cleavage by PliAgo is <100% in the cleavage assays. Double asterisks indicate a high mobility band observed in the case of the RNA target that may possibly correspond to residual γ-P^{32}-ATP used for target labeling or to a structured conformer of the RNA target not bound by PliAgo. (C) Analysis of interactions of PliAgo with dsDNA (5'-P^{32}-labeled in the target strand) by EMSA. The double-stranded target was obtained by pre-annealing of ssDNA of the same sequence as in panel B with a complementary oligonucleotide. In panels (B) and (C), representative gels from 2 independent experiments are shown. (D) Effects of a single deoxyribonucleotide (dG) in the RNA target (left) and a single ribonucleotide (rG) in the DNA target (right) on target cleavage by PnyAgo and CbAgo, in comparison with control RNA and DNA targets. The reactions were performed with a set of differently positioned guide DNAs and a 5'-labeled RNA target (see Supplementary Table 1 for oligonucleotide sequences). Position of the deoxyribonucleotide residue relative to the guide 5'-end is indicated. The reactions were performed at 37 °C for 30 minutes in the case of target RNA and for 3 hours in the case of target DNA. In each reaction, the cleavage efficiency was calculated as the ratio of the cleaved RNA product to

the sum of the product and unreacted target. For PnyAgo, considerable cleavage of the control DNA target is also observed with several guides (for positions 9'-15', upper right panel), confirming that it can cleave DNA under certain conditions (see Supplementary Fig. 2B). (E) Target RNA cleavage by PliAgo and PnyAgo for an RNA target containing a single deoxythymidine (dU) residue at indicated positions, in comparison with a control template (rU). (F) Effects of single deoxyribonucleotides (dG, left; dU, right) in the RNA target on its cleavage by RslAgo and HpeAgo, in comparison with corresponding control RNA targets. Target positions around the cleavage site for each pAgo are shown with dotted boxes. The reactions were performed at 37 °C for 30 minutes in the case of RslAgo and 10 minutes in the case of HpeAgo. In panels (D), (E), (F), means from 2-3 independent measurements are shown (the number of measurements is shown as individual data points on each graph).

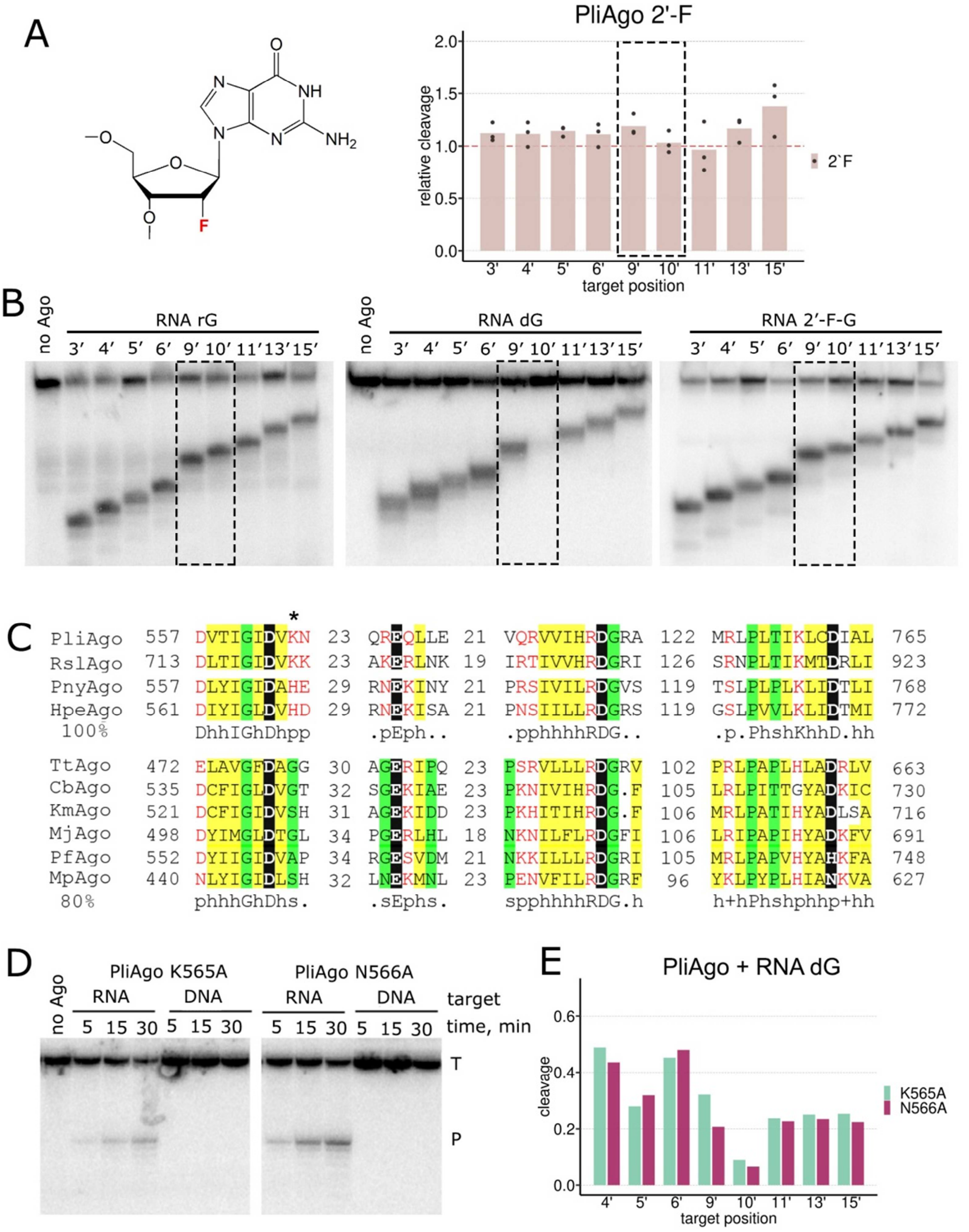

Supplementary Fig. 7. Effects of the 2'-fluoro modification and of PliAgo mutations on target cleavage. (A) Analysis of the effect of the 2'-F-guanosine modification (shown on the left) on the efficiency of RNA cleavage relative to the unmodified RNA target (right). Means from 3 independent measurements are shown. (B) Electrophoretic analysis of the cleavage products of modified RNAs containing 2'-dG or 2'-F-G at indicated positions by PliAgo in comparison with the control unmodified target (rG) (representative gels from 3 independent experiments). The reactions were performed with 5'-P[32]-labeled target RNA and differently positioned guide DNAs, resulting in different positioning of the modified residues relative to the active site (see Fig. 4 and Supplementary Table 1). (C) The consensus sequence of the active site in the PIWI domain in the two groups of D-R pAgos and in previously analysed pAgos. The coloring is based on the consensus shown underneath the alignment (see Fig. 3 for color scheme). The catalytic tetrad residues are shown in black, the PliAgo-specific residue K565 is indicated with an asterisk. (D) Analysis of target RNA and DNA cleavage by the K565A and N566A mutants of PliAgo. The results of a single experiment are shown. (E) Cleavage of the RNA target containing a single deoxyribonucleotide (dG) at indicated positions (defined by guide DNAs) by the K565A and N566A mutants. The results of a single experiment are shown. The reaction was performed at 37 °C for 30 minutes. Target positions around the cleavage site are shown with dotted boxes.

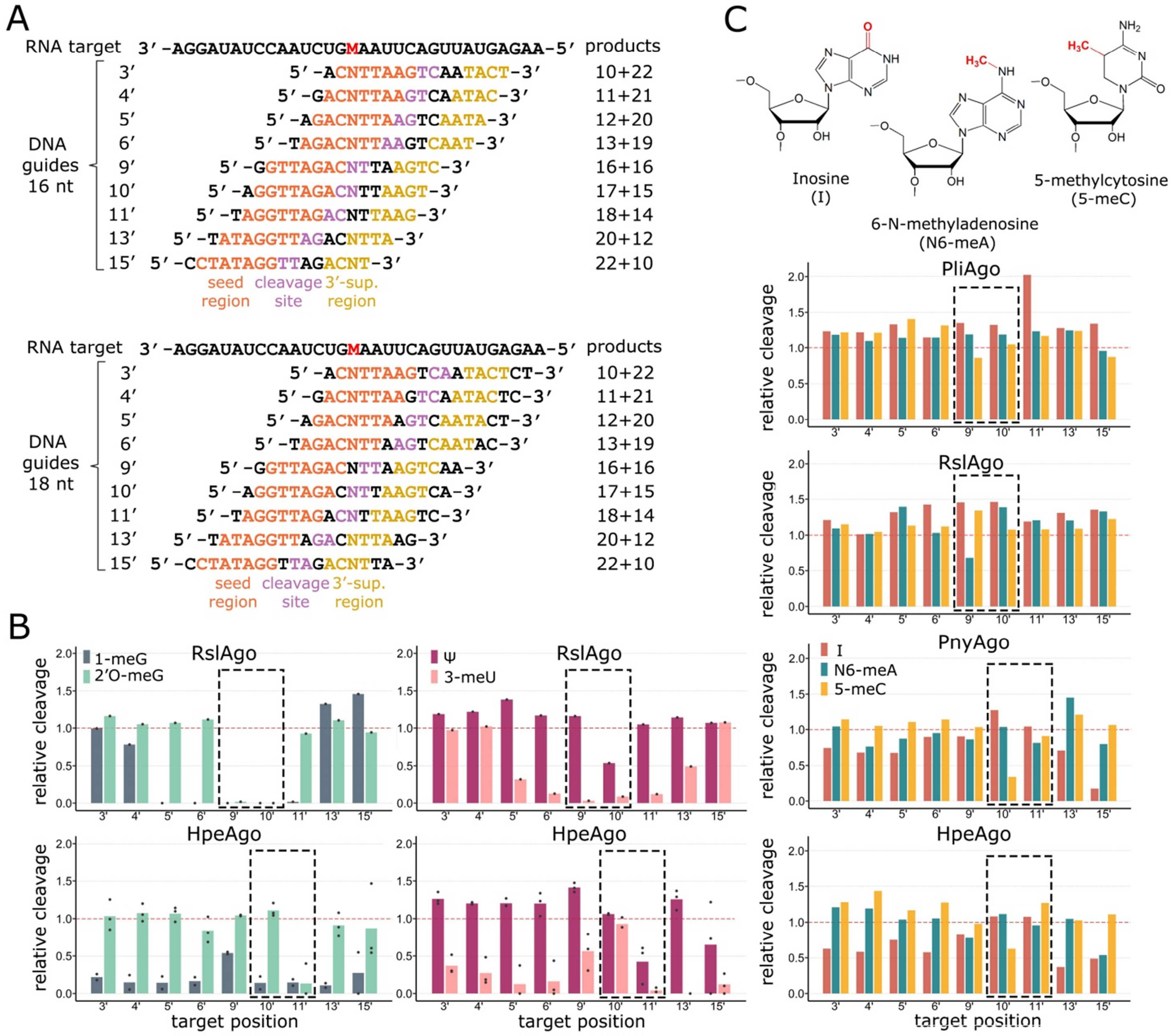

Supplementary Fig. 8. Effects of site-specific modifications on target RNA cleavage by pAgos. (A) RNA targets containing modified nucleotides (M) and differently positioned DNA guides used in the cleavage reactions. 'N' indicates complementary nucleotide depending on the type of modification. The functional regions in guide DNA (seed, central and 3'-supplementary) are indicated. The experiments were performed with 16 nt guide DNAs (top) for PliAgo and RslAgo and 18 nt guide DNAs (bottom) for PnyAgo and HpeAgo. (B) Effects of the 1-meG, 2'O-meG, Ψ and 3-meU modifications on target RNA cleavage by RslAgo and HpeAgo. For RslAgo, the results of a single experiment are presented; for HpeAgo, means from 3 independent measurements are shown. (C) Analysis of target RNA cleavage with the I, N^6-meA and 5-meC modifications (shown on the top) by PliAgo, RslAgo, PnyAgo and HpeAgo. For each position, the efficiency of cleavage of modified RNA relative to the efficiency of cleavage of control RNA measured with the same guide DNA ('relative cleavage'), is shown. In panel (C), the experiment was performed one time for each pAgo. Target positions around the cleavage site are shown with dotted boxes.

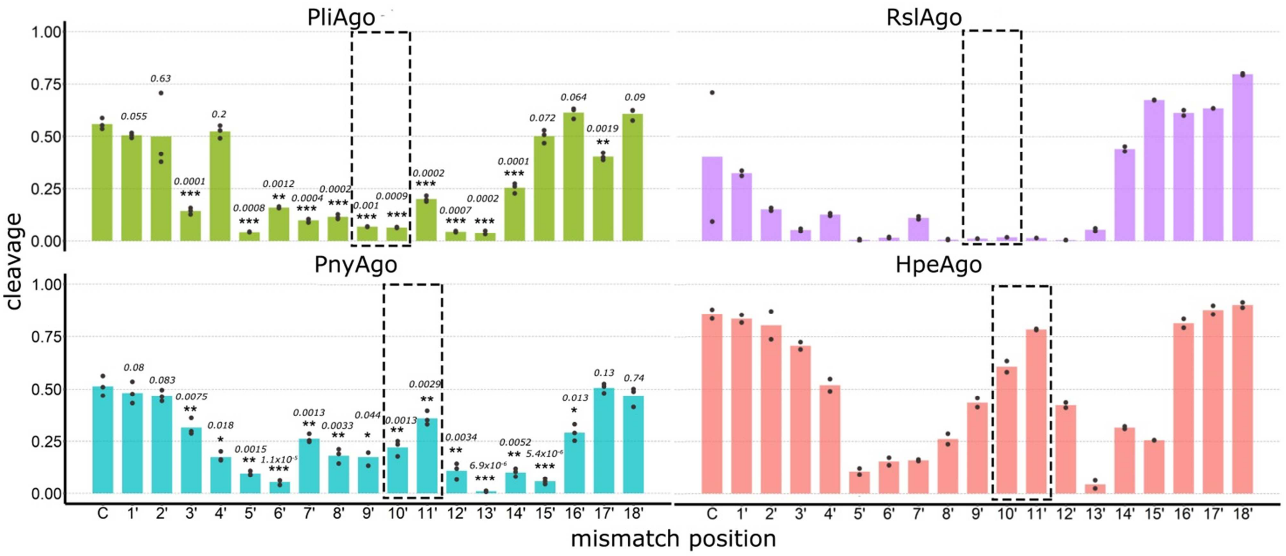

Supplementary Fig. 9. Effects of single-nucleotide mismatches on target RNA cleavage by pAgos. Quantification of the effects of mismatches on the efficiency of target RNA cleavage by D-R pAgos (Fig. 6). Target positions around the cleavage site are shown with dotted boxes. For each guide DNA, cleavage efficiencies were calculated as the ratio of the amounts of reaction products to the sum of reaction products and unreacted RNA. Means from 3 (for PliAgo and PnyAgo) or 2 (for RslAgo and HpeAgo) independent experiments are shown. For PliAgo and PnyAgo, significant changes in the cleavage efficiencies in comparison with the fully complementary control guide DNA ('C') are indicated (p-values: *<0.05; **<0.01; ***<0.001; the exact p-values for individual mismatches are indicated on the figure). The p-values were calculated by the two-tailed Student t-test for independent variables without adjustments for multiple comparisons.

Supplementary Table 1. Sequences of oligonucleotides.

	**Cleavage and binding assays**		
	Oligonucleotide name	Sequence (5'-3')	Description
1.	G-guide-DNA	GTTAGACTTTAAGTCAAT	18 nt guide DNA with 5'-G, complementary to G-target
2.	A-guide-DNA	ATTAGACTTTAAGTCAAT	18 nt guide DNA with 5'-A, complementary to G-target
3.	T-guide-DNA	TTTAGACTTTAAGTCAAT	18 nt guide DNA with 5'-T, complementary to G-target
4.	C-guide-DNA	CTTAGACTTTAAGTCAAT	18 nt guide DNA with 5'-C, complementary to G-target
5.	G-target-DNA	TTTATCAAAAAGAGTATTGA<br>CTTAAAGTCTAACCTATAGG<br>ATACTTACAG	50 nt target DNA for the guide/target specificity assay
6.	G-guide-RNA	GUUAGACUUUAAGUCAAU	18 nt guide RNA for the guide/target specificity assay
7.	G-target-RNA	UUUAUCAAAAAGAGUAUUG<br>ACUUAAAGUCUAACCUAUA<br>GGAUACUUACAG	50 nt target RNA for the guide/target specificity assay
8.	G-guide-10nt	GTTAGACTTT	10 nt guide with 5'-G, complementary to G-target
9.	G-guide-12nt	GTTAGACTTTAA	12 nt guide with 5'-G, complementary to G-target
10.	G-guide-14nt	GTTAGACTTTAAGT	14 nt guide with 5'-G, complementary to G-target
11.	G-guide-16nt	GTTAGACTTTAAGTCA	16 nt guide with 5'-G, complementary to G-target
12.	G-guide-20nt	GTTAGACTTTAAGTCAATAC	20 nt guide with 5'-G, complementary to G-target
13.	G-guide-22nt	GTTAGACTTTAAGTCAATAC<br>TC	22 nt guide with 5'-G, complementary to G-target
14.	Marker_5_RNA23	UUUAUCAAAAAGAGUAUUG<br>ACUU	23 nt RNA used as RNA marker
15.	Marker_5_RNA24	UUUAUCAAAAAGAGUAUUG<br>ACUUA	24 nt RNA used as RNA marker
16.	Marker_5_RNA25	UUUAUCAAAAAGAGUAUUG<br>ACUUAA	25 nt RNA used as RNA marker
17.	Marker_3_RNA25	AGUCUAACCUAUAGGAUAC<br>UUACAG	25 nt RNA used as RNA marker
18.	Marker_3_RNA26	AAGUCUAACCUAUAGGAUA<br>CUUACAG	26 nt RNA used as RNA marker
19.	Marker_3_RNA27	AAAGUCUAACCUAUAGGAU<br>ACUUACAG	27 nt RNA used as RNA marker
	**Crystallization**		
20.	3D-guide	TTACTGCACAGGTGACGA	18 nt guide with 5'-T used for crystallization
21.	3D-target-RNA	CUGUCGUCACCUGUGCAG<br>UAACUG	24 nt target RNA for 3D-guide, tested in crystallization trials
22.	3D-target-DNA	CATCTAGTAACTGCTGTCGT<br>CACCTGTGCAGTAACTGAG<br>TCATGGACTAA	50 nt target DNA for 3D-guide, used in the cleavage assay in Fig. S2B
	**Mismatch assay**		
23.	G-guide_mm1	CTTAGACTTTAAGTCAAT	guide forms mismatched pair in

			position 1 with G-target
24.	G-guide_mm2	GATAGACTTTAAGTCAAT	guide forms mismatched pair in position 2 with G-target
25.	G-guide_mm3	GTAAGACTTTAAGTCAAT	guide forms mismatched pair in position 3 with G-target
26.	G-guide_mm4	GTTTGACTTTAAGTCAAT	guide forms mismatched pair in position 4 with G-target
27.	G-guide_mm5	GTTACACTTTAAGTCAAT	guide forms mismatched pair in position 5 with G-target
28.	G-guide_mm6	GTTAGTCTTTAAGTCAAT	guide forms mismatched pair in position 6 with G-target
29.	G-guide_mm7	GTTAGAGTTTAAGTCAAT	guide forms mismatched pair in position 7 with G-target
30.	G-guide_mm8	GTTAGACATTAAGTCAAT	guide forms mismatched pair in position 8 with G-target
31.	G-guide_mm9	GTTAGACTATAAGTCAAT	guide forms mismatched pair in position 9 with G-target
32.	G-guide_mm10	GTTAGACTTAAAGTCAAT	guide forms mismatched pair in position 10 with G-target
33.	G-guide_mm11	GTTAGACTTTTAGTCAAT	guide forms mismatched pair in position 11 with G-target
34.	G-guide_mm12	GTTAGACTTTATGTCAAT	guide forms mismatched pair in position 12 with G-target
35.	G-guide_mm13	GTTAGACTTTAACTCAAT	guide forms mismatched pair in position 13 with G-target
36.	G-guide_mm14	GTTAGACTTTAAGACAAT	guide forms mismatched pair in position 14 with G-target
37.	G-guide_mm15	GTTAGACTTTAAGTGAAT	guide forms mismatched pair in position 15 with G-target
38.	G-guide_mm16	GTTAGACTTTAAGTCTAT	guide forms mismatched pair in position 16 with G-target
39.	G-guide_mm17	GTTAGACTTTAAGTCATT	guide forms mismatched pair in position 17 with G-target
40.	G-guide_mm18	GTTAGACTTTAAGTCAAA	guide forms mismatched pair in position 18 with G-target
**Modifications assay**			
41.	RNA_t_rA	AAGAGUAUUGACUUAA**A**GU CUAACCUAUAGGA	32 nt RNA target with A in position 17
42.	RNA_t_rU	AAGAGUAUUGACUUAA**U**GU CUAACCUAUAGGA	32 nt RNA target with U in position 17
43.	RNA_t_rG	AAGAGUAUUGACUUAA**G**GU CUAACCUAUAGGA	32 nt RNA target with G in position 17
44.	RNA_t_rC	AAGAGUAUUGACUUAA**C**GU CUAACCUAUAGGA	32 nt RNA target with C in position 17
45.	RNA_t_I	AAGAGUAUUGACUUAA**(I)**GU CUAACCUAUAGGA	32 nt RNA target with I in position 17
46.	RNA_t_N6-meA	AAGAGUAUUGACUUAA(**N6-meA)**GUCUAACCUAUAGGA	32 nt RNA target with N6-meA in position 17
47.	RNA_t_5-meC	AAGAGUAUUGACUUAA**(5-meC)**GUCUAACCUAUAGGA	32 nt RNA target with 5-meC in position 17
48.	RNA_t_Ψ	AAGAGUAUUGACUUAA**(Ψ)**G UCUAACCUAUAGGA	32 nt RNA target with Ψ in position 17
49.	RNA_t_2'O-meG	AAGAGUAUUGACUUAA**(2'O-**	32 nt RNA target with 2'-OmeG in

		**meG)**GUCUAACCUAUAGGA	position 17
50.	RNA_t_2'F-G	AAGAGUAUUGACUUAA**(2'-F-G)**GUCUAACCUAUAGGA	32 nt RNA target with 2'- F-G in position 17
51.	RNA_t_1-meG	AAGAGUAUUGACUUAA**(1-meG)**GUCUAACCUAUAGGA	32 nt RNA target with 1-meG in position 17
52.	RNA_t_3-meU	AAGAGUAUUGACUUAA**(3-meU)**GUCUAACCUAUAGGA	32 nt RNA target with 3-meU in position 17
53.	RNA_t_dG	AAGAGUAUUGACUUAA***dG***G UCUAACCUAUAGGA	32 nt RNA target with dG in position 17
54.	RNA_t_dU	AAGAGUAUUGACUUAA***dT***G UCUAACCUAUAGGA	32 nt RNA target with dT in position 17
55.	DNA_t_rG	AAGAGTATTGACTTAA***rG***GT CTAACCTATAGGA	32 nt DNA target with rG in position 17
56.	DNA_t_dG	AAGAGTATTGACTTAA**G**GTC TAACCTATAGGA	32 nt DNA target with G in position 17
57.	gDNA_18_3_A	AC**A**TTAAGTCAATACTCT	18 nt DNA guide with A in position 3 opposite the site of modification
58.	gDNA_18_4_A	GAC**A**TTAAGTCAATACTC	18 nt DNA guide with A in position 4 opposite the site of modification
59.	gDNA_18_5_A	AGAC**A**TTAAGTCAATACT	18 nt DNA guide with A in position 5 opposite the site of modification
60.	gDNA_18_6_A	TAGAC**A**TTAAGTCAATAC	18 nt DNA guide with A in position 6 opposite the site of modification
61.	gDNA_18_9_A	GGTTAGAC**A**TTAAGTCAA	18 nt DNA guide with A in position 9 opposite the site of modification
62.	gDNA_18_10_A	AGGTTAGAC**A**TTAAGTCA	18 nt DNA guide with A in position 10 opposite the site of modification
63.	gDNA_18_11_A	TAGGTTAGAC**A**TTAAGTC	18 nt DNA guide with A in position 11 opposite the site of modification
64.	gDNA_18_13_A	TATAGGTTAGAC**A**TTAAG	18 nt DNA guide with A in position 13 opposite the site of modification
65.	gDNA_18_15_A	CCTATAGGTTAGAC**A**TTA	18 nt DNA guide with A in position 15 opposite the site of modification
66.	gDNA_18_3_T	AC**T**TTAAGTCAATACTCT	18 nt DNA guide with T in position 3 opposite the site of modification
67.	gDNA_18_4_T	GAC**T**TTAAGTCAATACTC	18 nt DNA guide with T in position 4 opposite the site of modification
68.	gDNA_18_5_T	AGAC**T**TTAAGTCAATACT	18 nt DNA guide with T in position 5 opposite the site of modification
69.	gDNA_18_6_T	TAGACT**T**TAAGTCAATAC	18 nt DNA guide with T in position 6 opposite the site of modification
70.	gDNA_18_9_T	GGTTAGAC**T**TTAAGTCAA	18 nt DNA guide with T in position 9 opposite the site of modification
71.	gDNA_18_10_T	AGGTTAGAC**T**TTAAGTCA	18 nt DNA guide with T in position 10 opposite the site of modification
72.	gDNA_18_11_T	TAGGTTAGAC**T**TTAAGTC	18 nt DNA guide with T in position 11 opposite the site of modification
73.	gDNA_18_13_T	TATAGGTTAGAC**T**TTAAG	18 nt DNA guide with T in position 13 opposite the site of modification
74.	gDNA_18_15_T	CCTATAGGTTAGAC**T**TTA	18 nt DNA guide with T in position 15 opposite the site of modification
75.	gDNA_18_3_G	AC**G**TTAAGTCAATACTCT	18 nt DNA guide with G in position 3 opposite the site of modification

76.	gDNA_18_4_G	GAC**G**TTAAGTCAATACTC	18 nt DNA guide with G in position 4 opposite the site of modification
77.	gDNA_18_5_G	AGAC**G**TTAAGTCAATACT	18 nt DNA guide with G in position 5 opposite the site of modification
78.	gDNA_18_6_G	TAGAC**G**TTAAGTCAATAC	18 nt DNA guide with G in position 6 opposite the site of modification
79.	gDNA_18_9_G	GGTTAGAC**G**TTAAGTCAA	18 nt DNA guide with G in position 9 opposite the site of modification
80.	gDNA_18_10_G	AGGTTAGAC**G**TTAAGTCA	18 nt DNA guide with G in position 10 opposite the site of modification
81.	gDNA_18_11_G	TAGGTTAGAC**G**TTAAGTC	18 nt DNA guide with G in position 11 opposite the site of modification
82.	gDNA_18_13_G	TATAGGTTAGAC**G**TTAAG	18 nt DNA guide with G in position 13 opposite the site of modification
83.	gDNA_18_15_G	CCTATAGGTTAGAC**G**TTA	18 nt DNA guide with G in position 15 opposite the site of modification
84.	gDNA_18_3_C	AC**C**TTAAGTCAATACTCT	18 nt DNA guide with C in position 3 opposite the site of modification
85.	gDNA_18_4_C	GAC**C**TTAAGTCAATACTC	18 nt DNA guide with C in position 4 opposite the site of modification
86.	gDNA_18_5_C	AGAC**C**TTAAGTCAATACT	18 nt DNA guide with C in position 5 opposite the site of modification
87.	gDNA_18_6_C	TAGAC**C**TTAAGTCAATAC	18 nt DNA guide with C in position 6 opposite the site of modification
88.	gDNA_18_9_C	GGTTAGAC**C**TTAAGTCAA	18 nt DNA guide with C in position 9 opposite the site of modification
89.	gDNA_18_10_C	AGGTTAGAC**C**TTAAGTCA	18 nt DNA guide with C in position 10 opposite the site of modification
90.	gDNA_18_11_C	TAGGTTAGAC**C**TTAAGTC	18 nt DNA guide with C in position 11 opposite the site of modification
91.	gDNA_18_13_C	TATAGGTTAGAC**C**TTAAG	18 nt DNA guide with C in position 13 opposite the site of modification
92.	gDNA_18_15_C	CCTATAGGTTAGAC**C**TTA	18 nt DNA guide with C in position 15 opposite the site of modification
93.	gDNA_16_3_A	AC**A**TTAAGTCAATACT	16 nt DNA guide with A in position 3 opposite the site of modification
94.	gDNA_16_4_A	GAC**A**TTAAGTCAATAC	16 nt DNA guide with A in position 4 opposite the site of modification
95.	gDNA_16_5_A	AGAC**A**TTAAGTCAATA	16 nt DNA guide with A in position 5 opposite the site of modification
96.	gDNA_16_6_A	TAGAC**A**TTAAGTCAAT	16 nt DNA guide with A in position 6 opposite the site of modification
97.	gDNA_16_9_A	GGTTAGAC**A**TTAAGTC	16 nt DNA guide with A in position 9 opposite the site of modification
98.	gDNA_16_10_A	AGGTTAGAC**A**TTAAGT	16 nt DNA guide with A in position 10 opposite the site of modification
99.	gDNA_16_11_A	TAGGTTAGAC**A**TTAAG	16 nt DNA guide with A in position 11 opposite the site of modification
100.	gDNA_16_13_A	TATAGGTTAGAC**A**TTA	16 nt DNA guide with A in position 13 opposite the site of modification
101.	gDNA_16_15_A	CCTATAGGTTAGAC**A**T	16 nt DNA guide with A in position 15 opposite the site of modification
102.	gDNA_16_3_T	AC**T**TTAAGTCAATACT	16 nt DNA guide with T in position 3 opposite the site of modification

103.	gDNA_16_4_T	GAC**T**TTAAGTCAATAC	16 nt DNA guide with T in position 4 opposite the site of modification
104.	gDNA_16_5_T	AGAC**T**TTAAGTCAATA	16 nt DNA guide with T in position 5 opposite the site of modification
105.	gDNA_16_6_T	TAGAC**T**TTAAGTCAAT	16 nt DNA guide with T in position 6 opposite the site of modification
106.	gDNA_16_9_T	GGTTAGAC**T**TTAAGTC	16 nt DNA guide with T in position 9 opposite the site of modification
107.	gDNA_16_10_T	AGGTTAGAC**T**TTAAGT	16 nt DNA guide with T in position 10 opposite the site of modification
108.	gDNA_16_11_T	TAGGTTAGAC**T**TTAAG	16 nt DNA guide with T in position 11 opposite the site of modification
109.	gDNA_16_13_T	TATAGGTTAGAC**T**TTA	16 nt DNA guide with T in position 13 opposite the site of modification
110.	gDNA_16_15_T	CCTATAGGTTAGAC**T**T	16 nt DNA guide with T in position 15 opposite the site of modification
111.	gDNA_16_3_G	AC**G**TTAAGTCAATACT	16 nt DNA guide with G in position 3 opposite the site of modification
112.	gDNA_16_4_G	GAC**G**TTAAGTCAATAC	16 nt DNA guide with G in position 4 opposite the site of modification
113.	gDNA_16_5_G	AGAC**G**TTAAGTCAATA	16 nt DNA guide with G in position 5 opposite the site of modification
114.	gDNA_16_6_G	TAGAC**G**TTAAGTCAAT	16 nt DNA guide with G in position 6 opposite the site of modification
115.	gDNA_16_9_G	GGTTAGAC**G**TTAAGTC	16 nt DNA guide with G in position 9 opposite the site of modification
116.	gDNA_16_10_G	AGGTTAGAC**G**TTAAGT	16 nt DNA guide with G in position 10 opposite the site of modification
117.	gDNA_16_11_G	TAGGTTAGAC**G**TTAAG	16 nt DNA guide with G in position 11 opposite the site of modification
118.	gDNA_16_13_G	TATAGGTTAGAC**G**TTA	16 nt DNA guide with G in position 13 opposite the site of modification
119.	gDNA_16_15_G	CCTATAGGTTAGAC**G**T	16 nt DNA guide with G in position 15 opposite the site of modification
120.	gDNA_16_3_C	AC**C**TTAAGTCAATACT	16 nt DNA guide with C in position 3 opposite the site of modification
121.	gDNA_16_4_C	GAC**C**TTAAGTCAATAC	16 nt DNA guide with C in position 4 opposite the site of modification
122.	gDNA_16_5_C	AGAC**C**TTAAGTCAAT	16 nt DNA guide with C in position 5 opposite the site of modification
123.	gDNA_16_6_C	TAGAC**C**TTAAGTCAAT	16 nt DNA guide with C in position 6 opposite the site of modification
124.	gDNA_16_9_C	GGTTAGAC**C**TTAAGTC	16 nt DNA guide with C in position 9 opposite the site of modification
125.	gDNA_16_10_C	AGGTTAGAC**C**TTAAGT	16 nt DNA guide with C in position 10 opposite the site of modification
126.	gDNA_16_11_C	TAGGTTAGAC**C**TTAAG	16 nt DNA guide with C in position 11 opposite the site of modification

127.	gDNA_16_13_C	TATAGGTTAGAC**C**TTA	16 nt DNA guide with C in position 13 opposite the site of modification
128.	gDNA_16_15_C	CCTATAGGTTAGAC**C**T	16 nt DNA guide with C in position 15 opposite the site of modification

Supplementary Table 2. Data collection and refinement statistics of PliAgo structures.

**Complex**	**PliAgo (Native)**	**PliAgo-OH-gDNA complex**	**PliAgo-P-gDNA complex**	**PliAgo-P-gDNA-Mg^{2+} complex**	**PliAgo (SeMet)**
**PDB code**	7R8F	7R8G	7R8H	7R8J	7R8K
**Data collection**					
Space group	I23	I23	I23	I23	I23
Cell dimensions					
a (Å)	197.94	200.60	199.82	199.19	199.81
b (Å)	197.94	200.60	199.82	199.19	199.81
c (Å)	197.94	200.60	199.82	199.19	199.81
β (°)	90.00	90.00	90.00	90.00	90,00
Resolution (Å)	50–3.10(3.21-3.10)*	50–2.50(2.54-2.50)*	50–2.54(2.59-2.54)*	50–2.70(2.75-2.70)*	50–3.28(3.25-3.28)*
Total reflections	425,622	469,596	868,403	697,735	342,776
Unique reflections	23,413(2,310)*	46,475(2,292)*	43,544(2,179)*	36,116(1,764)*	17,168(1,032)*
Redundancy	18.2(15.7)*	10.1(10.3)*	19.9(20.8)*	19.3(19.2)*	20.0(19.7)*
Completeness (%)	100(100)*	100(100)*	100(100)*	100(100)*	100(100)*
I / σ	24(1.17)*	27.3(1)*	28(1)*	25.6(1.12)*	23.7(2.17)*
$CC^{1/2}$	(0.331)*	(0.344)*	(0.320)*	(0.371)*	(0.699)*
**Refinement**					
Resolution (Å)	49–3.16(3.23-3.16)	45–2.50(2.56-2.50)	45–2.54(2.61-2.54)	45–2.70(2.77-2.70)	47–3.28(3.36-3.28)
R_{work}	0.214(0.349)*	0.211(0.330)*	0.200(0.395)*	0.206(0.349)*	0.220(0.373)*
R_{free}	0.258(0.426)*	0.254(0.384)*	0.239(0.464)*	0.244(0.385)*	0.265(0.388)*

No. of atoms	6,019	6,336	6,408	6,264	6,029
No. of waters	0	35	0	0	0
R.m.s deviations					
Bond length (Å)	0.012	0.009	0.009	0.009	0.013
Bond angles (°)	1.694	1.094	1.102	1.153	1.702
Clashscore	12.44	8.73	7.73	11.6	17.69
Ramachandran favored, %	93.32	90.48	93.87	93.18	84.07
Ramachandran outliers, %	1.31	2.22	0.39	0.26	1.17

Data sets were collected at MacCHESS ID7B2 line, Ithaca, NY

*Highest resolution shells are shown in parentheses
