# Supplementary material for: Programmable RNA targeting by bacterial Argonaute nucleases with unconventional guide binding and cleavage specificity
Source: Nat Commun. 2022 Aug 8;13:4624. doi: 10.1038/s41467-022-32079-5 (PMC9360449; doi:10.1038/s41467-022-32079-5)
Supplement: Supplementary file 3 — Description of Additional Supplementary Files [file 41467_2022_32079_MOESM3_ESM.pdf]

Description of Additional Supplementary Files

File Name: Supplementary Movie 1
Description: The movie shows structural features of PliAgo in comparison with previously studied pAgo proteins.
