# Supplementary figures and images for: Programmable RNA targeting by bacterial Argonaute nucleases with unconventional guide binding and cleavage specificity
Source: Nat Commun. 2022 Aug 8;13:4624. doi: 10.1038/s41467-022-32079-5 (PMC9360449; doi:10.1038/s41467-022-32079-5)

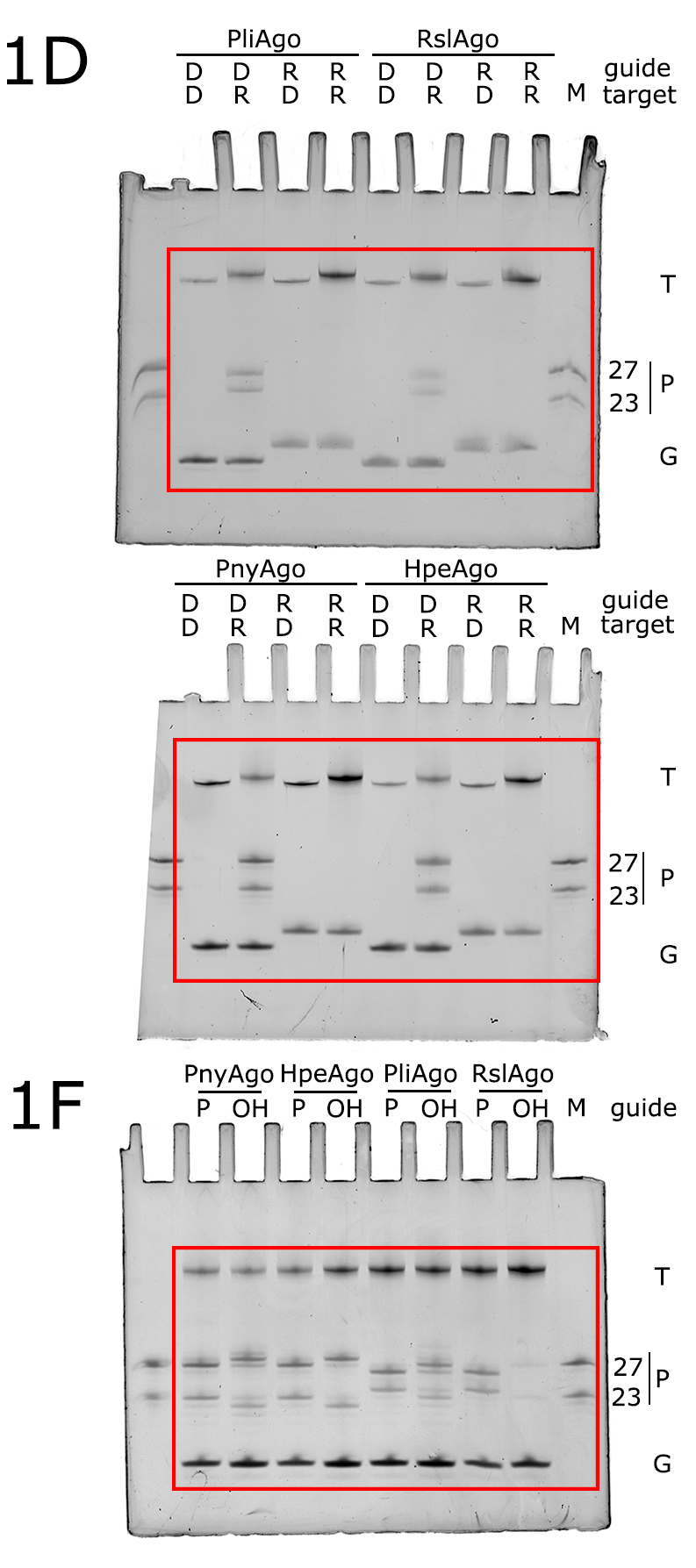

Supplement: Supplementary file 6 — Source Data [file 41467_2022_32079_MOESM6_ESM.zip › 1D+1F.tif]

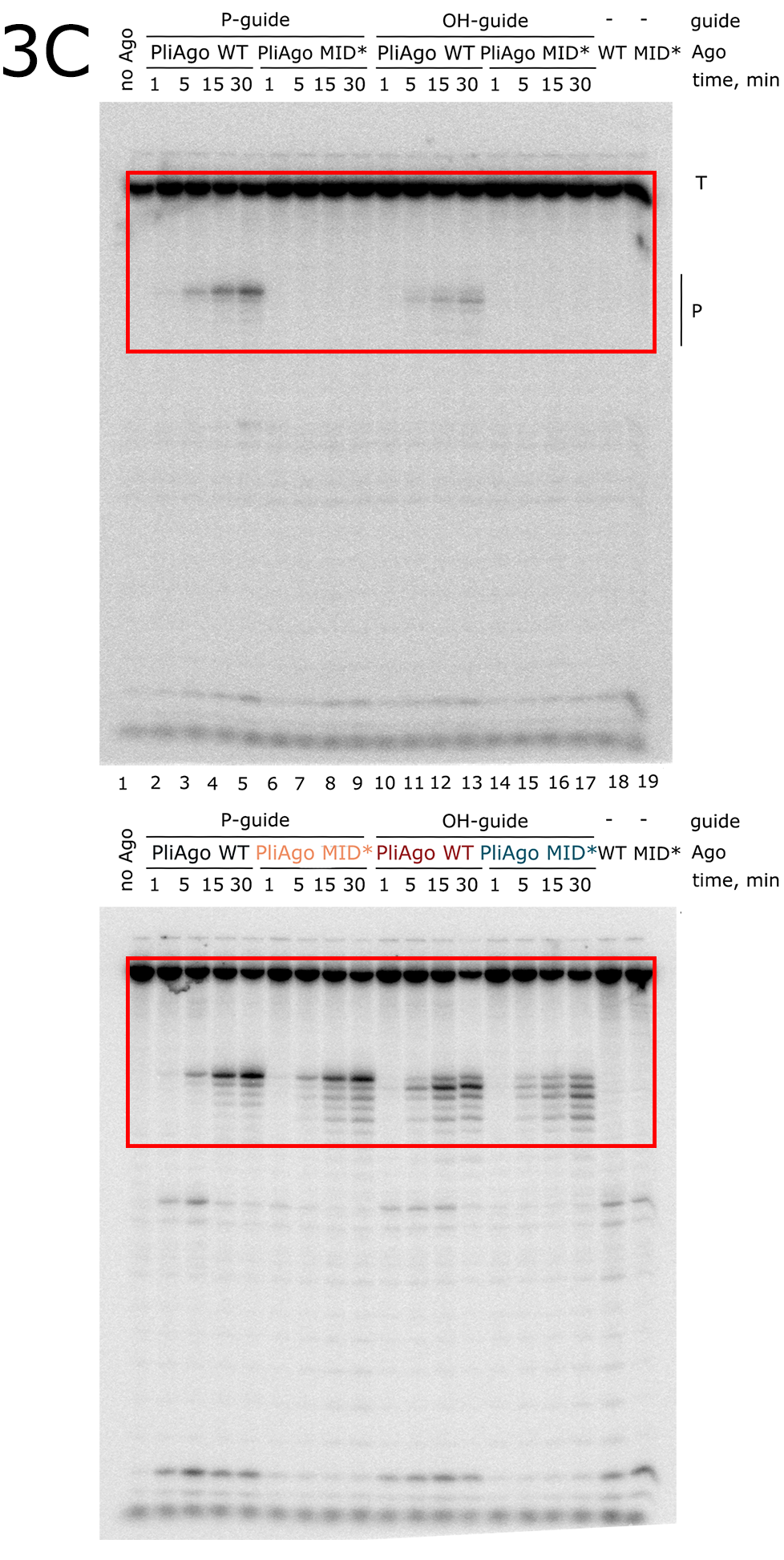

Supplement: Supplementary file 6 — Source Data [file 41467_2022_32079_MOESM6_ESM.zip › 3C.tif]

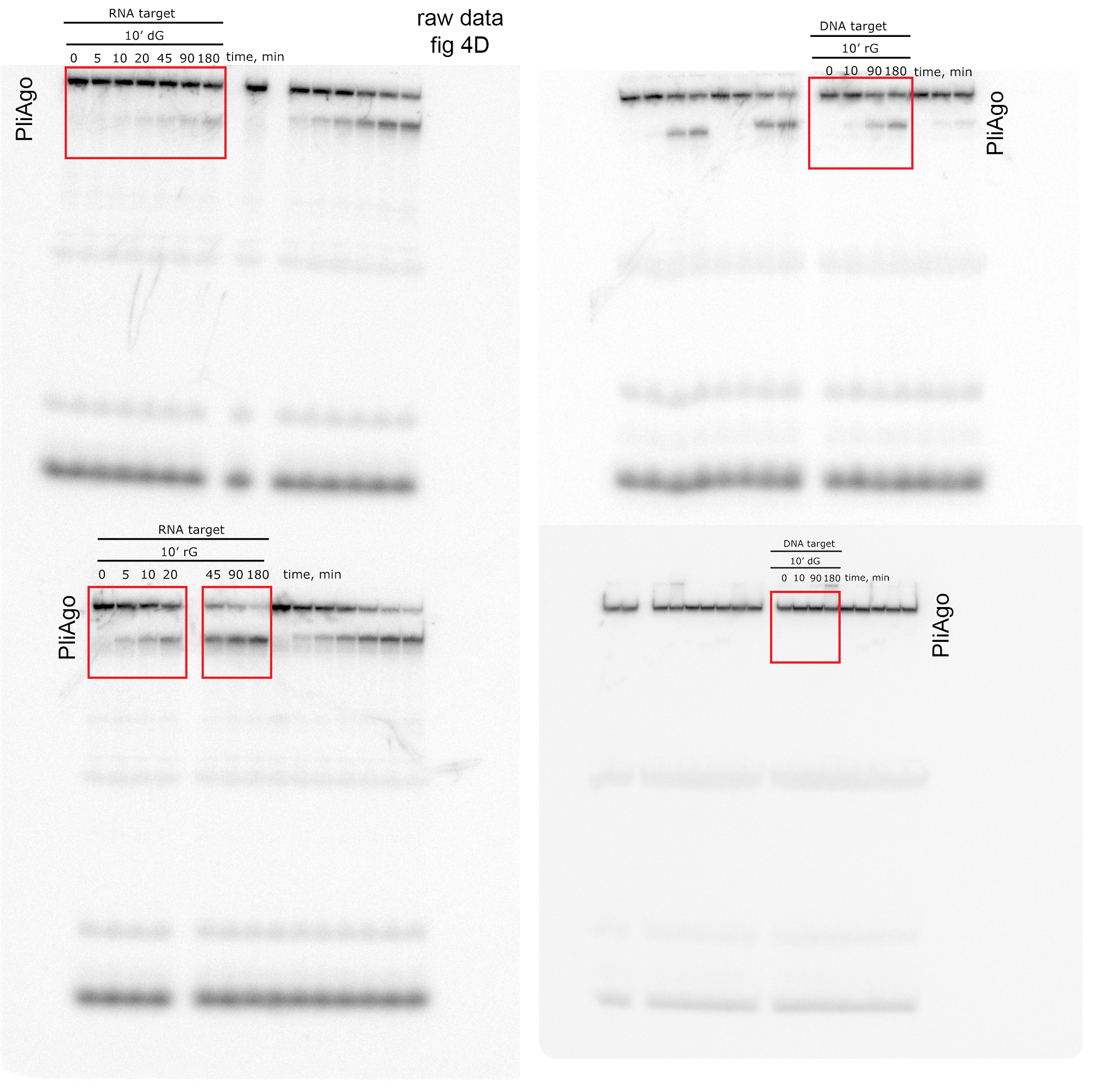

Supplement: Supplementary file 6 — Source Data [file 41467_2022_32079_MOESM6_ESM.zip › 4D.tif]

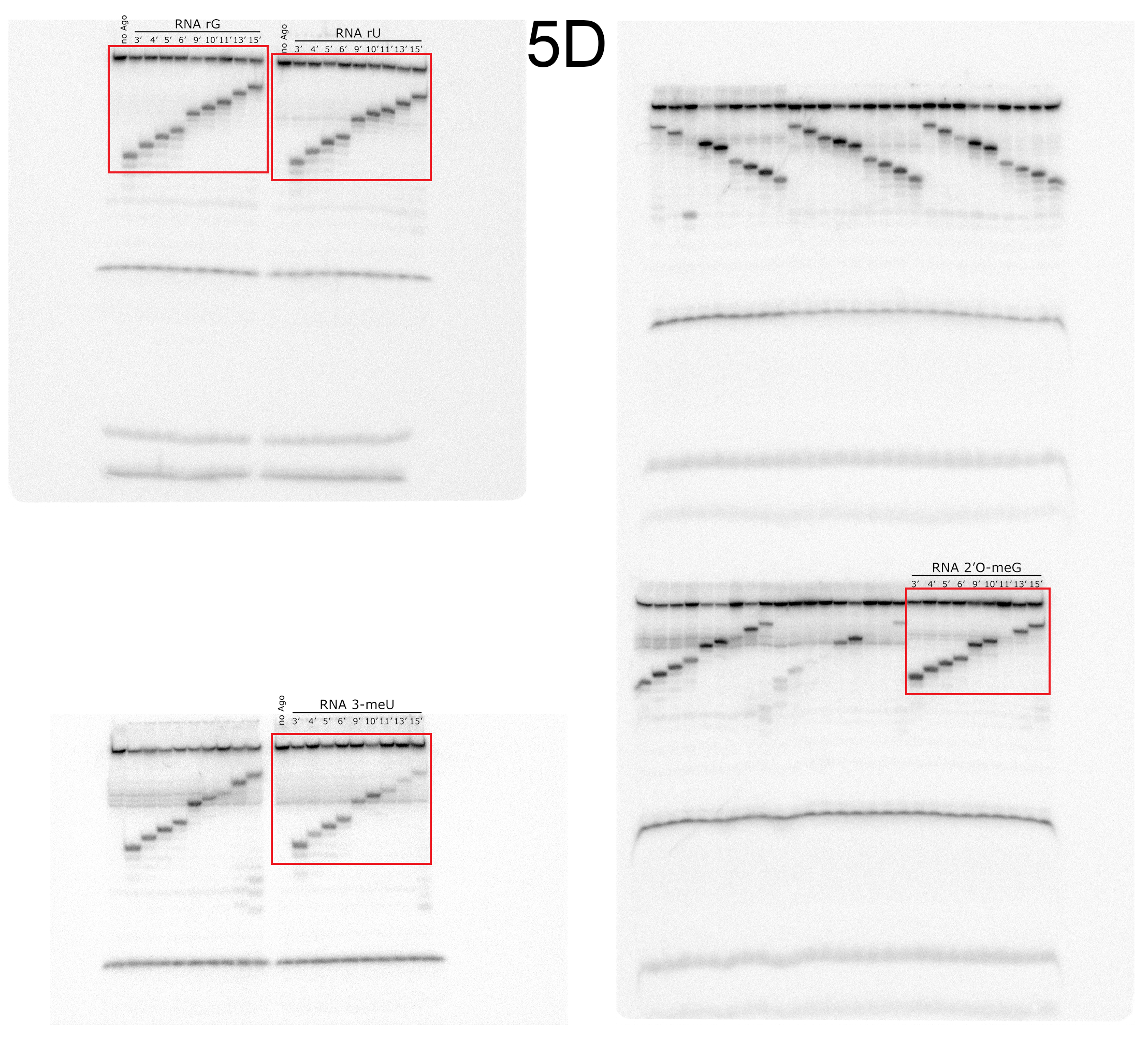

Supplement: Supplementary file 6 — Source Data [file 41467_2022_32079_MOESM6_ESM.zip › 5B.tif]

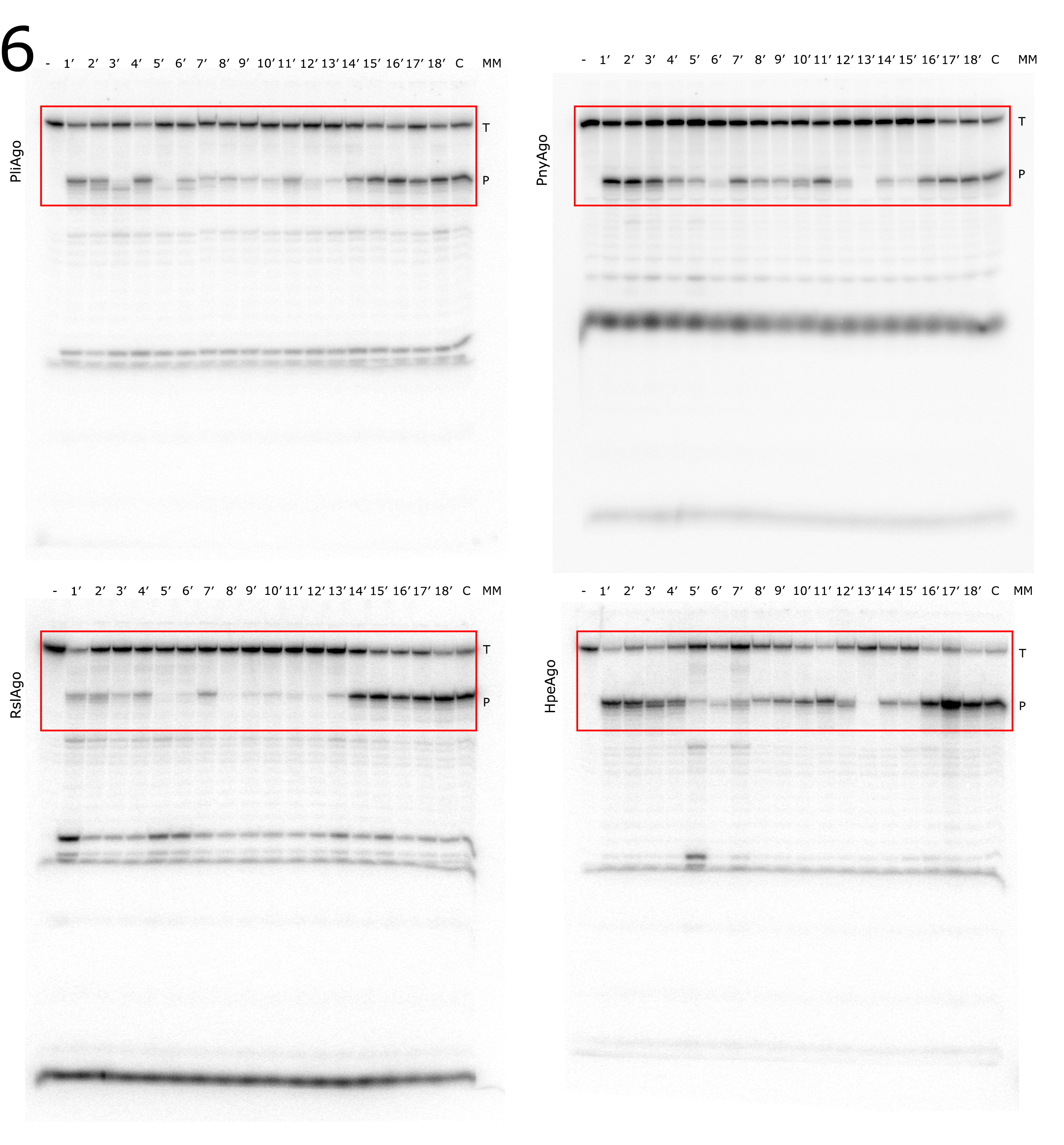

Supplement: Supplementary file 6 — Source Data [file 41467_2022_32079_MOESM6_ESM.zip › 6.tif]

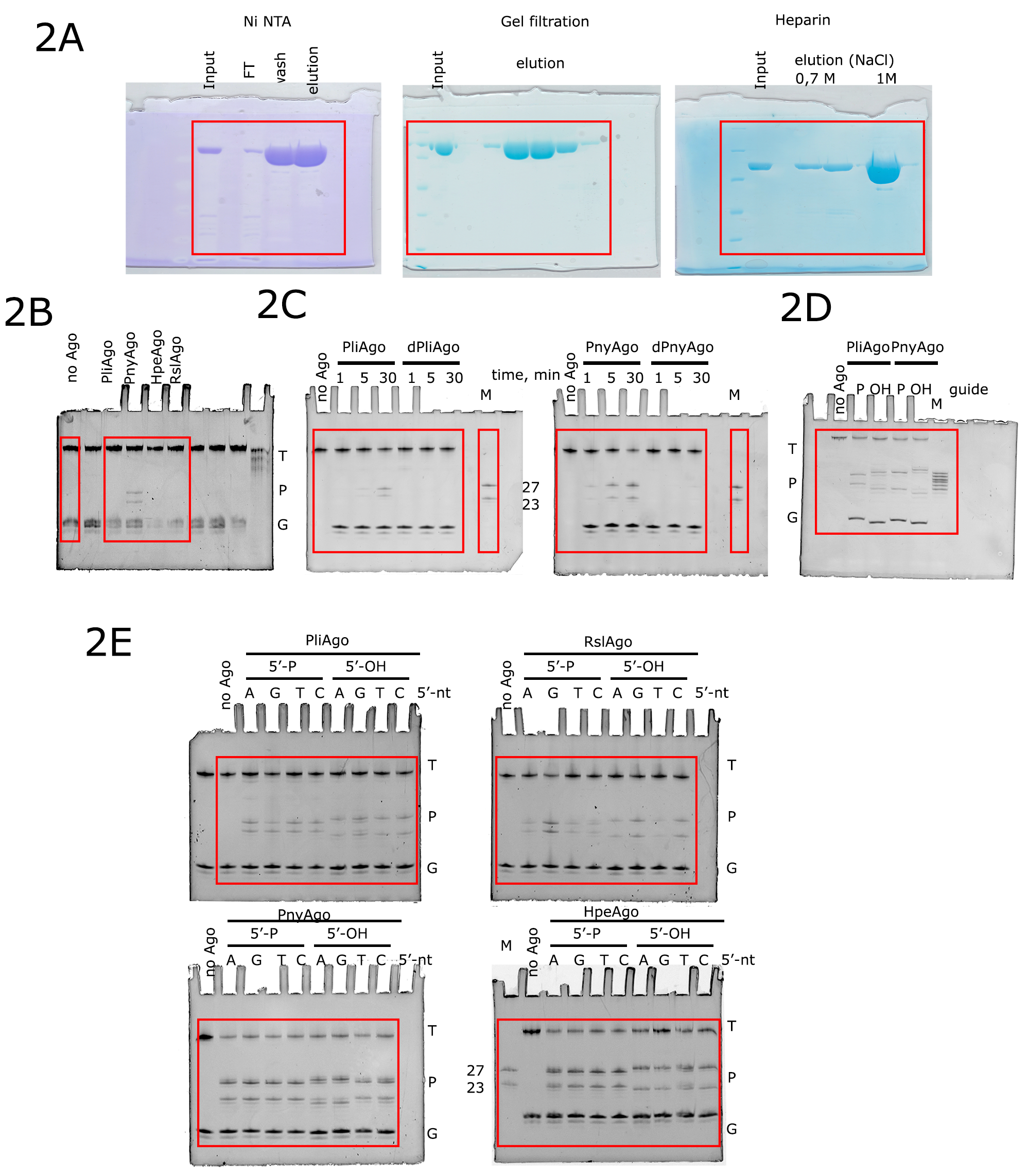

Supplement: Supplementary file 6 — Source Data [file 41467_2022_32079_MOESM6_ESM.zip › S2A+B+C+D+E.tif]

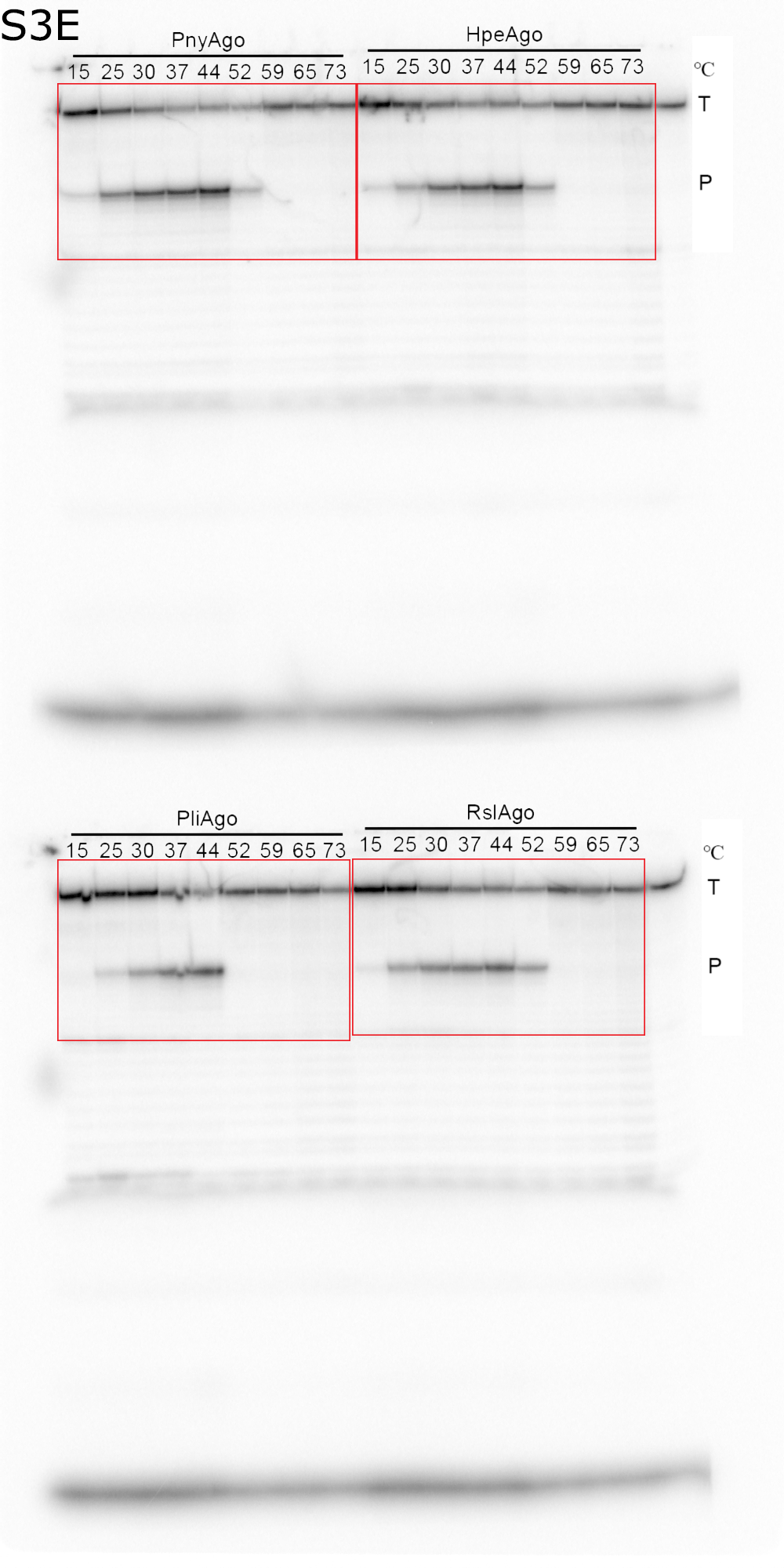

Supplement: Supplementary file 6 — Source Data [file 41467_2022_32079_MOESM6_ESM.zip › S3E.tif]

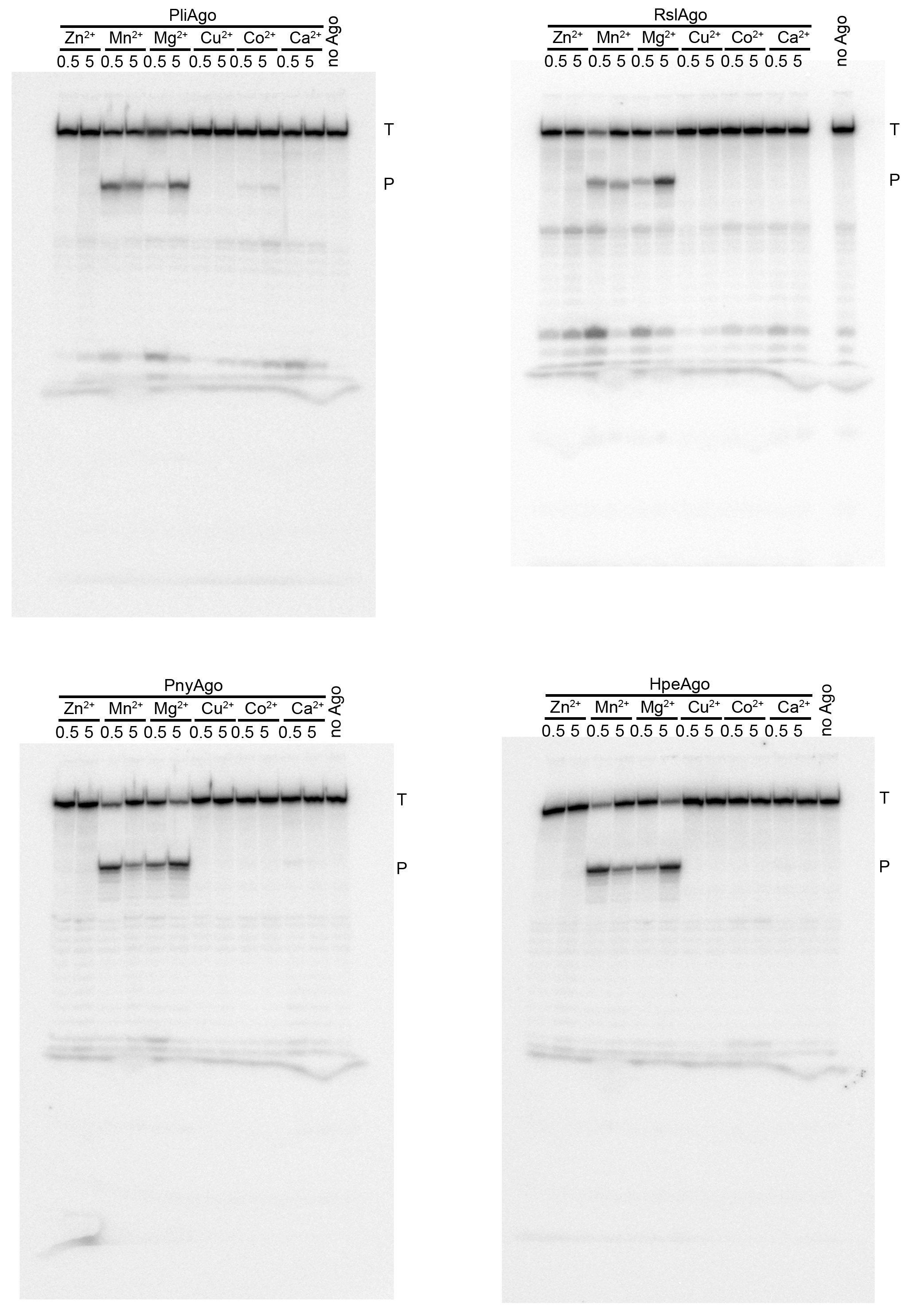

Supplement: Supplementary file 6 — Source Data [file 41467_2022_32079_MOESM6_ESM.zip › S3F.tif]

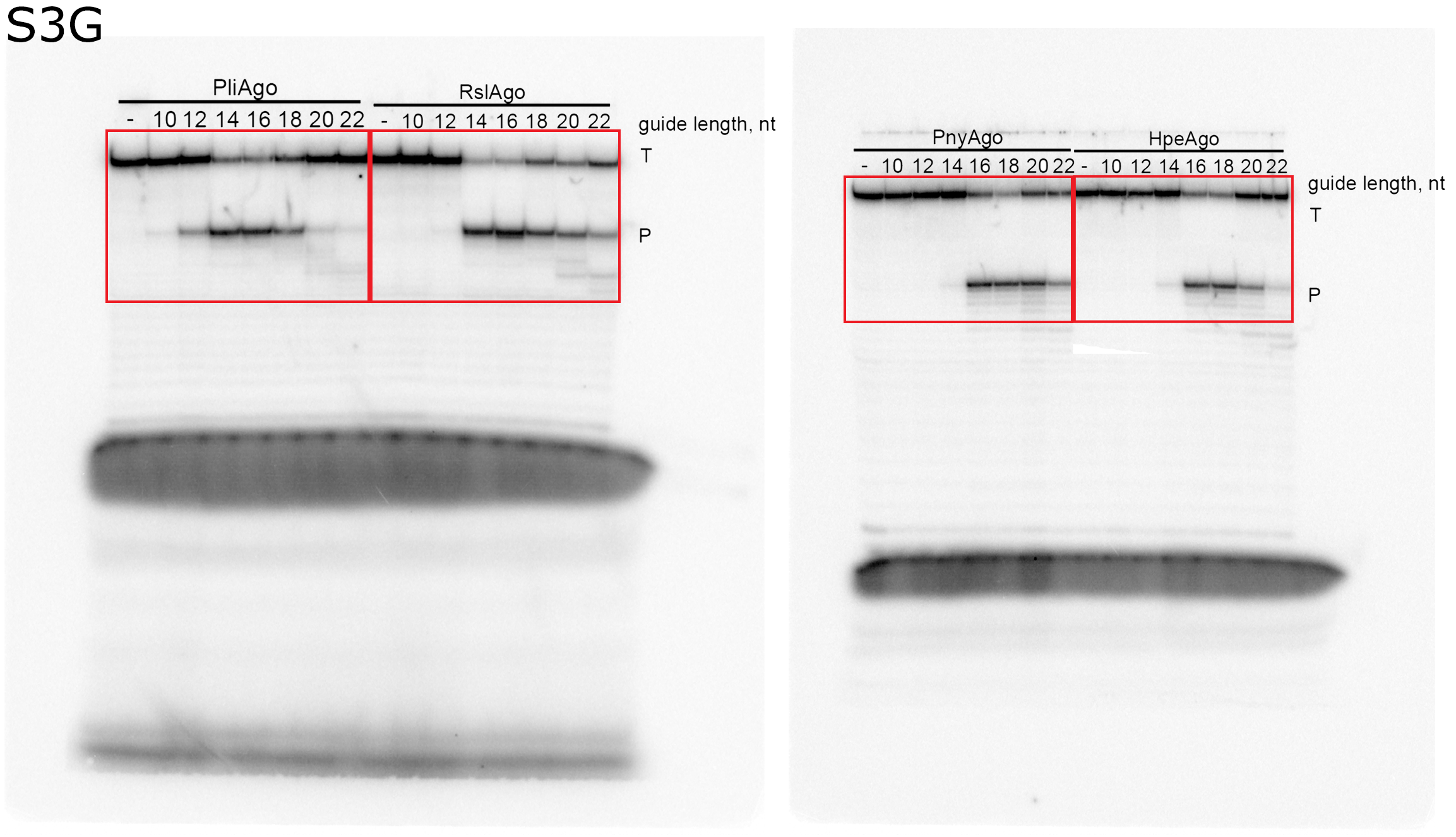

Supplement: Supplementary file 6 — Source Data [file 41467_2022_32079_MOESM6_ESM.zip › S3G.tif]

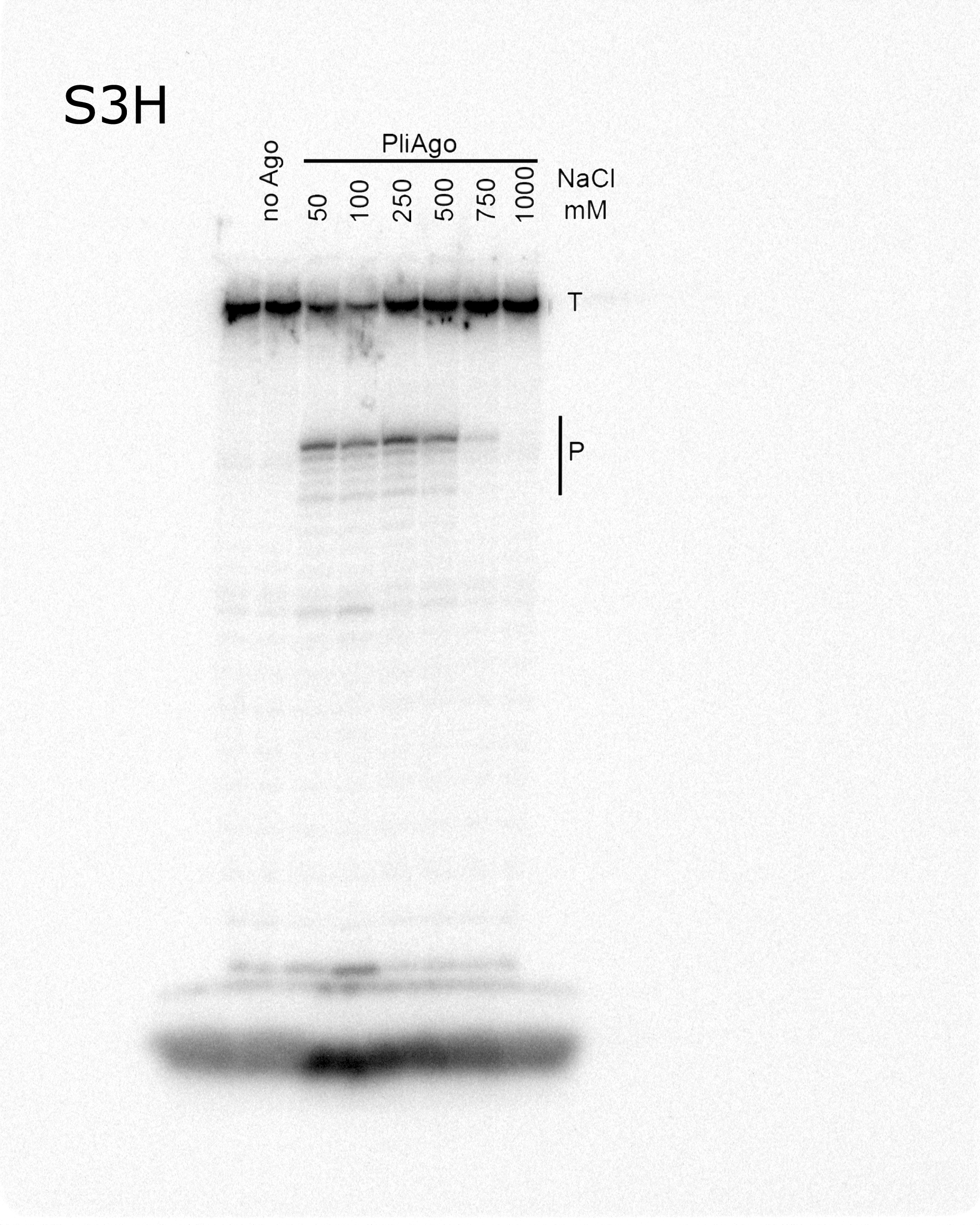

Supplement: Supplementary file 6 — Source Data [file 41467_2022_32079_MOESM6_ESM.zip › S3H.tif]

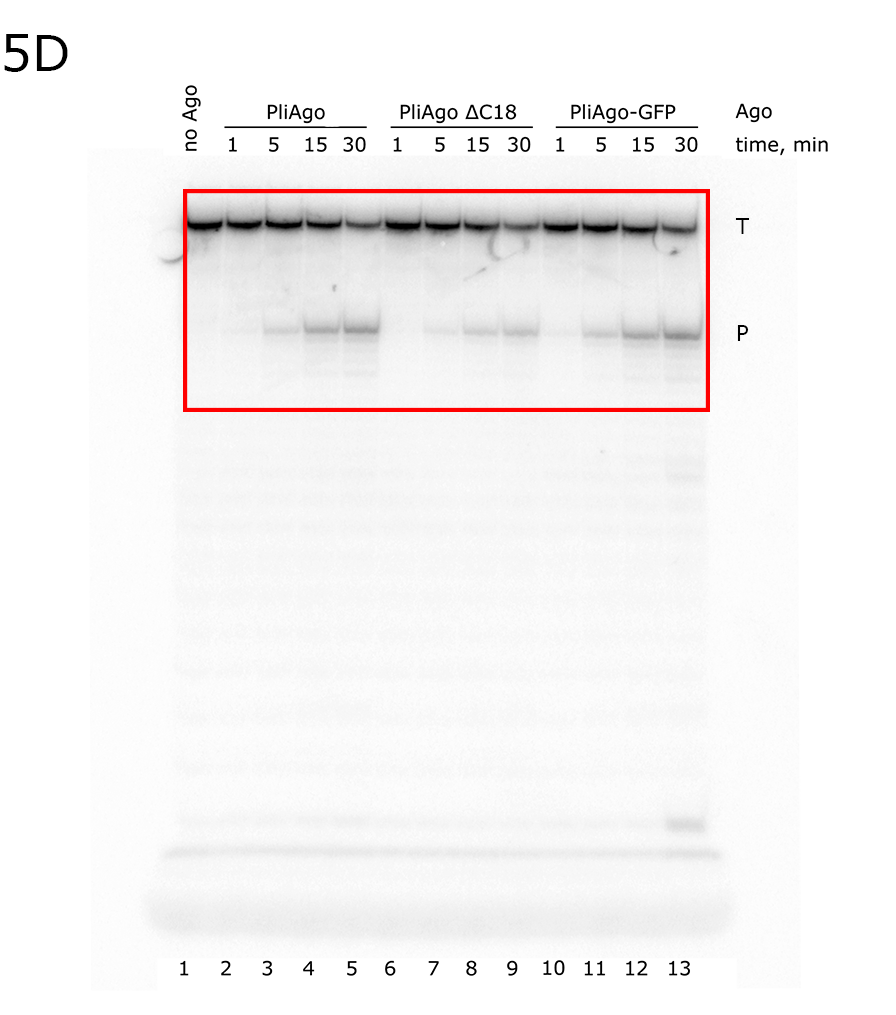

Supplement: Supplementary file 6 — Source Data [file 41467_2022_32079_MOESM6_ESM.zip › S5D.tif]

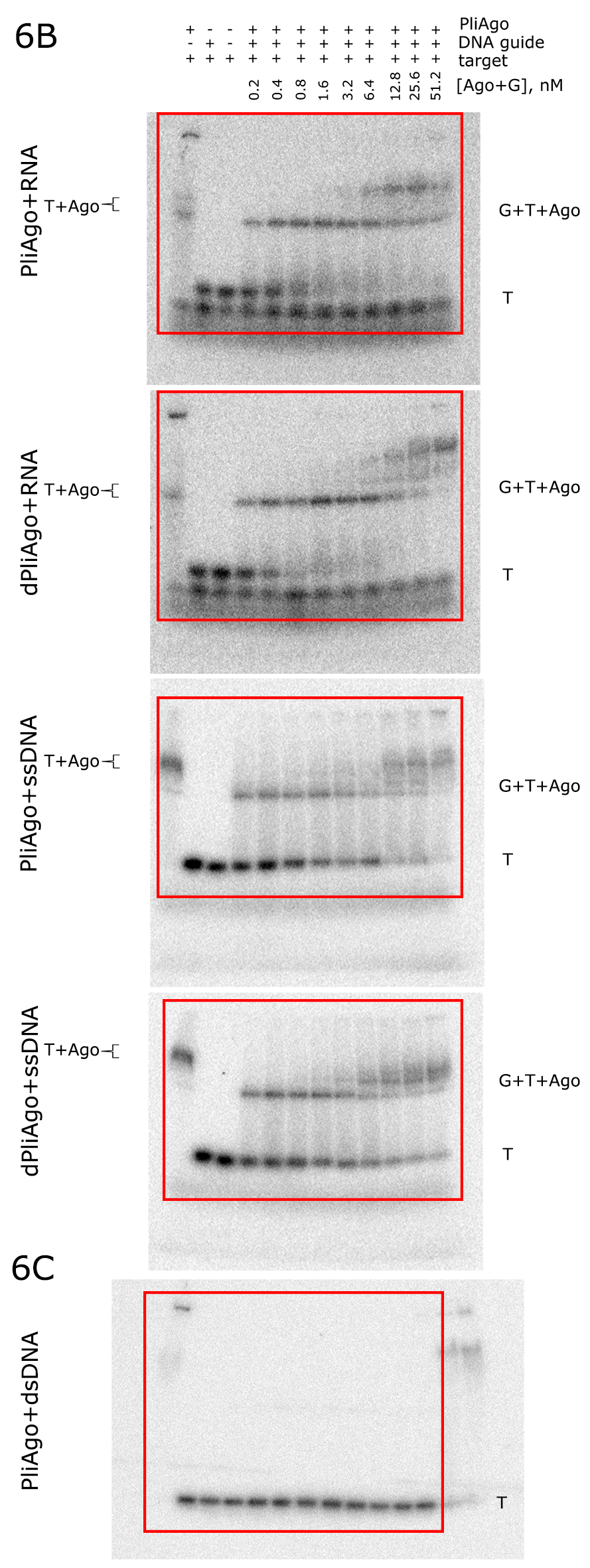

Supplement: Supplementary file 6 — Source Data [file 41467_2022_32079_MOESM6_ESM.zip › S6B.tif]

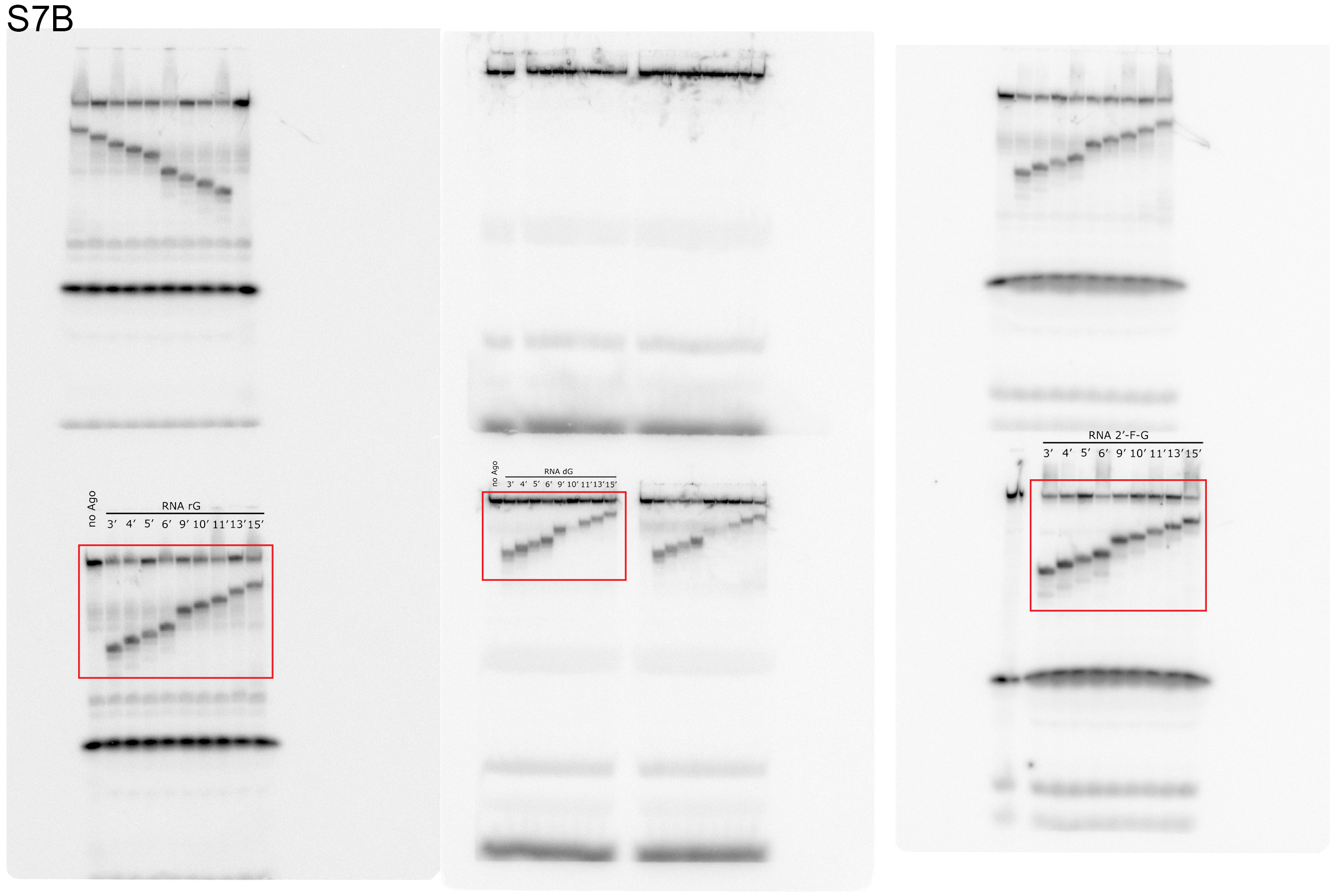

Supplement: Supplementary file 6 — Source Data [file 41467_2022_32079_MOESM6_ESM.zip › S7B.tif]

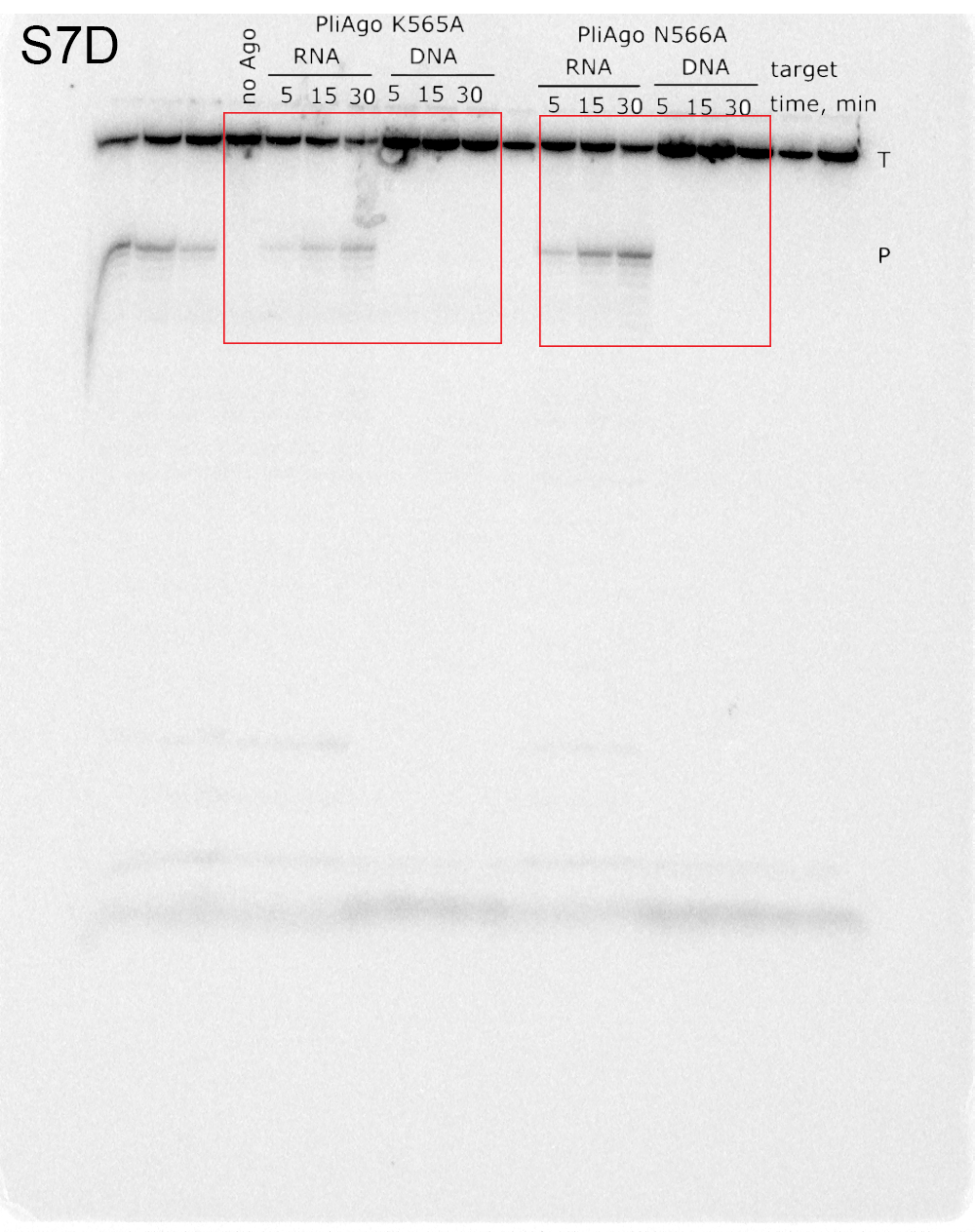

Supplement: Supplementary file 6 — Source Data [file 41467_2022_32079_MOESM6_ESM.zip › S7D.tif]
