# Supplementary material for: Programmable RNA targeting by bacterial Argonaute nucleases with unconventional guide binding and cleavage specificity
Source: Nat Commun. 2022 Aug 8;13:4624. doi: 10.1038/s41467-022-32079-5 (PMC9360449; doi:10.1038/s41467-022-32079-5)
Supplement: Supplementary file 2 — Peer Review File [file 41467_2022_32079_MOESM2_ESM.pdf]

Editorial Note: This manuscript has been previously reviewed at another journal that is not operating a transparent peer review scheme. The manuscript was considered suitable for publication without further review at Nature Communications.
